# Coupling Photochromism and Charge Transport in π‑Extended Arylazo Oligothiophenes and Oligothienoacenes

**DOI:** 10.1021/jacs.6c06319

**Published:** 2026-07-17

**Authors:** Chiara Taticchi, Federico Nicoli, Mattia Zangoli, Filippo Monti, Eugenio Lunedei, Alessandro Turci, Massimiliano Curcio, Pierluigi Mondelli, Mario Caironi, Magda Monari, Massimo Gazzano, Andrea Candini, Alberto Zanelli, Francesca Tinti, Nadia Camaioni, Alberto Credi, Massimo Baroncini, Francesca Di Maria

**Affiliations:** † Dipartimento di Chimica Industriale “Toso Montanari”, Alma Mater Studiorum—Università di Bologna, Via Gobetti 85, 40129 Bologna, Italy; ‡ Center for Light Activated Nanomaterials (CLAN), via Gobetti 101, 40129 Bologna, Italy; § Istituto per la Sintesi Organica e la Fotoreattività, 201838 Consiglio Nazionale delle Ricerche, via Gobetti 101, 40129 Bologna, Italy; ∥ Istituto per lo Studio dei Materiali Nanostrutturati, 9327 Consiglio Nazionale delle Ricerche, via Gobetti 101, 40129 Bologna, Italy; ⊥ Dipartimento di Scienze e Tecnologie Agro-alimentari, 304050 Alma Mater Studiorum—Università di Bologna, Viale Fanin 44, 40127 Bologna, Italy; # Center for Nano Science and Technology, 403543 Istituto Italiano di Tecnologia, Via Rubattino 81, 20134 Milano, Italy; ∇ Dipartimentodi Chimica “G. Ciamician”, Alma Mater Studiorum—Università di Bologna, Via Gobetti 85, 40129 Bologna, Italy

## Abstract

Developing photoresponsive molecular materials that couple efficient charge transport with robust photoswitching remains challenging because structural features enhancing one function often compromise the other. In this study, two homologous series of arylazo oligothiophenes and oligothienoacenes–in which an azo photoswitch is directly conjugated to a π-extended thiophenic scaffold–were systematically investigated. Ultraviolet–visible (UV–vis) spectroscopy combined with theoretical calculations shows that, in solution, π-extension enables *E → Z* photoisomerization using visible light but concurrently lowers the barrier for thermal *Z → E* back-conversion by activating rotational isomerization pathways. Aryl fluorination counteracts this effect by strengthening intramolecular S­(n)···π interactions, extending the *Z*-isomer half-life from minutes to hours. In the solid state, π-extension promotes dense intermolecular packing that inhibits photoisomerization. Introduction of a bulky trityl group reduces packing density, as revealed by single-crystal analysis, restoring *E* ⇆ *Z* photochromism in crystalline thin films and inducing a crystalline-to-amorphous transformation, as evidenced by X-ray diffraction (XRD) and microscopic analysis. The resulting light-driven molecular and morphological reorganization enables reversible, light-induced modulation of electrical conductivity in two-contact planar devices. Space-charge-limited-current measurements show that conductivity changes correlate with isomer-dependent electron mobility properties. Overall, these findings establish π-extended azo-thiophene scaffolds as a versatile platform to couple photochromism and morphological reorganization with charge transport phenomena.

## Graphical abstract

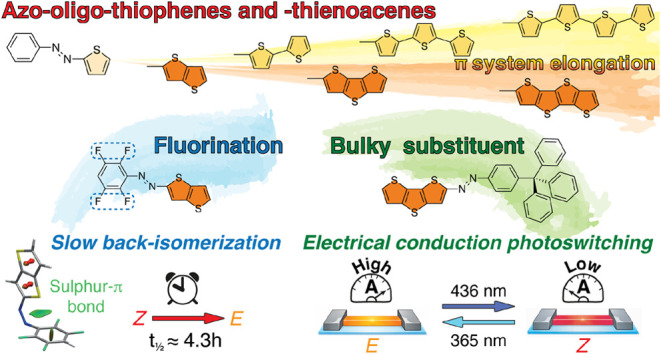

## 1 Introduction

Photochromic compounds are formally defined by their ability to undergo reversible, light-induced changes in their optical absorption spectrum.^1,2^ However, their functional relevance extends well beyond the simple modulation of light absorption. Indeed, through rational (supra)­molecular design, photochromic compounds can be engineered to transduce their light-triggered structural and electronic changes into the reversible modulation of a wide range of molecular and bulk properties.^3,4^ As a result, they have emerged as key tools in the development of advanced light-driven technologies, with applications in data storage,^5–7^ mechanical actuation,^8–12^ molecular motors,^13–16^ solar thermal energy storage,^17–19^ optoelectronics,^20–24^ and precision photopharmacology.^25–27^

Nowadays, within the vast pantheon of photochromic compounds, azo derivatives reign unchallenged due to their straightforward synthesis, structural versatility, and efficient *E* ⇄ *Z* photoisomerization, which entails substantial geometric and electronic reorganization.^28–32^ Substitution with electron-donating or -withdrawing groups and, more recently, replacement of conventional aryl units with heteroaryl rings have proven to be effective strategies to enhance photochromic figures of merit, including visible-light addressability, switching efficiency, fatigue resistance, and persistence of the metastable *Z* isomer.^33–35^ By contrast, although extension of π-conjugation in azo compounds is well-known to red-shift and intensify light absorption in the visible region, it usually entails significant trade-offs in both photoisomerization efficiency and thermal stability of the *Z* isomer.^36–40^ Moreover, extension of π-conjugation typically promotes strong intermolecular interactions, further hindering the already limited photochromic performance of azo compounds in the solid state.^41,42^ As a consequence, π-extended azo compounds have traditionally found application mainly as dyes and pigments, rather than as photochromic materials, in which π-extension has often been regarded as an unfavorable structural feature, and not as a handle to exploit.^43,44^

In parallel, π-conjugated thiophene-based oligomers have emerged as prototypical small-molecule organic semiconductors, featuring well-established structure–property relationships, tunable energy levels, and excellent processability in thin films.^45,46^ Their implementation in organic field-effect transistors, solar cells, and light-emitting devices has demonstrated how subtle variations in backbone structure and solid-state packing can profoundly affect charge transport.^47^

Building on the pioneering work by Wachtveitl and collaborators on the photochromism of arylazo thiophene monomers,^48^ this study investigates two homologous series of arylazo thiophene-based oligomers (Chart 1). These compounds comprise two, three, or four thiophene units, connected either through α-linkages (oligothiophenes) or by ring fusion (oligothienoacenes), thereby extending the thiophene π-conjugation and enabling a systematic assessment of its effect on photochromic properties. Furthermore, by directly coupling an azo photochromic moiety to a π-extended thiophenic scaffold, these architectures comprise functional moieties that can potentially combine photochromism and charge-transport within a single molecular framework. Thus, photoswitching of the effective π-conjugation length between the light-generated *E* and *Z* isomers can provide a direct approach to photomodulate charge transport, a strategy that, to date, has largely been confined to diarylethene-type systems.^49–53^

**Chart 1:**
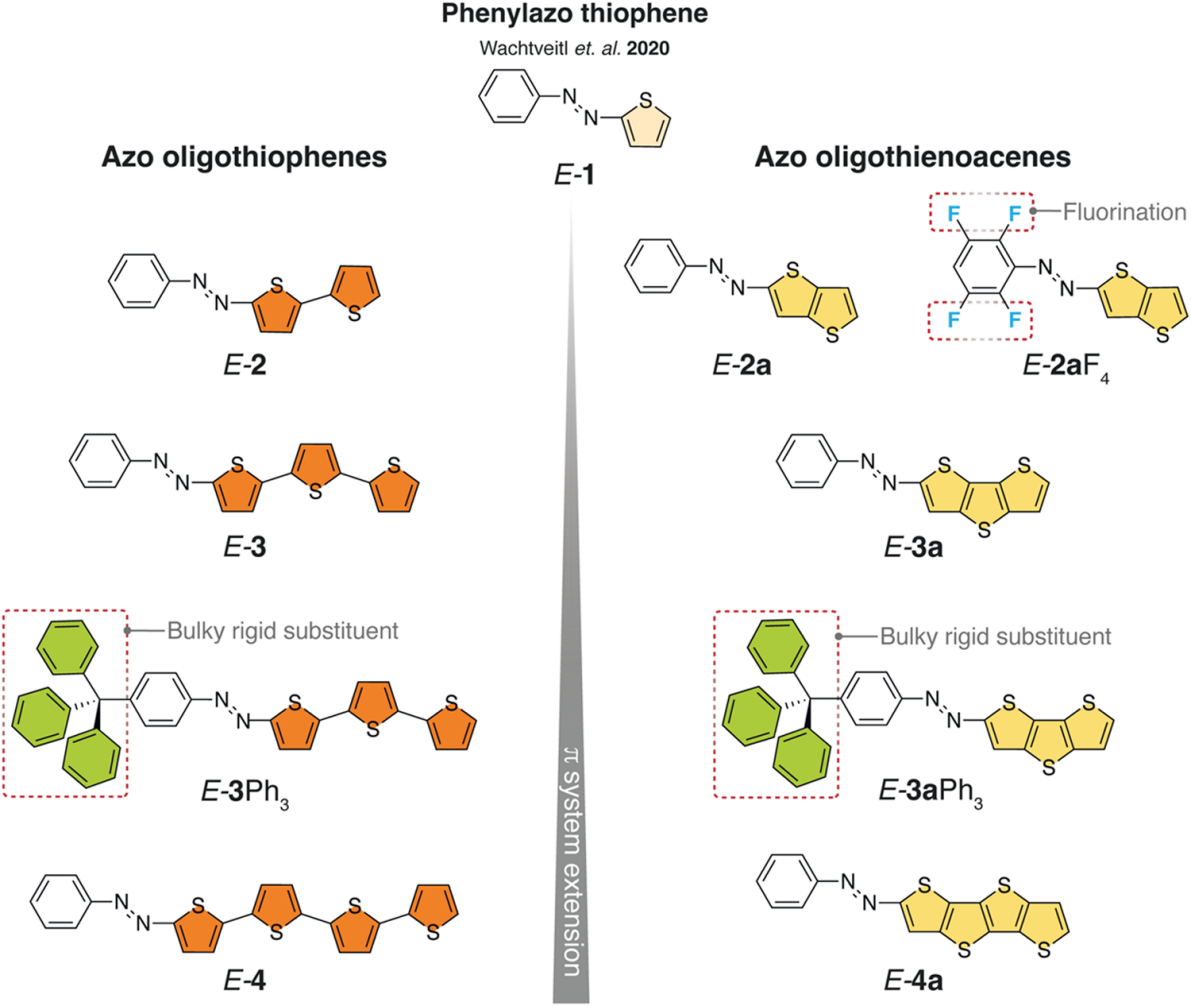
Structures of the Investigated Compounds

The anticipated drawbacks of π-extension on photochromic performances, namely, the destabilization of the *Z* isomer and reduced solid-state photoswitching efficiency, were addressed by complementary design strategies. In particular, in the oligothienoacene series, fluorine functionalization of the aryl moiety (Chart 1, *E*-**2a**F_4_), inspired by prior observations in perfluoroazobenzenes,^54^ markedly increases the half-life of the *Z* isomer and improves photoswitching bistability in solution. In parallel, the introduction of a bulky, shape-persistent trityl group (Chart 1, *E*-**3a**Ph_3_), reminiscent of Onsager-type cross architectures, balances packing density and intermolecular π-stacking, enabling solid-state photochromism while preserving intermolecular electronic coupling.^55,56^

A comprehensive experimental characterization–including ultraviolet–visible (UV–vis) spectroscopy, ^1^H NMR with *in situ* irradiation, cyclic voltammetry, polarized optical microscopy, single-crystal and thin-film X-ray diffraction (XRD), atomic force microscopy (AFM), and electrical characterization–together with density functional theory (DFT), post-Hartree–Fock and multireference calculations, afford detailed structure–property correlations in both solution and solid state, providing new insights into the photochromic behavior of π-extended heteroaryl azo systems and highlighting their potential for advanced photoresponsive and optoelectronic applications.

## 2 Results and Discussion

### 2.1 Synthesis and Molecular Design Optimization

Two homologous series of π-extended heteroaryl azo compounds, based on α-linked oligothiophenes and fused oligothienoacenes (Chart 1), each incorporating two to four thiophene units directly conjugated to a phenylazo moiety, were successfully synthesized *via* azo coupling. This procedure involves α-lithiation of the oligomer precursor with n-butyllithium, followed by *in situ* coupling with the diazonium salt of the aryl moiety (Scheme 1). Full experimental details and compound characterization are reported in the Supporting Information (Scheme S1, and Figures S1–S16).

**Scheme 1:**
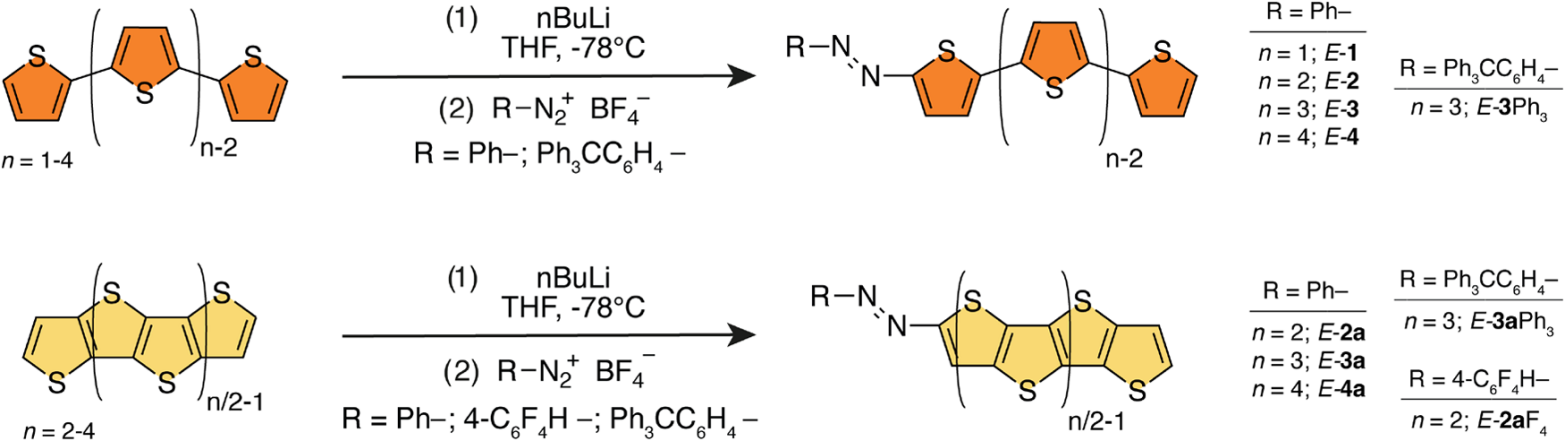
Synthetic Scheme Illustrating the Preparation of the Compounds Investigated in This Work

For both series, reaction yields decrease markedly with increasing oligomer length, likely due to the reduced efficiency of α-lithiation of progressively longer precursors (Table S1). The diazonium counterion also exerts a strong effect on coupling efficiency, with tetrafluoroborate (BF_4_^–^) salts affording the highest yields. In contrast, substituents on the aryl diazonium salt, either electron-withdrawing fluorine atoms or a bulky electron-donating trityl moiety, have only a minor impact. Notably, for the oligothiophene series, a complementary microwave-assisted Suzuki-Miyaura cross-coupling strategy was developed, enabling access to higher homologues with improved yields in shorter reaction times (Scheme S2).

In parallel with increasing oligomer length, the solubility of the resulting azo compounds markedly decreases. The unsubstituted longer analogues of both families (*E*-**3**/**4** and *E*-**3a**/**4a**) present scarce solubility in common organic solvents, limiting their processability.

DFT-optimized ground-state geometries show that the *E* isomers of all investigated compounds adopt a planar conformation between the phenyl and thienyl rings adjacent to the N=N bond. Furthermore, oligothienoacenes (*E*-**2a**–**4a**) feature fully planar thiophenic backbones, whereas the α-linked oligothiophenes (*E*-**2**–**4**) display moderate torsion angles (θ ≈ 22 ± 3°) between adjacent thiophene rings (Table S2 and Figure S17).^57^ In longer members of both series (*E*-**4** and *E*-**4a**), the planar conformation and large surface area of the heteroaromatic backbone promote strong intermolecular interactions, accounting for their poor solubility.^58^ Overall, the oligothienoacene series exhibits superior solubility compared to the α-linked counterparts.

Ground-state calculations indicate that the presence of the trityl substituent in *E*-**3**Ph_3_ and *E*-**3a**Ph_3_ leads to a significant increase in both solvent-accessible surface area and molecular volume (by approximately 70% and 80%, respectively) compared to the unsubstituted analogues *E*-**3** and *E*-**3a**. Furthermore, a reduction in the dipole moment by around 25% is also observed (*i.e*., the magnitude of the molecular dipole moment decreases from 1.184 to 0.897 D in *E*-**3** *vs E*-**3**Ph_3,_ and from 0.984 to 0.717 D in the case of *E*-**3a** *vs E*-**3a**Ph_3_). Hence, the increased solubility likely arises from the combined effects of enhanced solvent–solute interactions and solid-phase destabilization imparted by the rigid, star-like geometry of the trityl moiety.^59^

### 2.2 Photophysics of the *E* and *Z* Isomers in Solution and Theoretical Insights

All spectroscopic measurements were performed in air equilibrated dichloromethane solution (see Supporting Information for details). As shown in Figure 1, the model compound *E*-**1** exhibits a UV–vis absorption spectrum featuring an intense π–π* band centered at ≈350 nm, shouldered by a weaker n−π* band at ≈450 nm.^48,60^ In contrast, the *E* isomers of both oligomeric series display broad absorption profiles that progressively red-shift with increasing oligomer length. The bathochromic shift is more pronounced in the α-linked series (Figure 1a) than in the fused analogues (Figure 1b) due to the higher π-electron count per thiophene unit in the former (6π vs 4π electrons).^61^ Across both series, the increase of bandwidth and molar absorptivity (ε) values reflects the enhanced light-harvesting capability of extended π systems.

**Figure 1:**
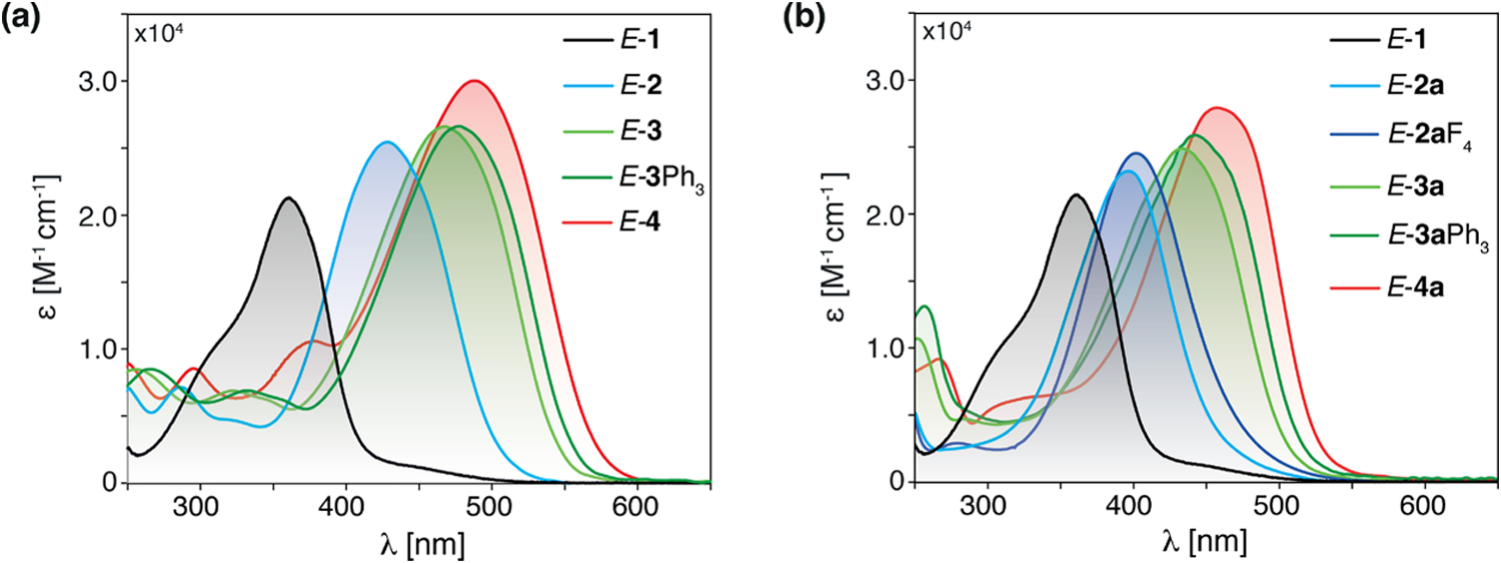
UV–vis absorption spectra in air equilibrated CH_2_Cl_2_ of the *E* isomers of (a) arylazo oligothiophenes, and (b) arylazo oligothienoacenes.

The introduction of electron-withdrawing fluorine atoms (*E*-**2a**F_4_) or the bulky trityl group on the aryl moiety (*E*-**3**Ph_3,_ and *E*-**3a**Ph_3_) induces an additional ≈20 nm red shift relative to parent compounds *E*-**2a**, *E*-**3** and *E*-**3a** (Figure 1a,b).

TD-DFT and STEOM-DLPNO–CCSD calculations reveal that the featureless absorption spectra in both oligomeric series result from the overlap between the n−π* and π–π* transitions (Figure S18), a distinctive feature of azo compounds pertaining to the aminoazobenzene family according to Rau’s classification.^62^ The weak n−π* band originates from the symmetry-forbidden S_0_ → S_1_ transition, involving the lone pairs and the π* orbitals of the N=N azo group. Accordingly, its energy is marginally affected by π-extension. In contrast, the more intense π-π* band–corresponding to the symmetry allowed S_0_ → S_2_ transition and primarily involving a HOMO → LUMO excitation–is strongly influenced by conjugation length (Table S3). Elongation of the thiophene backbone results in a slight stabilization of the LUMO, which remains localized on the azo fragment, and in a progressive destabilization and delocalization of the HOMO across the thiophene backbone (Figures S19 and S20). For both series of compounds, the narrowing of the HOMO–LUMO energy gap upon π-extension: (i) lowers the S_0_ → S_2_ transition energy, (ii) enhances its charge-transfer character, and (iii) leads to higher oscillator strengths. In addition to π-extension, substituents further modulate the frontier orbital energies and, consequently, the optical gap. Fluorination in *E*-**2a**F_4_ lowers both frontier orbital energies compared to *E*-**2a**, whereas aryl functionalization with trityl in *E*-**3**Ph_3_ and *E*-**3a**Ph_3_ slightly destabilizes the HOMO compared to unsubstituted *E*-**3** and *E*-**3a**, leading in both cases to a narrowing of the HOMO–LUMO gap (Figures S21–S23).

Cyclic voltammetry (CV) measurements corroborate these trends, showing only minimal changes in reduction potentials while exhibiting progressively lower oxidation potentials with increasing π-extension. Accordingly, the redox gap decreases along the series and is further reduced by functionalization (Table S5).

Irradiation of the *E* isomers of both series with light of appropriate wavelength induces *E* ⇆ *Z* photoisomerization (Figures S30–S39). The UV–vis spectra of the corresponding *Z* isomers (Figure 2a,b)–reconstructed via Fischer’s method and validated at the TD-ωB97M-D4/def2-TZVP level (see SI and Figure S18)–present spectrally resolved n−π* and π–π* bands. In the *Z* isomers, loss of coplanarity reduces π-conjugation and induces a hypsochromic shift of the π–π* band relative to the *E* form. In contrast, the n−π* band remains centered at ≈450 nm for all compounds, regardless of oligomer length.

**Figure 2:**
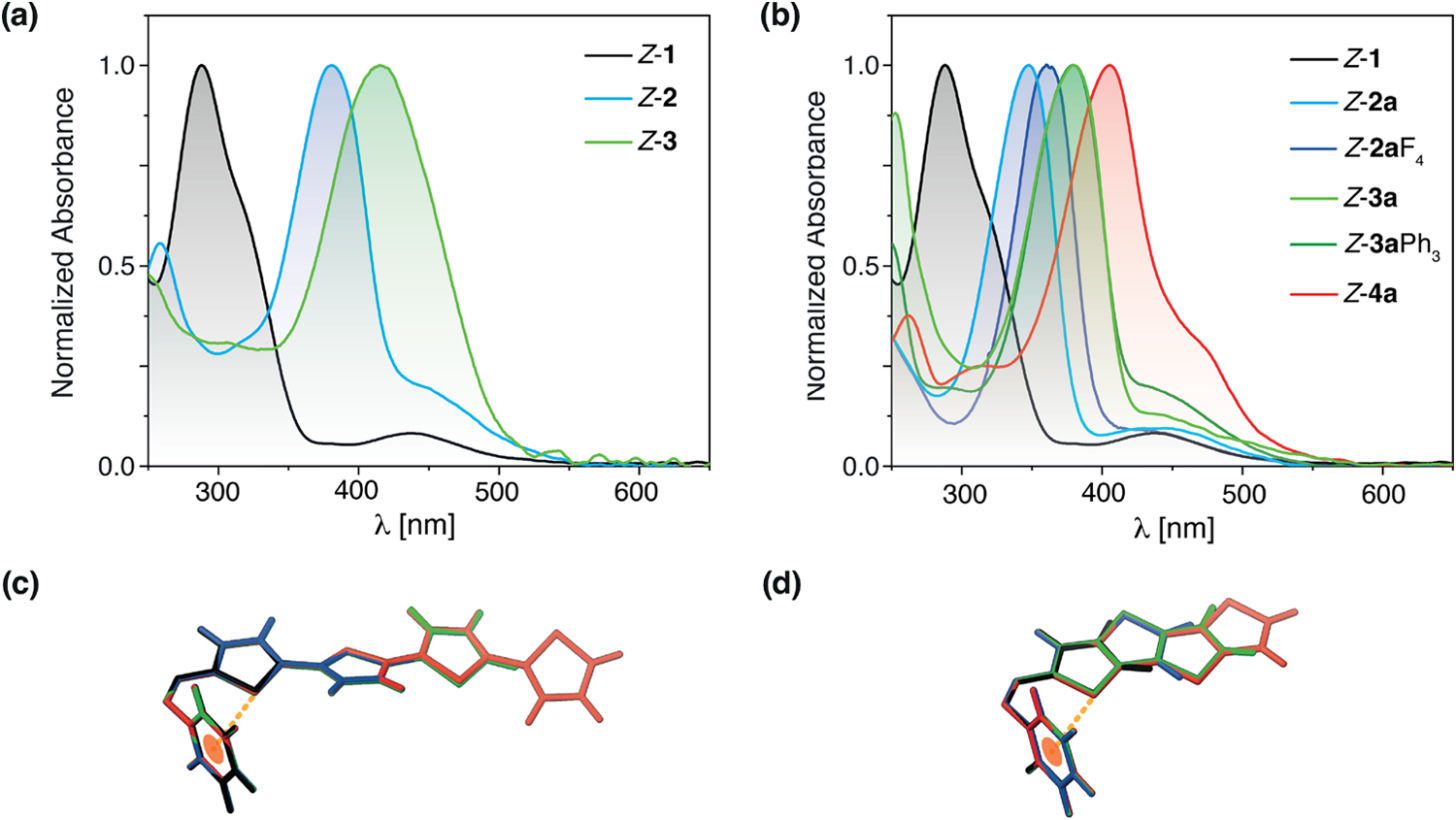
Reconstructed UV–vis absorption spectra in air equilibrated CH_2_Cl_2_ and DFT-optimized ground-state geometries of the *Z* isomers of: (a, c) phenylazo oligothiophenes, (b, d) arylazo oligothienoacenes. The *Z*-**4** spectrum could not be reconstructed due to fast thermal *Z* → *E* back-isomerization. Ground-state geometries overlays were generated by maximizing the overlap between heavy atoms of the phenyl–N=N–thienyl fragment. Color legend: *Z*-**1**, black; *Z*-**2** and *Z*-**2a**, cyan; *Z*-**3** and *Z*-**3a**, green; *Z*-**4** and *Z*-**4a**, red. The intramolecular S­(n)···π interaction is indicated in orange.

DFT calculations further indicate that the *Z* isomers of both series adopt a T-shaped ground-state conformation, with the phenyl ring lying nearly orthogonal to the thiophene plane (Figure 2c,d), stabilized by intramolecular S­(n)···π interactions.^63^ This conformational arrangement is experimentally supported by ^1^H NMR spectra of the *Z* isomer of selected derivatives (**2a**F_4_, **3a**Ph_3_), obtained by *in situ* irradiation in the NMR probehead (Figures S28 and S29), which exhibit downfield thienyl resonances consistent with anisotropic effects of azo and phenyl ring currents, as predicted by GIAO–DFT (Table S6).^64^

### 2.3 Photochromism in Solution

Upon exhaustive irradiation at 365, 405, and 436 nm (main Hg lamp lines isolated with Δλ = 10 nm interference filters), all compounds reach a wavelength-dependent photostationary state (PSS) composition (in the following, these are referred to as PSS_365_, PSS_405_, and PSS_436_, respectively), which exhibits progressively lower *Z*/*E* ratios with increasing oligomer length (Figure 3a and Table S7). Remarkably, and in contrast to parent compound **1** ^48^ and most azo derivatives,^62^ irradiation with visible light (in the range 405–450 nm) yields PSSs enriched in the *Z* isomer, whereas UV light favors accumulation of the *E* isomer. The fused oligothienoacene series exhibit PSS with a higher fraction of the *Z* isomer compared to their α-linked counterparts. Furthermore, they exhibit higher quantum yields for both *E* → *Z* and, particularly, *Z* → *E* photoisomerization processes compared to model compound **1** (Figure 3a).

**Figure 3:**
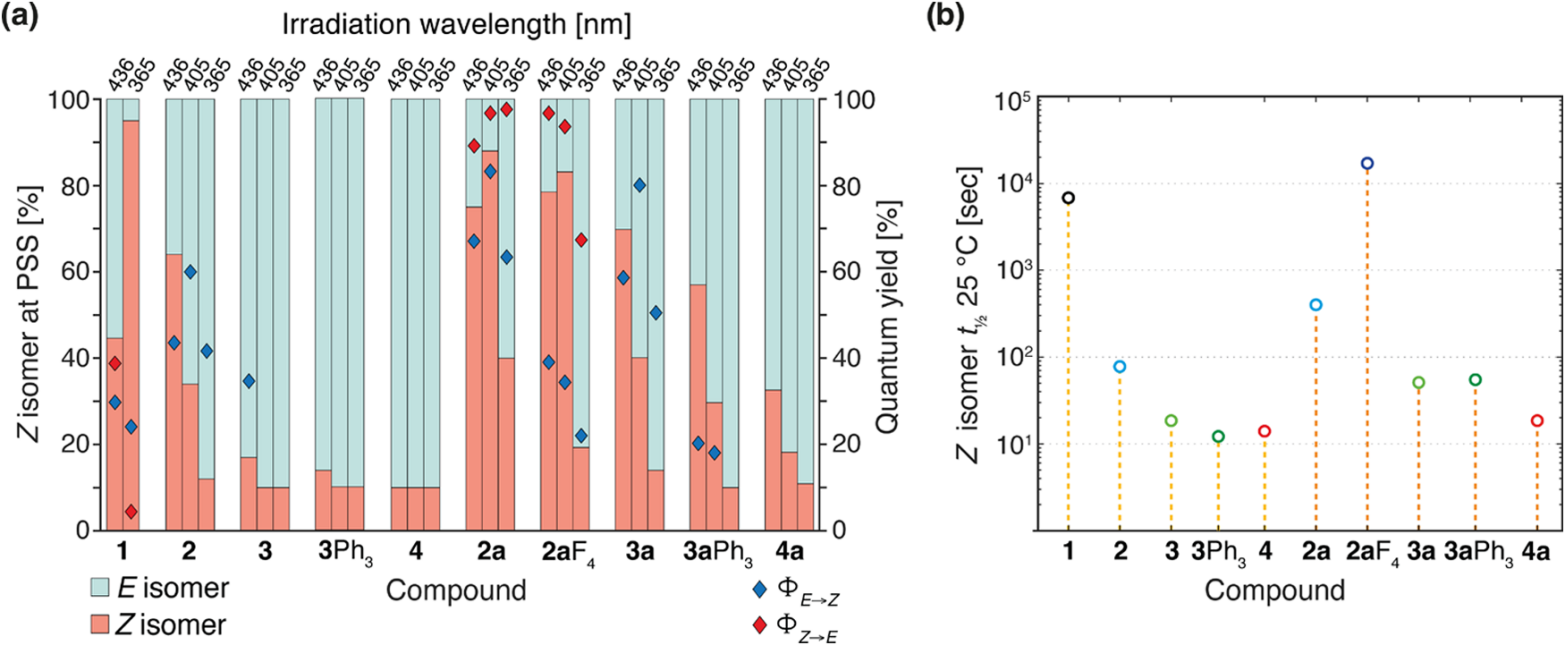
(a) Bar plot of the *Z*-isomer percentage at the photostationary state (PSS) and quantum yield for *E*→*Z* (Φ_*E*→*Z*_ blue diamonds) and *Z*→*E* (Φ_*Z*→*E*_ red diamonds) photoisomerization reactions (%) as a function of irradiation wavelength. (b) Semilogarithmic plot of the *Z* isomers half-life in the dark at 25 °C. Solvent: air equilibrated CH_2_Cl_2_.

For all investigated compounds, the *Z* isomer is metastable and undergoes a thermally activated *Z → E* back-isomerization that follows first-order kinetics (Figures S30–S39). The *Z* isomer half-life (*t*__1_/_2__) decreases sharply–from several hours to seconds–as the number of thiophene units increases, indicating a strong correlation between the extent of π conjugation and the thermal relaxation rate (Figure 3b). Notably, the very fast *Z* → *E* back-isomerization in compounds **3**/**4** and **4a** leads to PSSs whose composition depends strongly on light intensity. This accounts for the low *Z/E* ratios at PSS in the longer congeners of both series and, in turn, limits the accuracy of the isomerization quantum yield values and, for compound **4**, prevents the determination of the *Z*-isomer absorption spectrum.^65^

Ground state DFT and post-HF calculations show that the delocalization of the HOMO over the thiophenic backbone reduces the double-bond character of the azo linkage (Table S3, and Figures S19, and S20), justifying the lowering of the activation barrier for *Z* → *E* thermal back conversion in the extended congeners. Notably, CASSCF/NEVPT2 calculations, performed on selected compounds (Figures S24 and S25) reveal that thermal *Z* → *E* back-isomerization in azothiophenes occurs predominantly through a rotational pathway (i.e., torsion around the N=N azo bond) rather than exclusively via inversion, as previously reported for this class of compounds (see Table S4 and SI for further discussion).^60^

As shown in Figure 3, trityl substitution in both **3**Ph_3_ and **3a**Ph_3_ does not significantly affect photochromic characteristics compared to **3** and **3a**. In contrast, aryl fluorination proved to be an effective molecular handle to control the *Z* isomer persistence and to improve the compositional selectivity of the accessible PSSs.

As reported in Figure 3b, the fluorinated derivative **2a**F_4_ exhibits a remarkable increase in the *Z* isomer half-life compared to **2a** (4.3 h vs 7.2 min, respectively). In addition, **2a**F_4_ presents improved bistability under alternating UV (365 nm) and visible (405 or 436 nm) irradiation (Figure 3a), and also noteworthy photoswitching employing exclusively visible light wavelengths (see Table S7).

Ground state DFT calculations reveal that in *Z*-**2a**F_4_ the enhanced electron-withdrawing character of the aryl moiety generates a larger region of positive ESP above and below the aromatic ring, compared to **2a**, strengthening S­(n)···π interactions (Figures S26 and S27). Consistently, DFT calculations at the ωB97M-D4/def2-TZVP level estimate that the difference in the Gibbs free energy between the *Z* and *E* conformational minima is just 7.3 kcal/mol for **2a**F_4_ vs more than 11 kcal/mol for **2a**. This reduced Δ*G* indicates a greater stabilization of the *Z* isomer of **2a**F_4_ mainly due to stronger S­(n)···π interactions, which must be broken along the thermal *Z* → *E* pathway and likely underlie the markedly slower back-isomerization rate of *Z*-**2a**F_4_ compared to *Z*-**2a**.

#### 2.3.1 Photochromism in the Solid State

The solid-state photochromic behavior of the synthesized compounds was investigated by UV–vis spectroscopy on thin films obtained by spin-coating 10^–3^ M chloroform solutions of the *E* isomers onto quartz substrates (see SI). Among all investigated compounds, only *E*-**3a**Ph_3_ and *E*-**3**Ph_3_ yield films of good optical quality (Figures 4, and S43–S44). In contrast, all other derivatives displayed poor film morphology, indicating that trityl functionalization significantly enhances the processability of these materials. Despite the improved film quality of both trityl-substituted derivatives, only *E*-**3a**Ph_3_ thin films show reversible spectral changes upon light exposure. The absence of photochromism in thin films of *E*-**3**Ph_3_ likely stems from the stronger packing of the more π electron rich α-linked oligothiophene scaffold, which overrides the steric hindrance introduced by the trityl group.

Pristine thin films of *E*-**3a**Ph_3_ (Figure 4a, black trace) upon exhaustive irradiation at 436 nm display UV–vis spectral changes closely resembling those observed in solution, indicative of the formation of a PSS_436_ enriched in the *Z* isomer (Figure 4a, red trace). Subsequent irradiation at 365 nm drives the photoisomerization toward PSS_365_, composed almost quantitatively of the *E* isomer (Figure 4a, blue trace). The presence of well-defined isosbestic points and the absence of induction periods along both photoisomerization processes are consistent with a clean, two-state photochromic interconversion, suggesting weak intermolecular coupling and a homogeneous morphology of the film.^66,67^

**Figure 4:**
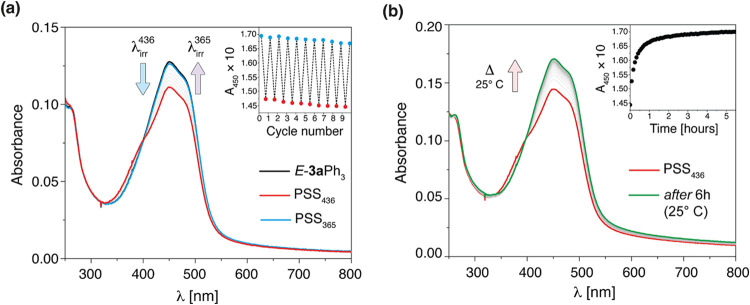
(a) UV–vis absorption spectra of a spin-coated film of *E*-**3a**Ph_3_ (thickness ≈ 60 nm, spin-coated from a 1 mg/mL solution at 1500 rpm): before (black trace), after exhaustive irradiation at 436 nm for 20 min (PSS_436_, red trace), and after subsequent exhaustive irradiation at 365 nm for 20 min (PSS_365_, blue trace). Spectra acquired at intermediate times during 436 nm irradiation are shown in light gray; inset: absorbance variation at 450 nm over repeated cycles of alternate irradiation at 436 nm (20 min, blue circles) and 365 nm (20 min, red circles); (b) UV–vis absorption spectra of a spin-coated film of *E*-**3a**Ph_3_ (thickness ≈ 60 nm) after exhaustive irradiation at 436 nm for 20 min (PSS_436_, red trace) and after 6 h at 25 °C in the dark (green trace). Spectra acquired at intermediate times are shown in light gray; inset: absorbance variation at 450 nm. Light intensity at 365 and 436 nm: ≈20 mW·cm^–2^.

Assuming identical molar absorption (ε) for the *E* and *Z* isomers of **3a**Ph_3_ in thin film and solution (Table S7), the calculated solid-state *Z/E* ratios are 15:85 at PSS_436_ and 1:99 at PSS_365_.

The lower fraction of *Z* isomer at PSS_436_ in the film compared to CH_2_Cl_2_ solutions (*Z*/*E* of 15:85 vs 57:43, Figure 3a) reflects the impact of solid-state packing, which, by restricting molecular mobility, significantly reduces the *E* → *Z* photoisomerization efficiency.^41^ Nevertheless, solid-state *E* ⇆ *Z* photoisomerization is highly fatigue resistant; indeed, after ten photocycles of alternating irradiation at 436 and 365 nm (equivalent to ≈6 h of high-intensity light exposure), the overall spectral variation remains ≤2% (Figure 4a inset and Figure S45). Notably, ^1^H NMR spectra recorded on powders before and after irradiation show no detectable changes, supporting the chemical stability of the compounds under the applied irradiation conditions (Figures S46). Hence, the minor change in optical density observed in thin film may arise from subtle morphological rearrangements (*vide infra*).

As shown in Figure 4b, films of *E-* **3a**Ph_3_ brought to PSS_436_ exhibit, in the dark at 25 °C, UV–vis spectral changes indicative of thermally activated *Z* → *E* back-isomerization, proving the metastability of the *Z* isomer also in the solid state. However, thermal relaxation is significantly slower in thin films compared to solution (*t*_1/2_ ≈ 20 min vs ≈ 1 min at 25 °C, respectively) and does not follow a first-order kinetic, suggesting the presence of distinct microenvironments that affect relaxation dynamics (Figure 4b inset).^68,69^

Detailed analysis by X-ray powder diffraction (XRPD), atomic force microscopy (AFM), and polarized optical microscopy (POM) of *E*-**3a**Ph_3_ thin films unveils that light irradiation not only drives *E* ⇆ *Z* interconversion but also induces a significant morphological reorganization of the material.

XRPD analysis shows that pristine spin-coated films of *E*-**3a**Ph_3_ (Figure 5a, trace I) display a main diffraction peak at 2θ ≈ 3.7° (*d* = 2.3 nm, 001 plane), nicely matching with the calculated powder pattern from single-crystal structure data (Figure 5a, trace IV). Scherrer analysis estimates an average crystalline domain size of 33–35 nm. Upon irradiation at 436 nm (up to 2 h) the intensity of the main diffraction peak progressively decreases (Figure 5a, trace II), indicative of a gradual amorphization of the film.

**Figure 5:**
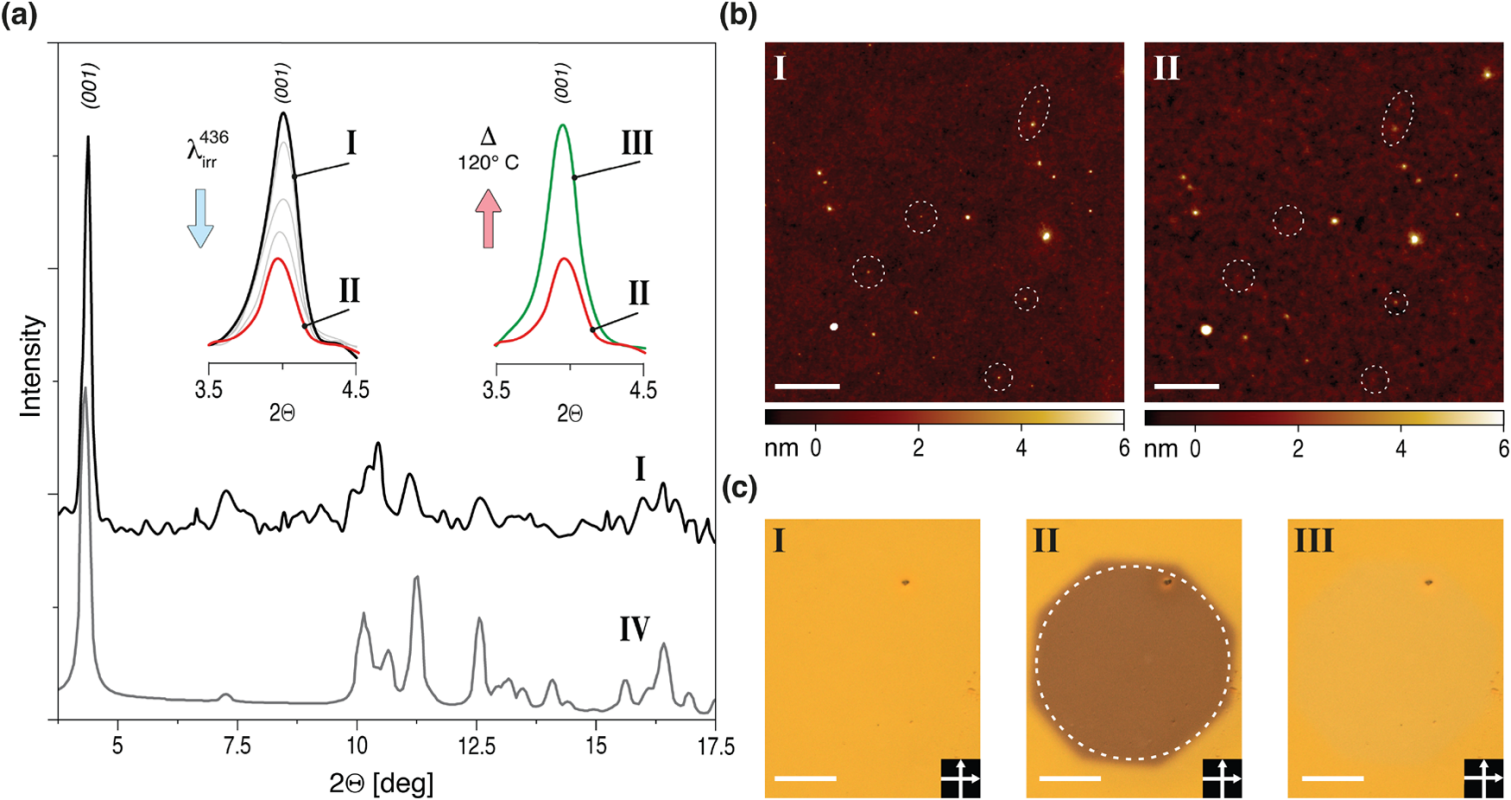
(a) XRPD patterns of a spin-coated film of *E*-**3a**Ph_3_ (thickness ≈ 150 nm, spin-coated from a 3 mg/mL solution at 1500 rpm), before (I), after irradiation at 436 nm (≈20 mW·cm^–2^) for 2 h (II), and after subsequent thermal annealing at 120 °C for 20 min (III). Patterns acquired at intermediate times are shown in light gray. The pattern calculated from single-crystal data of *E*-**3a**Ph_3_ is also shown (IV). (b) AFM morphology images showing the same area of a spin-coated film of *E*-**3a**Ph_3_ (thickness ≈ 60 nm) before (I), and after irradiation at 436 nm (≈20 mW·cm^–2^) for 2 h (II). The white dotted circles highlight some of the clusters that become less visible and eventually disappear upon irradiation. Scale bar: 1 μm. (c) Polarized optical micrographs (POM) of a spin-coated film (thickness ≈ 150 nm) of *E-* **3a**Ph_3_ before (I), after localized irradiation at 436 nm (dotted white circle) for 20 min (II), and after thermal annealing at 120 °C for 20 min (III). The white arrows represent the relative orientation of the polarizer and analyzer. Scale bar: 30 μm.

AFM analysis reveals that pristine spin-coated films of *E-* **3a**Ph_3_ exhibit a uniform morphology characterized by the presence of nanoclusters with lateral dimensions in the order of tens of nanometers, in agreement with the crystalline domain size estimated from XRPD (Figure 5b panel I). After irradiation at 436 nm for 2 h (Figure 5b panel II), two main effects are evident: (i) the film morphology becomes less uniform, a change quantitatively reflected in the evolution of the roughness (Table S8), and (ii) smaller nanoclusters progressively fade and eventually disappear, while most of the larger clusters broaden laterally and decrease in height (Figures S47 and S48). Overall, these features indicate that irradiation at 436 nm induces structural changes that reduce the degree of crystallinity of the film without significantly affecting the domain size, consistent with the observed decrease in XRPD peak intensity without appreciable peak broadening. Accordingly, POM images acquired under crossed polarizers show a localized loss of birefringence in regions irradiated at 436 nm, indicative of a transition to a less ordered, more isotropic phase (Figure 5c, compare panels I and II).

Notably, these morphological changes develop on a significantly slower time scale than the *E* ⇆ *Z* photoisomerization. Under continuous irradiation at either 365 or 436 nm (≈20 mW·cm^–2^), films of comparable thickness reach their respective PSS within ≈ 20 min, as determined by UV–vis spectroscopy. In contrast, XRPD and AFM reveal substantial amorphization only after more than 1 h of irradiation with light of the same intensity. Thus, structural reorganization of the films proceeds more slowly than molecular photoisomerization, most likely because the former is mediated by slow diffusive processes.^70,71^

Furthermore, although exhaustive irradiation at 365 nm of films brought to PSS_436_ restores an almost quantitative *E*-isomer composition, as revealed by UV–vis spectroscopy, the original degree of crystallinity is not recovered as shown by XRPD, AFM, and POM data. Nevertheless, crystallinity and birefringence are reinstated after thermal annealing at 120 °C for 20 min (Figure 5a trace III, and Figure 5c panel III). This indicates that the *E*-rich phase, obtained upon 365 nm irradiation, persists in a kinetically trapped amorphous state that requires thermal activation to recrystallize. This behavior is also evident in drop-cast films, where micron-sized crystallites lose both birefringence and euhedral morphology upon irradiation at 436 nm, and new crystalline domains with a distinct crystal habit form only upon thermal annealing (Figure S49). Notably, irradiation of *E-* **3a** films under identical conditions leaves both crystallinity and morphology unchanged, further supporting that the morphological modifications observed in *E-* **3a**Ph_3_ films are a direct consequence of *E* ⇆ *Z* photoisomerization and not of light-induced thermal effects (Figure S50).

### 2.4 Single Crystal Structure Analysis

To elucidate the impact of the bulky trityl substituent on molecular packing and its implication in the solid-state photochromic behavior of *E-* **3a**Ph_3_, high-quality crystals grown from chloroform were analyzed by X-ray diffraction (see SI, Table S9) and compared to those of the unsubstituted parent compound *E-* **3a**.

*E-* **3a**Ph_3_ crystallizes in the triclinic *P*–1 space group and presents columns with ordered stacks of thiophene backbones interleaved with columns of triphenyl branches (Figure 6a). In the asymmetric unit are present two symmetry-independent molecules, differing in the dihedral angles (1.0° and 21.1°) between the phenyl and dithienothiophene thienyl rings. These two conformers adopt an antiparallel orientation to reduce steric clashes between the trityl moieties, resulting in a staggered stacking motif between alternating pairs of the conformers with interplanar distances of ≈3.60 Å (Figure S51). Conversely, *E*-**3a** crystallizes in the monoclinic *P*2_1_ space group and exhibits nearly planar molecules (dihedral angle ≈ 3.1°) packed in cofacial slipped stacks with interplanar distances of ≈3.43 Å arranged in ordered columnar arrays with a dihedral angle of 81° between molecules (Figure 6b). The lower packing coefficient of *E-* **3a**Ph_3_ (0.698) compared to *E*-**3a** (0.726) proves that the rigid, sterically demanding trityl group disfavors dense packing, generating additional free volume and weaker intermolecular contacts.

**Figure 6:**
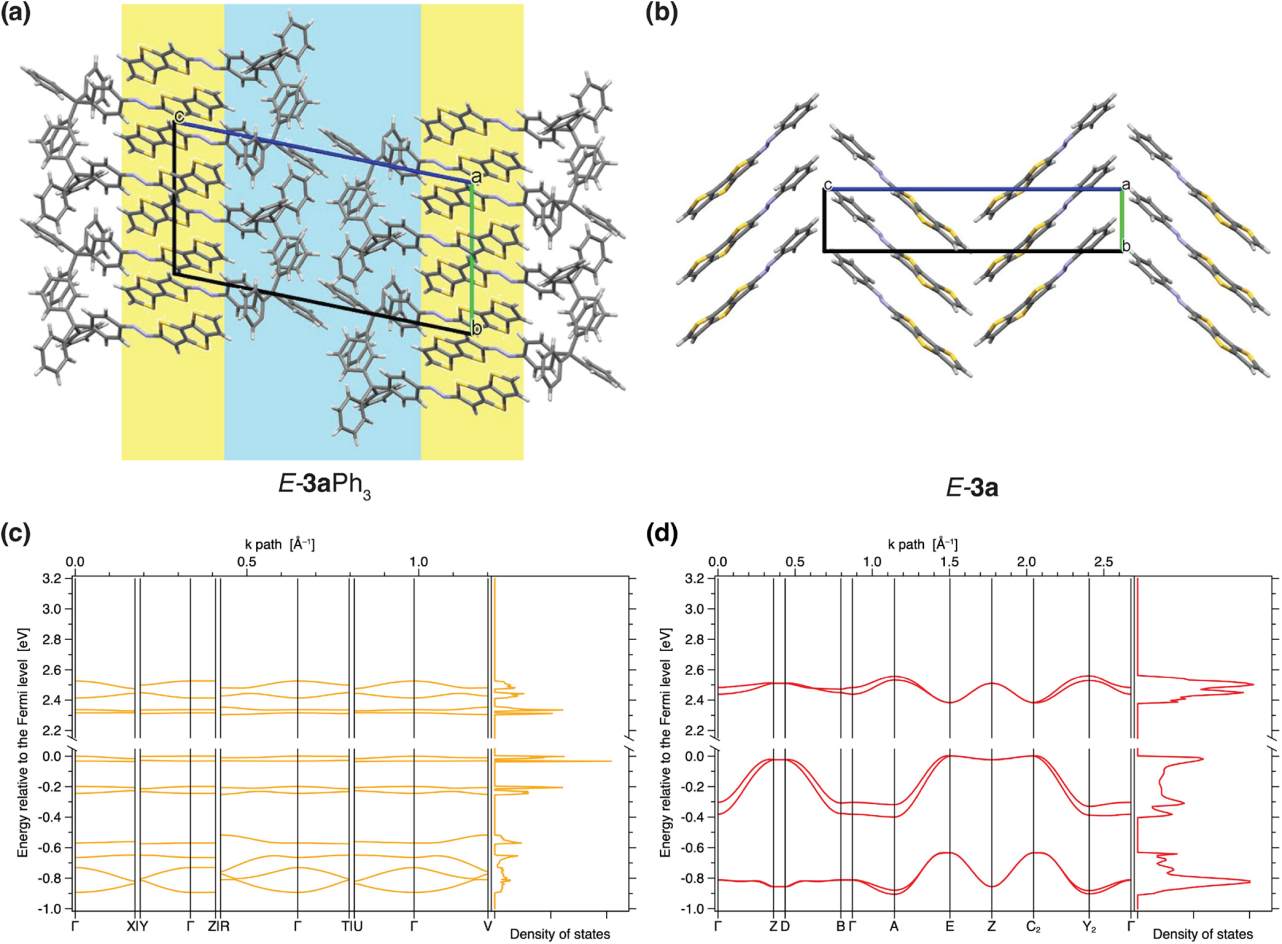
Projection down the *a*-axis of single crystal structures for (a) *E-* **3a**Ph_3_ and (b) *E-* **3a**. In (a), the different colored areas highlight the distinct portions of the crystal volume where the flat thiophene backbones or the bulky triphenyl branches are settled. Band structure and density-of-states plots of (c) *E-* **3a**Ph_3_ and (d) *E-* **3a** crystals calculated at the HSE06/POB-TZVP-rev2 level of theory on geometries from single-crystal X-ray diffraction data.

Periodic DFT calculations (HSE06/POB-TZVP-rev2) show that the *E-* **3a**Ph_3_ crystal, despite its looser packing and larger unit cell, displays the same topology of the crystal frontier orbitals as *E*-**3a** (Figures S40 and S41). In both materials, the HOCO (highest occupied crystal orbital) and LUCO (lowest unoccupied crystal orbital) are delocalized along the π-conjugated backbones, confirming that the azothiophene core governs band structure, whereas the trityl groups contribute negligibly to the band-edge states. Interestingly, in *E-* **3a**Ph_3_, the presence of four molecules per unit cell leads to two pairs of strongly interacting molecules, resulting in a partial splitting of the HOCO/LUCO orbitals and preserving intermolecular electronic coupling despite the increased intermolecular distance imposed by the trityl groups (Figure 6c). As shown in Figure 6d, although *E*-**3a** exhibits larger band dispersion along the stacking direction (*b* axis, Figure S42), indicative of stronger π–π orbital overlap, *E-* **3a**Ph_3_ still maintains an appreciable coupling with only a modest reduction in bandwidth. The computed crystal band gaps are 2.30 eV for *E-* **3a**Ph_3_ and 2.38 eV for *E*-**3a**, indicating that the introduction of the trityl moiety, despite decreasing packing density, leads to a slight narrowing of the HOCO-LUCO gap consistent with the observed trend of the HOMO–LUMO gap at the molecular level.

### 2.5 Photomodulation of Conductivity

Photomodulation of electrical conduction was investigated in planar two-contact devices, fabricated by spin-coating chloroform solutions of *E-* **3a**Ph_3_ or *E*-**3a** (as a nonphotoisomerizable control), onto an Ag interdigitated pattern spaced by 100 μm (Figure S52). The current was continuously measured at a fixed low bias of 0.1 V.

To probe the impact of *E* ⇆ *Z* photoisomerization, devices were alternately irradiated with 436 and 365 nm light (20 min each). The current was recorded in the dark, 20 min after each irradiation step, to rule out photothermal and photogeneration contributions.

As shown in Figures 7a and S53, irradiation at 436 nm of *E-* **3a**Ph_3_ based devices (PSS_436_) induces a small but reproducible decrease in the current (red circles) compared to the pristine state (black circle), whereas subsequent irradiation at 365 nm (PSS_365_) restores the current to its initial value (blue circles). The on/off current ratio, though moderate, remains reproducible up to six cycles of alternating visible and UV light irradiation, although a gradual decrease in the current modulation amplitude is observed over time. In contrast, *E*-**3a** based devices, under identical irradiation conditions, do not show appreciable current photomodulation (Figure 7a, bottom), indicating that the response observed in **3a**Ph_3_ based devices originates from *E* ⇆ *Z* photoisomerization, rather than from nonspecific photothermal effects.

**Figure 7:**
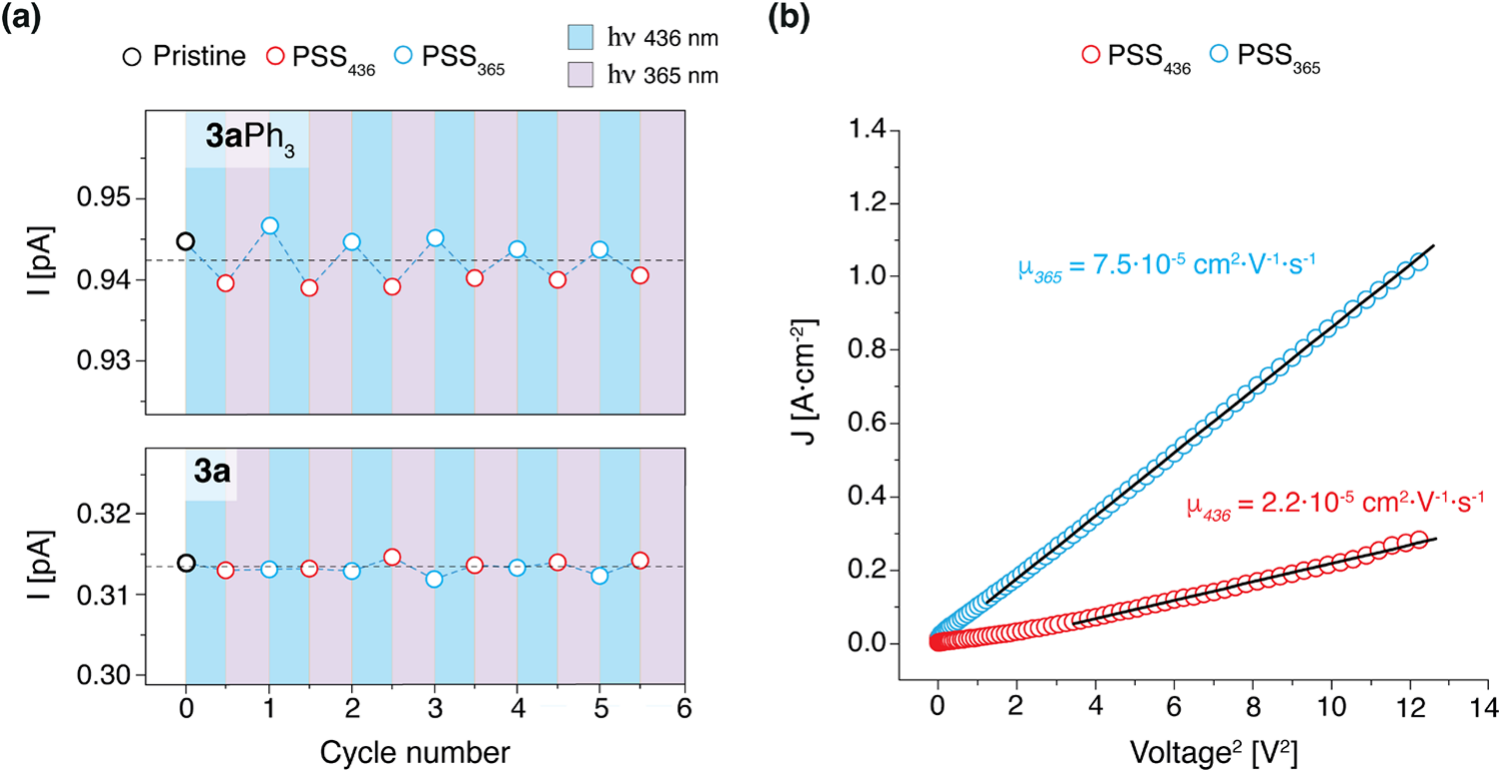
(a) Current response, under a fixed 0.1 V bias, of planar devices (*N* ≥ 3 devices) with Ag interdigitated electrodes (channel width: 100 μm, channel length: 5 mm) fabricated from *E*-**3a**Ph_3_ (top) and *E*-**3a** (bottom) spin-coated films (thickness ≈ 60 nm). Current measured in the pristine state (black circles) and upon repeated cycles of alternating irradiation for 20 min at 436 nm (PSS_436_; red circles) and 365 nm (PSS_365_; blue circles). The horizontal dotted line serves as a guide for the eye. (b) Variation of current density with the square of voltage for an electron-only ITO/ZnO/*E*-**3a**Ph_3_/Ca/Al device upon irradiation at 365 nm (PSS_365_; blue circles) or at 436 nm (PSS_436_; red circles) for 20 min; black lines indicate the linear fit to the data. *E*-**3a**Ph_3_ layer thickness: 64 nm_._ Light intensity at 365 and 436 nm: ≈20 mW·cm^–2^.

Given the large mismatch between the Ag work function (≈4.3–4.7 eV) and the frontier energy levels of **3a**Ph3 (Figure S23), contact injection barriers are expected to contribute significantly to the overall resistance. Accordingly, the mechanism underlying the reversible photomodulation of the current can be attributed to differences in energy-level alignment between the *E* and *Z* isomers at the Ag/organic interface, whereas the progressive current attenuation upon cycling is consistent with cumulative, irreversible film amorphization that disrupts percolation pathways, as evidenced by XRPD, AFM, and POM analyses on thin films.

To probe the intrinsic charge-transport properties of *E*-**3a**Ph_3_ in its *E*- and *Z*-enriched PSSs (Figure 7a, top) the bulk mobility (μ) was investigated by using the space-charge-limited-current (SCLC) method, applied to one carrier-only devices.^72^ Because the HOMO of **3a**Ph_3_ is very deep (≈6.05 eV), efficient hole injection is not readily achieved with standard high work-function electrodes, whereas electron injection can be favored using Ca (≈2.8–2.9 eV) contacts owing to their favorable alignment with the LUMO (≈3.58 eV). Accordingly, electron-only devices with the architecture ITO/ZnO/*E*-**3a**Ph_3_/Ca/Al were fabricated: Ca provides a low injection barrier for electrons, while the ZnO interlayer suppresses hole injection, ensuring single-carrier selectivity. Furthermore, the vertical device geometry reduces the active layer transport distance in the order of tens of nm instead of the hundreds of micrometers of planar devices, reducing the dependence on the morphological variation of the film structure. The electrical characterization was performed in the dark, under argon atmosphere. As shown in Figure 7b, the current-density *J* exhibits a very good quadratic trend with the voltage, as expected for a space-charge-limited current, both upon irradiation at 365 nm and at 436 nm. Mobility values were derived by applying the Mott–Gurney law for the SCLC regime, with the mobility independent of the electric field (see SI for details). An electron mobility of 7.5 × 10^–5^ cm^2^·V^–1^·s^–1^ was obtained for the device brought to PSS_365_ (upon irradiation at 365 nm for 20 min; intensity ≈ 20 mW·cm^–2^), whereas at PSS_436_ (upon irradiation at 436 nm for 20 min; intensity ≈ 20 mW·cm^–2^) the value decreases to 2.2 × 10^–5^ cm^2^·V^–1^·s^–1^. The variation of mobility observed for the *Z*- and *E*-rich PSSs–obtained with short irradiation times that minimize morphological changes–demonstrates that the isomeric composition of the film affects its charge transport properties. The reduced mobility of the *Z*-rich PSS_436_ can be rationalized by the loss of planarity and decreased π-overlap along the thiophene backbone that reduces charge hopping and intermolecular electronic coupling.

## 3 Conclusions

Two novel classes of photochromic π-extended azo compounds–arylazo α-oligothiophenes and oligothienoacenes–have been synthesized and systematically investigated for their photochromic behavior in both solution and solid state.

In solution, these compounds exhibit photochromic characteristics consistent with well-established trends: extension of π-conjugation leads to bathochromic shifts in absorption and reduced persistence of the *Z* isomer. This latter effect can be mitigated by the introduction of fluorine substituents, which significantly increases the half-life of the *Z*-isomer by enhancing stabilizing S­(n)···π interactions in the ground state.

A particularly noteworthy feature of both series of compounds is their wavelength-dependent PSS composition, which favors formation of the *Z* isomer upon visible-light irradiation, whereas an *E*-isomer rich composition is obtained upon UV irradiation, thereby exhibiting a complementary behavior compared to most arylazo photochromic compounds. This peculiar feature may open interesting avenues for the design of multichromophoric systems with complementary photoswitching behavior.

As expected, strong intermolecular interactions between the azo and oligothiophene units suppress photochromism in the solid state for all investigated compounds. This limitation was successfully overcome through crystal-packing engineering. In particular, the introduction of a bulky trityl group in *E*-**3a**Ph_3_ proved effective in enabling photochromism in the solid state by disrupting tight packing, driving a transition from the compact arrangement observed in the *E*-**3a** analogue to a slack π-stacked architecture. Differently, in *E*-**3**Ph_3_ the bulky trityl group, while it enhances the processability of the material, is not sufficient to promote solid state photochromism mainly due to the stronger packing of the more π electron rich α-linked oligothiophene scaffold. These findings highlight that efficient solid-state photoswitching requires a fine structural balance between intermolecular interactions and lattice arrangement: while *E*-**3a**Ph_3_ fulfils this requirement, further functionalization strategies are needed to extend this behavior to α-linked oligothiophene systems.

Thin films of *E*-**3a**Ph_3_ exhibit robust solid-state photochromism coupled to a progressive light-induced crystalline-to-amorphous morphological transformation directly evidenced by XRPD, POM, and AFM analyses. Importantly, *E* ⇆ *Z* photoisomerization proceeds on a faster time scale than amorphization, thereby rendering the two processes temporally disentangled.

The molecular structure of *E*-**3a**Ph_3_–combining solid-state photochromism of the azo unit with semiconductive properties of the oligothiophene backbone–enabled the fabrication of simple optoelectronic devices that exhibit light-controlled modulation of electrical conduction. In planar two-contact devices, the current modulation upon alternating irradiation is consistent with isomer-dependent energetics at the Ag/organic interface, where contact injection barriers contribute significantly to the overall resistance. The progressive attenuation of the modulation upon repeated cycling is, in turn, consistent with cumulative irradiation-induced amorphization of the active layer, indicating that the electrical response becomes increasingly influenced by the film’s mesoscale morphological evolution. In contrast, in electron-only SCLC devices—designed to minimize contact limitations and morphological contributions—the extracted bulk electron mobility decreases by ∼4-fold between the *E*- and *Z-*enriched PSSs, revealing that isomeric composition intrinsically affects charge transport. A direct comparison of these absolute mobility values with literature precedents is not straightforward, given the scarcity of closely related molecular systems, and because reported mobilities depend heavily on device architecture, electrode configuration, film morphology, contact barriers, and the specific extraction methods employed. Hence, the observed 4-fold mobility modulation should be regarded primarily as a mechanistically well-supported proof of principle, rather than as an optimized device figure of merit.

Overall, these results establish arylazo oligothiophenes as a peculiar material platform where intrinsic molecular photochemistry and light-driven mesoscale structural dynamics can be probed and correlated to charge transport. On a broader perspective, this study prompts a re-evaluation of π-extended azo compounds as viable photochromic moieties, highlighting their limitations and strengths, as well as their potential for the development of multifunctional photoresponsive materials and next-generation optoelectronic architectures.

## Supplementary Material



## Notes

Material synthesis; additional structural and spectroscopic characterization; theoretical calculation details; AFM and optical microscope images; single crystal structures; device characterization (PDF)
