# Supplementary material for: Coupling Photochromism and Charge Transport in π‑Extended Arylazo Oligothiophenes and Oligothienoacenes
Source: J Am Chem Soc. 2026 Jul 17;148(29):31020–31. doi: 10.1021/jacs.6c06319 (PMC13426255; doi:10.1021/jacs.6c06319)
Supplement: Supplementary file 1 [file ja6c06319_si_001.pdf]

SUPPLEMENTARY INFORMATION

Coupling Photochromism and Charge Transport in π-Extended Arylazo Oligothiophenes and Oligothienoacenes

Chiara Taticchi, Federico Nicoli, Mattia Zangoli, Filippo Monti, Eugenio Lunedei, Alessandro Turci, Massimiliano Curcio, Pierluigi Mondelli, Mario Caironi, Magda Monari, Massimo Gazzano, Andrea Candini, Alberto Zanelli, Francesca Tinti, Nadia Camaioni, Alberto Credi, Massimo Baroncini,* Francesca Di Maria*

I.	Experimental details	p. S2-S6
II.	Materials and methods	p. S7
III.	Synthetic procedures	p. S8-S12
IV.	NMR characterization	p. S13-S28
V.	Theoretical calculations	p. S29-S42
VI.	Cyclic voltammetry measurements (CVs)	p. S43
VII.	PhotoNMR characterization of **2a**F_4 and **3a**Ph_3	p. S44-S46
VIII.	UV-Vis photochemical characterization in solution	p. S47-S57
IX.	Periodic DFT calculations on *E*-**3a** and *E*-**3a**Ph_3 crystals	p. S58-S60
X.	Solid-state UV-Vis characterization	p. S61-S63
XI.	AFM images and data analysis	p. S64-S65
XII.	Optical microscopy	p. S66-S67
XIII.	Single crystal structures determination	p. S68-S69
XIV.	Devices fabrication and characterization	p. S70
XV.	References	p. S71

I. Experimental details

NMR and PhotoNMR Spectroscopy. NMR spectra were recorded on an Agilent DD2 spectrometer operating at 500 MHz. Chemical shifts are quoted in parts per million (ppm) relative to tetramethylsilane using the residual solvent peak as a reference standard and all coupling constants (J) are expressed in Hertz (Hz). Photochemical NMR experiments (PhotoNMR) were performed in air-equilibrated CD_2Cl_2 solutions at 298 K. Irradiation was performed directly inside NMR tubes in the spectrometer probehead using a Prizmatix FC-LED-365A or FC-LED-445HR illuminator equipped with an FCA-SMA adaptor for optical fibers. The desired irradiation wavelength of 365 or 450 nm was selected using appropriate hard coated OD 4.0 band-pass filter ($\Delta\lambda$ = ±10nm). Quartz optical fiber (core 1000 µm, 5 m) equipped with a SMA connector on one end was purchased from Thorlabs. The other end of the optical fiber was scraped to remove the protective coatings, exposing the quartz core, and inserted into the solution within a quartz coaxial tube (New ERA).

UV-Vis spectroscopy. Absorption spectra were recorded on Lambda750 (Perkin Elmer) double beam spectrophotometers. One cm path length quartz cuvettes (Hellma) were employed. In the case of solutions of high absorption, 1 mm or 2 mm path length cuvettes were used. Quartz slides (Hellma) were employed for the preparation of spin coated thin films.

Spin-coated thin film preparation. For studies of thin films, concentrated $CHCl_3$ solutions of the compounds (ranging from 7 mM to 20 mM) were deposited on quartz or glass slides by spin coating at room temperature. The rotation of the instrument plate was set to ~1500 rpm (quartz) or 800 rpm (glass) for 1 min. Optical micrographs were taken using a Nikon Eclipse 80i polarizing optical microscope.

UV-Vis irradiation experiments in solution. Irradiation experiments were performed on thoroughly stirred air-equilibrated solutions. Absorption spectra were recorded with a diode array spectrophotometer equipped with an optical fiber probe (Avantes StarLine AvaSpec-ULS2048CL-EVO-RS) at high rates under continuous irradiation. Irradiation was performed with a medium pressure Hg lamp (200 W), the wavelength of irradiation was selected using an appropriate interference filter (10 nm band pass) and the intensity of the incident light was regulated with a pinhole of opportune diameter. The incident photon flux was measured using the ferrioxalate actinometer, according to the procedure reported for its "microversion".[1] The absorption spectra of the *Z* isomers were obtained mathematically, according to the method reported by Fischer.[2] The photoisomerization quantum yields ($\Phi_{E\to Z}$, $\Phi_{Z\to E}$) were determined by global fitting of the time-dependent absorbance changes, monitored both at the irradiation wavelength and at the wavelength showing the largest spectral variation (corresponding to the π–π* absorption maximum), using the photokinetic equation reported below[3]:

$$\frac{d[E]}{dt} = -\frac{d[Z]}{dt} = -\frac{\Phi_{E\to Z}\cdot q_0\cdot \varepsilon_{E,\lambda_i}\cdot [E]\cdot f}{V\cdot\left(\varepsilon_{E,\lambda_i}\cdot[E]+\varepsilon_{Z,\lambda_i}\cdot[Z]\right)} + \frac{\Phi_{Z\to E}\cdot q_0\cdot \varepsilon_{Z,\lambda_i}\cdot [Z]\cdot f}{V\cdot\left(\varepsilon_{E,\lambda_i}\cdot[E]+\varepsilon_{Z,\lambda_i}\cdot[Z]\right)} + k_\Delta\cdot[Z]$$

where q_0 is the incident photon flux, V is the volume of the irradiated solution, $\varepsilon_{E,\lambda i}$ and $\varepsilon_{Z,\lambda i}$ are the molar absorption coefficients of the pure *E* and *Z* isomers at the selected irradiation wavelength (λ_i), and k_Δ is the thermal back-isomerization rate constant. The term f represents the fraction of incident light absorbed by the system:

$$f = 1 - 10^{-A_{\lambda_i}}$$

where:

$$A_{\lambda_i} = \varepsilon_{E,\lambda_i}\cdot[E] + \varepsilon_{Z,\lambda_i}\cdot[E]$$

For simplicity, the optical path length is assumed to be 1 cm and omitted from the formalism. The photokinetic equation accounts simultaneously for three competing processes: forward photoconversion (*E*→*Z*, first term), reverse photoconversion (*Z*→*E*, second term), and thermal relaxation (third term). This differential equation cannot be solved analytically and instead requires numerical integration to accurately extract photophysical parameters (quantum yields values). In our case, Berkeley Madonna software was employed to numerically fit the time-dependent absorbance changes (represented as a red line in the graphs) and determine the quantum yields. However, in the case of extremely fast thermal processes ($t_{½}$ < 100 sec), the error associated to the determination of the back photoisomerization quantum yield ($\Phi_{Z\rightarrow E}$) is higher than 30%, primarily due to the increased difficulty in accurately defining the pure *Z*-isomers absorption spectra.[4] Thermal back-isomerization kinetics were investigated monitoring the absorbance changes over time in the dark at 298 K. The obtained data were fitted according to a first-order model.

UV-Vis irradiation experiments in thin films. Irradiation experiments were performed on spin coated thin film deposited on quartz or glass slides with a Hg lamp (200 W). The desired wavelength of irradiation was selected using an appropriate interference filter (10 nm band pass) and the intensity of the incident light was regulated with a pinhole of opportune diameter. Irradiation experiments in the optical microscope were performed with an epifluorescence optical setup with a 100 W Hg lamp through a Nikon C-FL BV Wide Blu filter (420 - 490 nm).

Computational Methods. All non-periodic calculations were performed using ORCA 6.0.1[3] and the conductor-like polarizable continuum model (CPCM) was adopted to consider dichloromethane (DCM) solvation effects.[4] The *E* and *Z* isomers of all the investigated molecules were fully optimized, without symmetry constraints, using the range-separated ωB97M-D4 functional,[5] in combination with the def2-TZVP triple-ζ valence basis set.[6] All calculations employed the TightSCF convergence criterion, and harmonic vibrational frequencies were computed to confirm the nature of each stationary point found by the geometry optimization algorithms.
To explore the excited-state scenario of these molecules at the Franck–Condon region and to model their absorption spectra, the so-obtained ground-state minimum-energy geometries were taken for subsequent time-dependent density functional theory (TD-DFT) calculations,[7] within the Tamm–Dancoff approximation (TDA),[8] using the same level of theory already adopted for the geometry optimizations and the RIJCOSX procedure to speed up the calculations.[9] To obtain a higher-level description of such excited states and to improve the accuracy of the computed $S_0 \rightarrow S_n$ vertical excitation energies, similarity-transformed equation-of-motion domain-based local pair natural orbital coupled-cluster singles and doubles calculations (STEOM-DLPNO-CCSD)[10] were also performed with the def2-TZVP(-f) orbital basis set,[11]using the def2-TZVP/C and def2/J auxiliary basis sets for an efficient evaluation of the electron–electron correlation and Hartree–Fock exchange contributions, respectively.[12] Dichloromethane solvation effects were considered also in this case within the CPCM approach.[13] The nature of the lowest lying electronic excitations was characterized using the natural transition orbital (NTO) formalism, which affords a compact representation of each excitation in terms of its leading hole-electron pair.[14]
For a representative subset of molecules (*i.e.*, **1**, **2**, **2a** and **2a**F_4), the thermal $Z \rightarrow E$ isomerization process was investigated to identify the most efficient mechanism (*e.g.*, inversion or torsion around the azo N=N bond). It is known that DFT can properly identify transition states (TSs) along the inversion pathways, not only in azobenzene derivatives, but also in azothiophene counterparts[15] and,

indeed, relaxed surface scans (with steps of 5°) were successfully performed at the ωB97M-D4/def2-TZVP considering the linearization of both the phenyl (C_{ph}–N=N) or thiophene (N=N–C_{thio}) angles, and the corresponding TSs were fully found and optimized. On the contrary, single-reference DFT methods fail to properly identify torsional transition states in azobenzene-like systems[16] and relaxed scans of the C–N=N–C dihedral angle were effective just at angles far from 90°, otherwise the relaxation would converge to linear TSs (inversion pathways); accordingly, in such critical region, constrained relaxed scans were performed at frozen C_{ph}–N=N and N=N–C_{thio} angles to avoid inversion. Since DFT is known to yield a wrong potential-energy profile along torsion (due to the single-reference DFT limit and multi-reference torsional-TS nature) and a too low barrier along inversion, we adopted a combined DFT-multireference approach inspired by a recent paper of Aleotti *at al.*,[17] using single-point CASSCF/NEVPT2 calculations on the top of DFT-optimized geometries from the previously mentioned scans. Such single-point CASSCF calculations were carried out as implemented in ORCA 6.0.1,[18] adopting the def2-SVP basis set[11] in combination with the auxiliary def2-SVP/C basis for speeding up the evaluation of the electron–electron Coulomb term (using the resolution-of-identity (RI) approximation).[19] For all the calculations, an active space of 14 electrons in 12 orbitals was selected (see **Figure S25** for a complete active orbital description), and 2 singlet and 1 triplet states were considered in a state-averaged approach with all equal weights. Dynamic correlation beyond CASSCF level was included by second-order N-electron valence state perturbation theory (strongly contracted NEVPT2).[20]
To theoretically model the ^{1}H NMR spectral changes associated with the Z–E isomerization, NMR shielding tensors were computed at the PBE0/def2-TZVPP in dichloromethane (CPCM) for compound **2a**F_4, selected as representative example of the series. The calculations were performed on the fully optimized ground-state geometries obtained at the ωB97M-D4/def2-TZVP level (see above). All NMR calculations employed gauge-including atomic orbitals (GIAO) as implemented in ORCA, together with the TightSCF convergence criterion. Absolute isotropic shieldings (σ) were converted into chemical shifts (δ, ppm) using tetramethylsilane (TMS) as reference, according to the formula: $\delta = \sigma_{TMS} - \sigma_{sample}$. In addition, the anisotropy and asymmetry of the chemical shielding tensors were analyzed from the diagonalized total shielding matrices reported by ORCA, yielding the three principal components (σ_{11}, σ_{22}, σ_{33}), which were used to evaluate the chemical shielding anisotropy (Δσ) and asymmetry (η), following the Haeberlen convention:

$$|\sigma_{33} - \sigma_{iso}| \geq |\sigma_{11} - \sigma_{iso}| \geq |\sigma_{22} - \sigma_{iso}|$$

$$\sigma_{iso} = \frac{\sigma_{11} + \sigma_{22} + \sigma_{33}}{3}$$

$$\Delta\sigma = \sigma_{33} - \sigma_{iso} \quad \text{and} \quad \eta = \frac{\sigma_{22} - \sigma_{11}}{\Delta\sigma}$$

To evaluate the influence of the bulky trityl group on the electronic properties of **3a** and **3a**Ph_3, not only at the molecular level but also in the solid-state, periodic DFT calculations were performed with CRYSTAL23[21] using the range-separated HSE06 hybrid functional[22] and the POB-TZVP-REV2 Gaussian basis set.[23] The crystal cells and atomic coordinates were taken directly from the experimental CIF files obtained by single-crystal X-ray diffraction, with only the C–H bond lengths adjusted to 1.090 Å. Single-point calculations were carried out on these fixed geometries employing a Γ-centered Pack–Monkhorst grid with a k-point spacing of 0.150 $Å^{-1}$ along the reciprocal lattice

vectors, which was further refined at 0.075 Å^{-1} for the density-of-states (DOS) evaluation. Band structures were subsequently computed along the conventional high-symmetry paths of the Brillouin zone, as defined by the space group of each crystal.
Visualizations of molecular orbitals and natural transition orbitals (NTOs) were generated using Visual Molecular Dynamics (VMD), version 1.9.4a57.[24] In the case of periodic systems, crystalline orbitals were obtained with Jmol.[25] Non-covalent interaction (NCI) analyses and graphs were performed using Multiwfn, version 3.8.[26]

Cyclic Voltammetry (CV): CVs of all compounds were performed at room temperature on 0.1 mmol L^{-1} solutions purged with Ar, with an AMEL 5000 Electrochemical System on Pt working electrodes (1 mm diameter) in a three compartment glass cell with a Pt wire spiral and an aqueous saturated calomel electrode (SCE) as the auxiliary and reference electrodes, respectively. The supporting electrolyte was CH_2Cl_2 (Sigma-Aldrich for HPLC >66.5%, distilled over P_2O_5 and stored under Ar) in 0.1 mol L^{-1} $(C_4H_9)_4NClO_4$, (Fluka, electrochemical grade) where the standard potential of the ferrocene/ferrocenium (FC^+/FC) couple is E°[FC^+/FC] = 0.475 V vs. SCE.

XRPD thin films analysis. X-ray powder diffraction (XRPD) patterns of film samples were recorded by using a PANalytical X'PertPro diffractometer equipped with a copper target (λ= 0.15418 nm) and a fast Solid-state X'Celerator detector. The measured films were ≈ 150 nm thick, to increase the signal-to-noise ratio. Data were recorded in the 3-30° 2 theta interval, by collecting for 40 sec at each step (0.05°). Irradiation of the film was performed directly *in situ* mounting the lamp inside the diffractometer at a distance of 4 cm from film surface. A CoolLED pE-300 light source, fed into a UV/Vis LLG (Liquid Light Guide) and coupled with a collimation optic was used. The intensity was set to maximum (100%, ~20 mW/cm^2 for both 365 nm and 436 nm wavelengths). The *001* peak was taken as a marker of the crystal phase presence. A repetitive data collection was performed during irradiation.

AFM microscopy. Topographic atomic force microscopy (AFM) images have been acquired employing a Multimode 8 (Bruker) microscope operated in air, employing Pt/Ir-coated cantilever-doped silicon tips (SCM-PIT-V2, Bruker) with mechanical constant k = 3 N/m and oscillating frequency f_0 ≈ 75 kHz. Measurements were performed on spin coated thin film deposited on silicon slides. Irradiation was performed with LED light sources at 365 nm and 436 nm. The desired wavelength of irradiation was selected using an appropriate interference filter (10 nm band pass). XRD single crystal analysis. The X-ray intensity data for *E*-3a and *E*-3aPh_3 were collected on a Bruker APEX-II CCD diffractometer using Mo−Kα or Cu-Kα radiation (for *E*-3aPh_3). All data were processed using the Bruker suite of programs[27,28,29] and the structures were solved by direct methods and refined with the SHELX program suite.[30,31] All non-hydrogen atoms were assigned anisotropic displacement parameters. Most of the hydrogen atoms were located in the Fourier map, placed in idealized positions and included as riding with constrained isotropic displacement parameters (C—H = 1.09 Å) for the aromatic protons and refined as riding with U_{iso}(H) = 1.2U_{eq}(C)). In *E*-3aPh_3 two independent molecules are present in the asymmetric unit. Molecular graphics were generated using the program Mercury.[32] Table S9 reports crystal data and refinement parameters for *E*-3a and *E*-3aPh_3.

Electrical characterization. Bulk conductivity photomodulation was investigated for *E*-3aPh_3 and, for comparison, *E*-3a using devices fabricated by spin-coating chloroform solutions onto interdigitated vacuum evaporated silver electrodes (300 nm thick, 100 μm channel spacing). The film thickness, assessed by profilometry, was maintained in the range of ~ 60 nm. Current–voltage (*I–V*) characteristics were recorded under high vacuum in the dark at 296 K (see page S65 for further details).

To probe the effect of photoisomerization on material's conductivity, the devices were subjected to alternating cycles of irradiation at 'VIS' (436 nm light) and 'UV' (365 nm light) for 20 min each. In order to exclude any thermal effect or photogeneration artifact, conductivity measurements were performed under vacuum (base pressure P ~ $5*10^{-5}$ mbar), in the dark, 10 min after cessation of irradiation under a chosen fixed voltage bias.
To irradiate the film, a CoolLED pE-300 source, fed into a UV/Vis LLG (Liquid Light Guide) and coupled with a collimation optic was used. The intensity was set to maximum (100%, ~20 mW/cm^2 for both sources). The illuminated area was carefully selected to cover uniquely the central part of the two-faced electrodes (separation 100μm, *see* **Figure S46** for the device structure). The external connections were not illuminated. The experimental setup was programmatically driven by a computer, which controlled the irradiation intensity, its peak wavelength (UV / VIS) and the on/off timing, while the flowing current I_D was synchronously measured by a Keithley 236 (SMU, Source Measure Unit) at a constant bias of V_{fix} = 0.1 V, integration 20 ms, averaged on 16 readings, measurement resolution 5 digits, limit current resolution +/- 10fA. The raw data points were successively adjacent averaged over 91 points.
The measurement was carried on as follows: after a suitable stabilization time (of the order of 30 min), a sequence of: [VIS (436 nm) irradiation (20 min), wait time in the dark (10 min), Current measuring (10 min), UV (365 nm) irradiation (20 min), wait time in the dark (10 min), Current measuring (10 min)] was repeated for N cycles (N = 6 or more). **Figure S47** shows the cycle-dependent electrical current flowing across a *E*-**3a**Ph_3 film (where photoisomerization is expected to take place) and, for comparison, the cycle-dependent electrical current flowing across a "control" *E*-**3a** film (where no photoisomerization effect is expected).
Electron-only devices were prepared in the sandwiched structure ITO/ZnO/**3a**Ph_3/Ca/Al, where ITO is indium-tin-oxide. ITO-coated glass substrates were first cleaned in water, then ultrasonicated in acetone and isopropyl alcohol for 15 min each. The substrates underwent a 20 min UV-Ozone treatment before the deposition of the ZnO precursor solution, which was spin-coated at 600 rpm for 160 seconds plus 5 seconds at 1000 rpm. A thermal annealing of 5 min at 140 °C completed the hydrolysis of the precursors to form a sintered film of transparent ZnO. The ZnO-coated substrates were then transferred into an Ar-filled glove-box to complete the device structure. The **3a**Ph_3 film (64 nm) was deposited by spin-coating (300 rpm) from a thiophene solution (10 g/L). The Ca layer (70 nm) and the Al top electrode (80 nm) were thermally evaporated at a base pressure of $4x10^{-6}$mbar through a shadow mask defining a device active area of 8 mm^2.
The electrical characterization of the devices was carried out in the dark, at room temperature in an Ar-filled glove-box, by using a Keithley 2400 source-measure unit.

The values of the bulk electron mobility were extracted from the current-density *J versus* voltage (*J-V*) curves fitted according to the Mott-Gurney law:

$$J = \frac{9}{8}\varepsilon\varepsilon_0\mu\frac{V^2}{d^3}$$

Where μ is the mobility, ε and ε_0 are the relative dielectric constant (assumed as 3) and the permittivity in vacuum, respectively, and d is the thickness.[33]

II. Materials and methods

TLC analysis was performed on 0.2-mm thick silica gel 60 F_{254} (Sigma). Preparative column chromatography was carried out on glass columns charged with silica gel 60 (particle sizes 0.040-0.063 mm, Sigma). Thiophene, 2,2'-bithiophene, 2,2':5',2''-terthiophene, thieno[3,2-b]thiophene and dithieno[3,2-b:2',3'-d]thiophene were purchased from Fluorochem; *N*-bromosuccinimide, *n*-butyllithium 2.5 M solution in hexane, $NaHCO_3$ and [1,1′-Bis(diphenylphosphino)ferrocene] dichloropalladium(II) Pd(dppf)Cl_2 were purchased from Sigma-Aldrich Co. Thieno[2',3':4,5]thieno[3,2-b]thieno[2,3-d]thiophene,[34] 4-(4,4,5,5-tetramethyl-1,3,2-dioxaborolan-2-yl)benzo[c][1,2,5]thiadiazole,[35] 3,3'-bis(methylthio)-2,2'-bithiophene,[36] dithieno[3,2-b:2',3'-d]thiophene-4,4'-dioxide[37] were synthesized according to literature procedures. All reagents and solvents were used as received. Organic solvents were dried by standard procedures.

Microwave experiments were carried out in a CEM Discover SP-Microwave Synthesizer reactor in a closed vessel (230W, fixed temperature at 80°C, air, high stirring rate). Reactions with ultrasound were performed in a FALC LBS1 50KHz Ultrasonic bath at room temperature. Mass spectra were collected on a Thermo Scientific TRACE 1300 gas chromatograph.

III. Synthetic procedures

Synthesis of diazonium salts

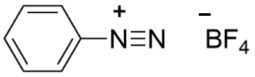

Benzenediazonium tetrafluoroborate (1d): **1d** was synthesized according to Ref. 40. **EI-MS**: *m/z* 192 (M^+). **^{1}H NMR** (500 MHz, $(CD_3)_2CO$, 298 K): δ (ppm) 8.82 (d, *J* = 8 Hz, 2H, *a*), 8.38 (t, *J* = 8 Hz, 1H, *c*), 8.08 (dd, *J* = 7,6 Hz, *J* = 1,5 Hz, 2H, *b*). **^{13}C{^{1}H} NMR** (126 MHz, $(CD_3)_2CO$, 298 K): δ (ppm) 141.43, 132.80, 131.60, 115.67. **^{19}F{^{13}C} NMR** (470 MHz, $(CD_3)_2CO$, 298 K): δ (ppm) -150.9 (s, 4F).

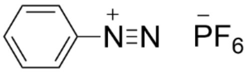

Benzenediazonium hexafluorophosphate (2d): **2d** was synthesized according to Ref. 40. **EI-MS**: *m/z* 192 (M^+). **^{1}H NMR** (500 MHz, $(CD_3)_2CO$, 298 K): δ (ppm) 8.82 (d, *J* = 8 Hz, 2H, *a*), 8.36 (t, *J* = 8 Hz, 1H, *c*), 8.01 (dd, *J* = 7,4 Hz, *J* = 1,3 Hz, 2H, *b*). **^{13}C{^{1}H} NMR** (126 MHz, $(CD_3)_2CO$, 298 K): δ (ppm) 141.83, 131.80, 131.60, 114.60. **^{19}F{^{13}C} NMR** (470 MHz, $(CD_3)_2CO$, 298 K): δ (ppm) -72.44 (d, *J* = 706.7 Hz).

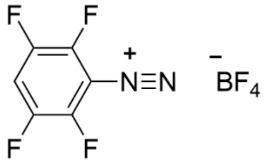

2,3,5,6-tetrafluorobenzenediazonium tetrafluoroborate (3d): **3d** was synthesized according to Ref. 40. **EI-MS**: *m/z* 264 (M^+). **^{1}H NMR** (500 MHz, $(CD_3)_2CO$, 298 K): δ (ppm) 8.85 (m, 1H, *a*). **^{13}C{^{1}H} NMR** (126 MHz, $CD_3)_2CO$, 298 K): δ (ppm) 146.6 (dm, *J* = 273.4 Hz), 144.7 (dm, *J* = 214.2 Hz), 123.6 (t, *J* = 23.9 Hz), 98.2 (t, *J* = 26.5 Hz). **^{19}F{^{13}C} NMR** (470 MHz, $(CD_3)_2CO$, 298 K): δ (ppm) -141.6 (m, 2F), -150.0 (s, 4F), -156.6 (m, 2F).

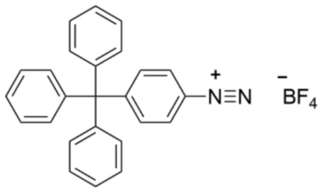

4-tritylbenzenediazonium tetrafluoroborate (4d): **4d** was synthesized according to Ref. 41. **EI-MS**: *m/z* 434 (M^+). **^{1}H NMR** (500 MHz, $(CD_3)_2CO$, 298 K): δ (ppm) 8.78 (d, *J* = 9 Hz, 2H, *a*), 7.99 (d, *J* = 9 Hz, 2H, *b*), 7.33 (m, 15H, *c-d-e*). **^{13}C{^{1}H} NMR** (126 MHz, $(CD_3)_2CO$, 298 K): δ (ppm) 162.14, 144.75, 133.69, 132.42, 130.54, 128.34, 126.86, 112.22, 66.32. **^{19}F{^{13}C} NMR** (470 MHz, $(CD_3)_2CO$, 298 K): δ (ppm) -151.4 (s, 4F).

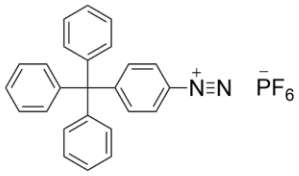

4-tritylbenzenediazonium hexafluorophosphate (5d): **5d** was synthesized according to Ref. 42. **EI-MS**: *m/z* 492 (M^+). **^{1}H NMR** (500 MHz, $(CD_3)_2CO$, 298 K): δ (ppm) 8.79 (d, *J* = 6.5 Hz, 2H, *a*), 8.01 (d, *J* = 9.1 Hz, 2H, *b*), 7.40 - 7.33 (m, 6H, *c-d-e*), 7.31 - 7.18 (m, 9H). **^{13}C{^{1}H} NMR** (126 MHz, $(CD_3)_2CO$, 298 K): δ (ppm) 163.22, 145.65, 134.33, 133.33, 131.44, 129.28, 127.81, 113.06, 66.35. **^{19}F{^{13}C} NMR** (470 MHz, $(CD_3)_2CO$, 298 K): δ (ppm) -72.57 (d, *J* = 707.4 Hz).

General synthetic procedure via azo-coupling

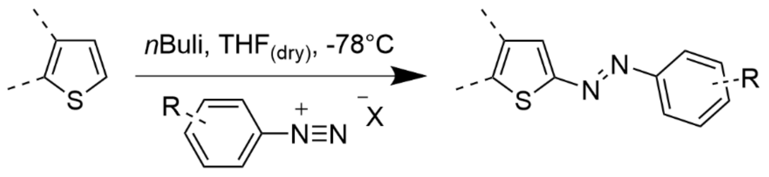

Scheme S1. General synthetic scheme for the preparation of α-oligothiophenes (*E*-**1**–**4**) and oligothienoacenes (*E*-**2a**–**4a**, *E*-**2a**F_4, *E*-**3a**Ph_3) via azo coupling.

A solution of nBuLi in hexane (2.5 M, 1.1 mmol) was added dropwise at -78 °C to a solution of the opportune thiophene derivative (1 mmol) in dry THF (5 mL) under a N_2 atmosphere. Afterward, the solution was stirred for 30 min maintaining the temperature at -78°C. Then, the solution was transferred with a gas-tight syringe to a previously prepared suspension of the diazonium salt (1.2 mmol) in dry THF (5 mL). The resulting deep brown reaction mixture was stirred for 1 hour and left to reach room temperature. The reaction was quenched with distilled water and extracted with DCM (50 mL × 3). The combined organic phases were washed with brine (1 × 80 mL) and dried over Na_2SO_4. The organic solvent was removed under vacuum, and the crude product was purified by flash chromatography over silica (eluent: cyclohexane/CH_2Cl_2) to yield the title compound.

(E)-1-phenyl-2-(thiophen-2-yl)diazene (*E*-**1**): the synthesis was performed according to the general procedure using thiophene (1 mmol) and phenyldiazonium tetrafluoroborate **1d** or the corresponding hexafluorophosphate salt **2d** (1.2 mmol) as synthetic precursors. The pure product was obtained through flash column chromatography over silica (Cyclohexane/CH_2Cl_2 – 90:10) as an orange solid (Yield: X^-=BF_4^-, 90%; X^-=PF_6^-, 55%). **m.p.**: 70 - 71°C. **EI-MS**: *m/z* 188 (M^+). **^{1}H NMR** (500 MHz, $CDCl_3$, 298 K): δ (ppm) 7.88 (m, 2H, *d*), 7.82 (dd, *J* = 3.8 Hz, 1.3 Hz, 1H, *c*), 7.48 (m, 3H, *e-f*), 7.42 (dd, *J* = 5.6 Hz, 1.6 Hz, 1H, *a*), 7.17 (dd, *J* = 5.3, 3.8 Hz, 1H, *b*). **^{13}C{^{1}H} NMR** (126 MHz, $CDCl_3$, 298 K): δ (ppm) 160.39, 152.07, 131.71, 130.78, 129.08, 128.49, 127.46, 122.82.

(E)-1-([2,2'-bithiophen]-5-yl)-2-phenyldiazene (*E*-**2**): the synthesis was performed according to the general procedure using 2,2'-bithiophene (1 mmol) and phenyldiazonium tetrafluoroborate **1d** or the corresponding hexafluorophosphate salt **2d** (1.2 mmol) as synthetic precursors. The pure product was obtained through flash column chromatography over silica (Cyclohexane/CH_2Cl_2 – 85:15) as an orange solid (Yield: X^-=BF_4^-, 80%; X^-=PF_6^-, 45%). **m.p.**: 105 - 110 °C. **EI-MS**: *m/z* 270 (M^+). **^{1}H NMR** (500 MHz, $CDCl_3$, 298 K): δ (ppm) 7.86 (d, *J* = 7.3 Hz, 2H, *f*), 7.72 (d, *J* = 4.1 Hz, 1H, *e*), 7.49 (m, 2H, *g*), 7.43 (m, 1H, *h*), 7.36 (dd, *J* = 3.6, 1.0 Hz, 1H, *d*), 7.32 (dd, *J* = 5.1, 1.0 Hz, 1H, *a*), 7.27 (d, *J* = 4.1 Hz, 1H, *c*), 7.07 (dd, *J* = 5.0, 3.7 Hz, 1H, *b*). **^{13}C{^{1}H} NMR** (126 MHz, $CDCl_3$, 298 K): δ (ppm) 158.31, 152.32, 140.72, 137.58, 133.02, 130.79, 129.26, 128.38, 126.37, 125.47, 123.82, 122.93.

(E)-1-([2,2':5',2''-terthiophen]-5-yl)-2-phenyldiazene (*E*-**3**): the synthesis was performed according to the general procedure using 2,2':5',2''-terthiophene (1 mmol) and phenyldiazonium

tetrafluoroborate **1d** or the corresponding hexafluorophosphate salt (1.2 mmol) as synthetic precursors. The pure product was obtained through flash column chromatography over silica (Cyclohexane/CH_2Cl_2 – 75:25) as an orange-red solid (Yield: X^-=BF_4^-, 65%; X^-=PF_6^-, 30%). **m.p.**: 162 - 164 °C. **EI-MS**: *m/z* 352 (M^+). **^{1}H NMR** (500 MHz, CD_2Cl_2, 298 K): δ (ppm) 7.85 (d, *J* = 7.3 Hz, 2H, *h*), 7.72 (d, *J* = 4.1 Hz, 1H, *g*), 7.45 (m, 3H, *i-j*), 7.26 (m, 3H, *d-e-f*), 7.22 (d, *J* = 3.6, 1.0 Hz, 1H, *a*), 7.13 (d, *J* = 3.8 Hz, 1H, *c*), 7.04 (dd, *J* = 5.1, 3.6 Hz, 1H, *b*). **^{13}C{^{1}H} NMR** (126 MHz, CD_2Cl_2, 298 K): δ (ppm) 158.09, 152.17, 140.15, 138.20, 136.59, 135.82, 133.03, 130.68, 129.14, 128.04, 126.18, 125.21, 124.63, 124.29, 123.67, 122.64.

(E)-1-([2,2':5',2''-terthiophen]-5-yl)-2-(4-tritylphenyl)diazene (*E*-**3**Ph_3): the synthesis was performed according to the general procedure using 2,2':5',2''-terthiophene (1mmol) and 4-tritylbenzenediazonium tetrafluoroborate **4d** or the corresponding hexafluorophosphate salt **5d** (1.2 mmol) as synthetic precursors. The crude product was purified by column chromatography over silica (cyclohexane/DCM/AcOEt – 80:15:5) to yield a pure reddish orange solid (Yield: X^-=BF_4^-, 50%; X-=PF_6^-, <5%). **m.p.**: 235 - 238 °C. **EI-MS**: m/z 594 (M+). **^{1}H NMR** (500 MHz, $CDCl_3$, 298 K): δ (ppm) 7.73 (d, J = 9 Hz, 2H, h), 7.68 (d, J = 4 Hz, 1H, g), 7.36 (d, J = 9 Hz, 2H, i), 7.27 (m, Hz, 4H, a-d-e-f), 7.22 (m, 15H, j-k-l), 7.12 (d, J = 3.5 Hz, 1H, c), 7.04 (dd, J = 5, 3.5 Hz, 1H, b). **^{13}C{^{1}H} NMR** (126 MHz, CDCl3, 298 K): δ (ppm) 149.77, 146.36, 136.75, 136.00, 132.76, 131.89, 131.06, 128.01, 127.61, 126.07, 125.10, 124.64, 124.22, 123.60, 121.82, 65.09.

*(E)-1-([2,2':5',2'':5'',2'''-quaterthiophen]-5-yl)-2-phenyldiazene (E-**4**)*: the synthesis was performed according to the general procedure using 2,2':5',2'':5'',2'''-quaterthiophene (1 mmol) and phenyldiazonium tetrafluoroborate **1d** or the corresponding hexafluorophosphate salt **2d** (1.2 mmol) as synthetic precursors. The pure residue was purified by flash chromatography over silica (Cyclohexane/CH_2Cl_2 – 60:40) and precipitation in cold pentane. Deep red solid. (Yield: X^-=BF_4^-, 5 %; X^-=PF_6^-, traces). **m.p.**: 213 - 214 °C. **EI-MS**: *m/z* 434 (M^+). **^{1}H NMR** (500 MHz, DMSO, 343 K): δ (ppm) 7.93 (d, *J* = 4.1 Hz, 1H, *i*), 7.81 (d, *J* = 7.6 Hz, 2H, *j*), 7.56 (m, 6H, *f-g-h-k-l*), 7.38 (m, 3H, *a-d-e*), 7.30 (d, *J* = 3.8 Hz, 1H, *c*), 7.12 (dd, *J* = 5.0, 3.7 Hz, 1H, *b*). **^{13}C{^{1}H} NMR** not recorded due to low solubility.

(E)-1-phenyl-2-(thieno[3,2-b]thiophen-2-yl)diazene (*E*-**2a**): the synthesis was performed according to the general procedure using thieno[3,2-b]thiophene (1 mmol) and phenyldiazonium tetrafluoroborate **1d** or the corresponding hexafluorophosphate salt **2d** (1.2 mmol) as synthetic precursors. The pure product was obtained through flash column chromatography over silica (Cyclohexane/CH_2Cl_2 – 85:15) as an orange solid (Yield: X^-=BF_4^-, 85%; X^-=PF_6^-, 50%). **m.p.**: 156 - 158 °C. **EI-MS**: *m/z* 244 (M^+). **^{1}H NMR** (500 MHz, $CDCl_3$, 298 K): δ (ppm) 8.01 (s, 1H, *c*), 7.88 (d, *J* = 7.5 Hz, 2H, *d*), 7.55 (d, *J* = 5.2 Hz, 1H, *a*), 7.50 (m, 2H, *e*), 7.47 – 7.41 (m, 1H, *f*), 7.28 (d, *J* = 5.2 Hz, 1H, *b*). **^{13}C{^{1}H} NMR** (126 MHz, $CDCl_3$, 298 K): δ (ppm) 161.53, 152.20, 140.52, 138.46, 130.92, 130.89, 129.26, 125.11, 123.04, 120.82.

*(E)-1-(2,3,5,6-tetrafluorophenyl)-2-(thieno[3,2-b]thiophen-2-yl)diazene (E-***2a**F_4): the synthesis was performed according to the general procedure using thieno[3,2-b]thiophene (1 mmol) and 2,3,5,6-tetrafluorobenzenediazonium tetrafluoroborate **3d** (1.2 mmol) as synthetic precursors. The crude product was purified by column chromatography over silica (cyclohexane/DCM – 90:10) to yield pure deep red solid (Yield: X^-=BF_4^-, 75%). **m.p.**: 168 - 170 °C. **EI-MS**: *m/z* 316 (M^+). **^{1}H NMR** (500 MHz, $CDCl_3$, 298 K): δ (ppm) 8.15 (s, 1H, *c*), 7.65 (d, *J* = 5 Hz, 1H, *a*), 7.30 (d, *J* = 5 Hz, 1H, *b*), 7.08 (m, 1H, *d*). **^{13}C{^{1}H} NMR** (126 MHz, $CDCl_3$, 298 K): δ (ppm) 161.00 (s), 146.2 (dm, *J* = 256.9 Hz), 142.8 (s), 140.6 (dm, *J* = 259.5 Hz), 138.82 (s), 128.65 (s), 120.74 (s), 105.41 (t, *J* = 23.9 Hz). **^{19}F{^{13}C} NMR** (470 MHz, $CDCl_3$, 298 K): δ (ppm) -139 (m, 2F), -149 (m, 2F).

(E)-1-(dithieno[3,2-b:2',3'-d]thiophen-2-yl)-2-phenyldiazene (*E*-**3a**): the synthesis was performed according to the general procedure using dithieno[3,2-b:2',3'-d]thiophene (1 mmol) and phenyldiazonium tetrafluoroborate **1d** or the corresponding hexafluorophosphate salt **2d** (1.2 mmol) as synthetic precursors. The pure product was obtained through flash column chromatography over silica (Cyclohexane/CH_2Cl_2 – 75:25) as an orange solid (Yield: X^-=BF_4^-, 70%; X^-=PF_6^-, 40%). **m.p.**: 190 - 193 °C. **EI-MS**: *m/z* 300 (M^+). **^{1}H NMR** (500 MHz, $CDCl_3$, 298 K): δ (ppm) 8.02 (s, 1H, *c*), 7.88 (d, *J* = 7.4 Hz, 2H, *d*), 7.51 (m, 3H, *e-a*), 7.44 (m, 1H, *f*), 7.33 (d, *J* = 5.2 Hz, 1H, *b*). **^{13}C{^{1}H} NMR** (126 MHz, $CDCl_3$, 298 K): δ (ppm) 160.15, 152.17, 144.11, 140.70, 132.76, 132.08, 130.91, 129.29, 128.99, 126.21, 123.05, 120.99.

(E)-1-(dithieno[3,2-b:2',3'-d]thiophen-2-yl)-2-(4-tritylphenyl)diazene (*E*-**3a**Ph_3): the synthesis was performed according to the general procedure using dithieno[3,2-b:2',3'-d]thiophene (1mmol) and 4-tritylbenzenediazonium tetrafluoroborate **4d** or the corresponding hexafluorophosphate salt **5d** (1.2 mmol) as synthetic precursors. The crude product was purified by column chromatography over silica (cyclohexane/DCM – 90:10) to yield a pure orange solid (Yield: X^-=BF_4^-, 70%; X^-=PF_6^-, 5%). **m.p.**: 224 - 226 °C. **EI-MS**: *m/z* 542 (M^+). **^{1}H NMR (**500 MHz, $CDCl_3$, 298 K): δ (ppm) 7.97 (s, 1H, *c*), 7.75 (d, *J* = 8.7 Hz, 2H, *d*), 7.49 (d, *J* = 5.2 Hz, 1H, *a*), 7.38 (d, *J* = 8.7 Hz, 2H, *e*), 7.32 (d, *J* = 5.2 Hz, 1H, *b*), 7.23 (m, 15H, *f-g-h*). **^{13}C{^{1}H} NMR (**126 MHz, $CDCl_3$, 298 K): δ (ppm) 160.31, 150.12, 150.07, 146.52, 144.02, 140.72, 132.65, 132.07, 131.22, 128.92, 127.80, 127.59, 126.25, 125.96, 122.11, 120.97, 65.27

(E)-1-phenyl-2-(thieno[2',3':4,5]thieno[3,2-b]thieno[2,3-d]thiophen-2-yl)diazene (*E*-**4a**): the synthesis was performed according to the general procedure using thieno[2',3':4,5]thieno[3,2-b]thieno[2,3-d]thiophene (1 mmol) and phenyldiazonium tetrafluoroborate **1d** or the corresponding hexafluorophosphate salt **2d** (1.2 mmol) as synthetic precursors. The pure product was obtained through flash column chromatography over silica (Cyclohexane/CH_2Cl_2 – 70:30), followed by crystallization in cold pentane, as a red solid (Yield: X^-=BF_4^-, 20%; X^-=PF_6^-, 5%). **m.p.**: 238 - 240 °C. **EI-MS**: *m/z* 356 (M^+). **^{1}H NMR** (500 MHz, DMSO, 373 K): δ (ppm) 8.44 (s, 1H, *c*), 7.83 (d, *J* = 8.0 Hz, 3H, *d*), 7.56 (m, 4H, *a-b-e-f*). **^{13}C{^{1}H} NMR** not recorded due to low solubility.

General synthetic procedure of phenylazo α-oligothiophenes via Suzuki-Miyaura cross-coupling

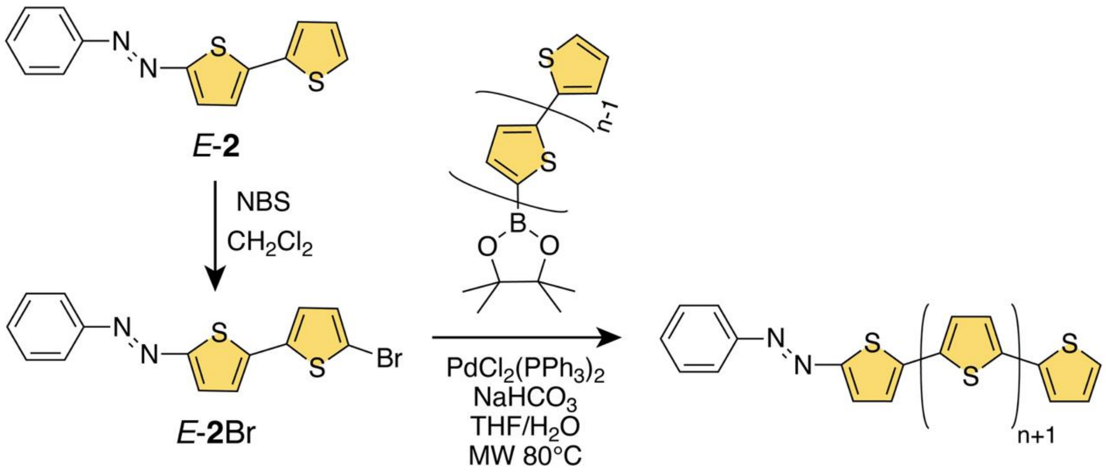

Scheme S2. General synthetic scheme for the preparation of α-oligothiophenes *via* Suzuki-Miyaura cross-coupling.

Compounds *E*-**3** and *E*-**4** were prepared from brominated phenylazothiophene precursor *E*-**2**Br (1 mmol), the appropriate dioxaborolane derivative (1 mmol), $NaHCO_3$ (5 mmol) and Pd(dppf)Cl_2 (0.005 mmol) in THF/H_2O mixture (2:1, 3 mL of) under microwave (MW) irradiation for 20 min. After returning to room temperature the reaction mixture was diluted with water and extracted with CH_2Cl_2. The combined organic layers were dried and evaporated under reduced pressure. The crude residues were purified by flash chromatography as described above. Yields: 80% for *E*-**3** and 50 % for *E*-**4**.

Procedure for the synthesis of E-2Br

(E)-1-(5'-bromo-[2,2'-bithiophen]-5-yl)-2-phenyldiazene (*E*-**2**Br): N-Bromosuccinimide (1 mmol) was added in small portions to a solution of *E*-**2** (1 mmol) in DCM (5 mL). TLC monitoring (SiO_2, CH_2Cl_2/Hexane 3:7) confirmed the completion of the reaction after 3 hours. The resulting mixture was quenched with water and extracted with DCM (20mL × 3). The crude product was purified by column chromatography over silica (cyclohexane/ CH_2Cl_2 - 80/20) to yield the title compound as a red solid (Yield: 100%). **EI-MS**: *m/z* 348 (M^+). **^{1}H NMR** (500 MHz, $CDCl_3$, 298 K): δ (ppm) 7.72 (d, *J* = 8 Hz, 2H, *e*), 7.65 (d, *J* = 4 Hz, 1H, *d*), 7.36 (d, *J* = 8 Hz, 2H, *f*), 7.22 (m, 2H, *c-g*), 7.07 (d, *J* = 3.9 Hz, 1H, *b*), 7.01 (d, *J* = 3.9 Hz, 1H, *a*). **^{13}C{^{1}H} NMR** (126 MHz, $CDCl_3$, 298 K): δ (ppm) 150.04, 132.49, 131.90, 131.05, 127.64, 126.09, 125.24, 123.80, 121.90, 113.00.

Table S1. Isolated yields for the synthesis of arylazo α-oligothiophenes and oligothienoacenes

Compound	***Yield*** *via* BF_4^- diazonium salt [%]	***Yield*** *via* PF_6^- diazonium salt [%]	***Yield*** *via* Suzuki-Miyaura cross-coupling [%]
E-**1**	90	55	–
E-**2**	80	45	–
E-**3**	65	30	80
E-**4**	<10	traces	50
E-**2a**	85	55	–
E-**3a**	70	40	–
E-**4a**	30	10	–
E-**2a**F_4	75	–	–
E-**3a**Ph_3	70	< 10	–
E-**3**Ph_3	50	< 10	–

IV. NMR characterization

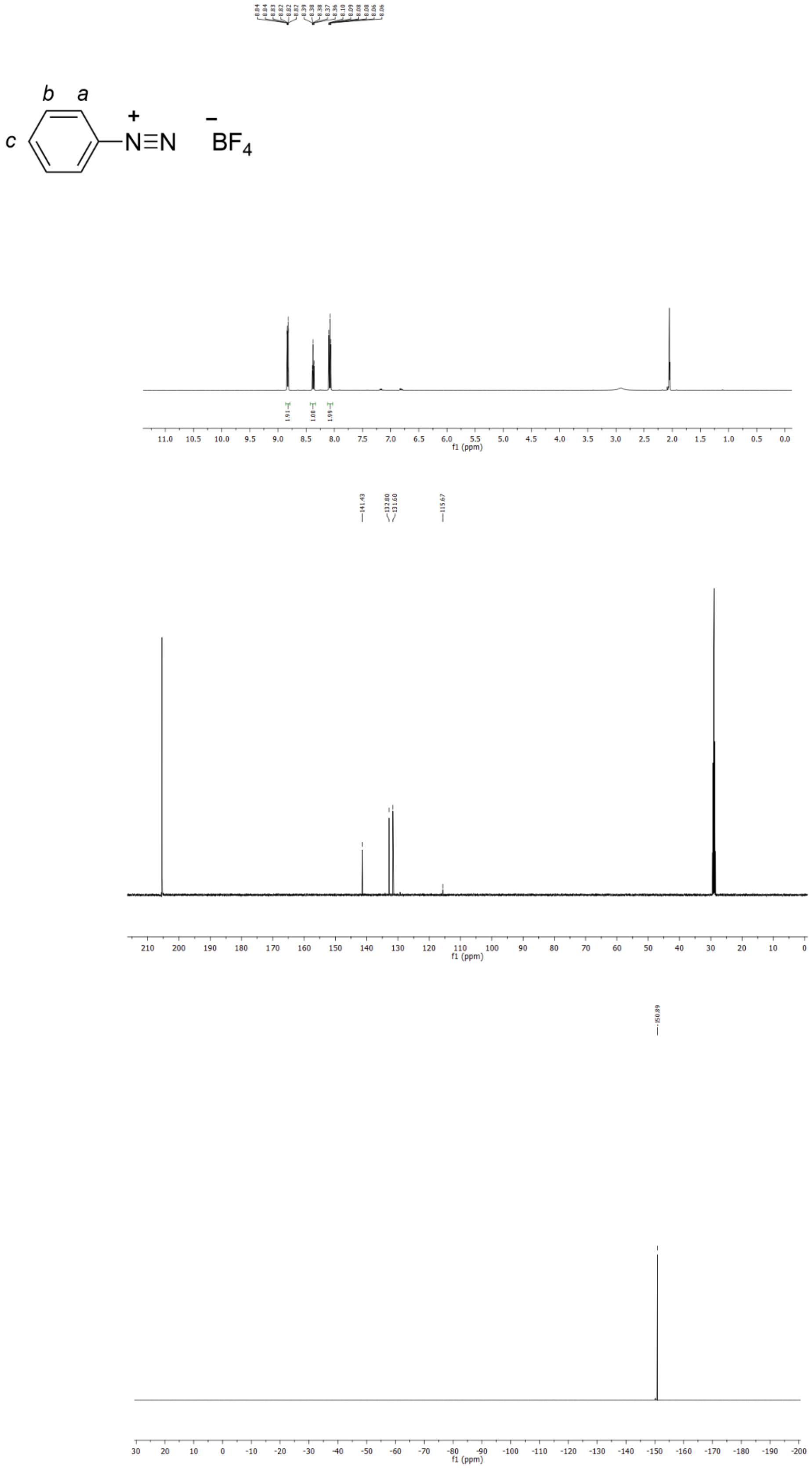

Figure S1. ^{1}H (500 MHz, acetone-*d6*, 298K), ^{13}C{^{1}H} (125 MHz, acetone-*d6*, 298K) and ^{19}F{^{13}C} (470 MHz, acetone-*d6*, 298K) NMR spectra of compound **1d**.

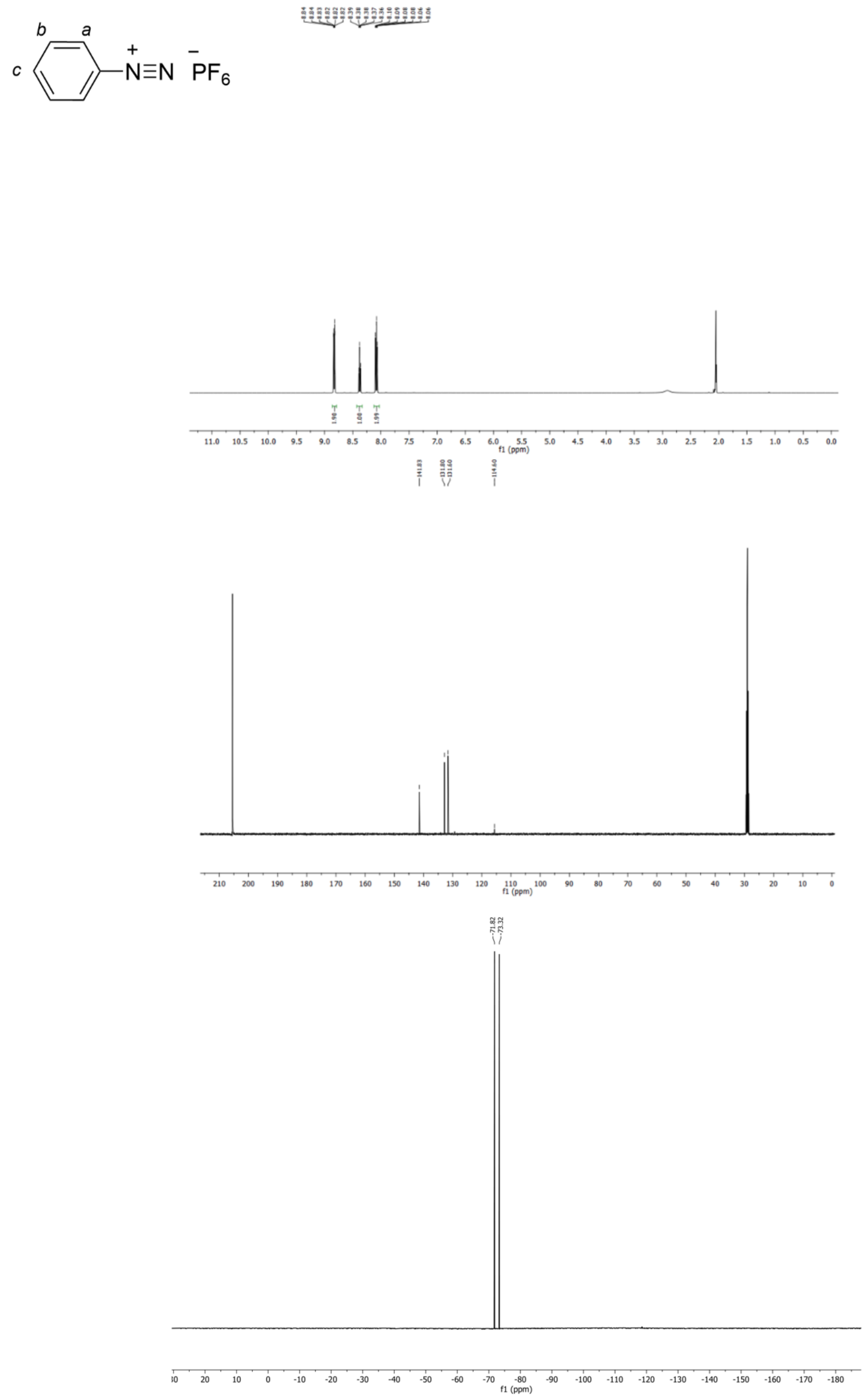

Figure S2. ^{1}H (500 MHz, acetone-*d6*, 298K), $^{13}C\{^{1}H\}$ (125 MHz, acetone-*d6*, 298K) and $^{19}F\{^{13}C\}$ (470 MHz, acetone-*d6*, 298K) NMR spectra of compound **2d**.

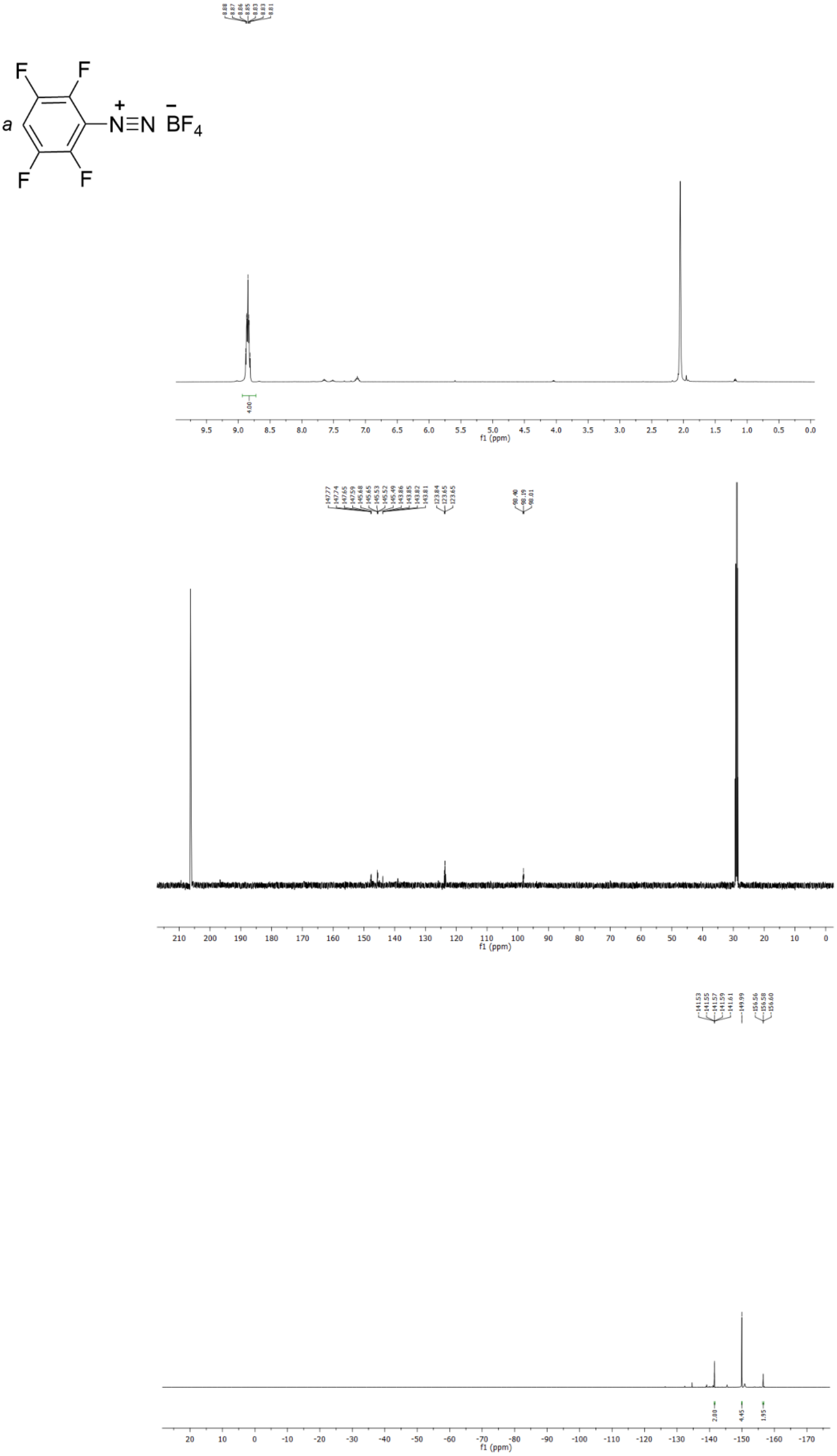

Figure S3. ^{1}H (500 MHz, acetone-*d6*, 298K), $^{13}C\{^{1}H\}$ (125 MHz, acetone-*d6*, 298K) and $^{19}F\{^{13}C\}$ (470 MHz, acetone-*d6*, 298K) NMR spectra of compound **3d**.

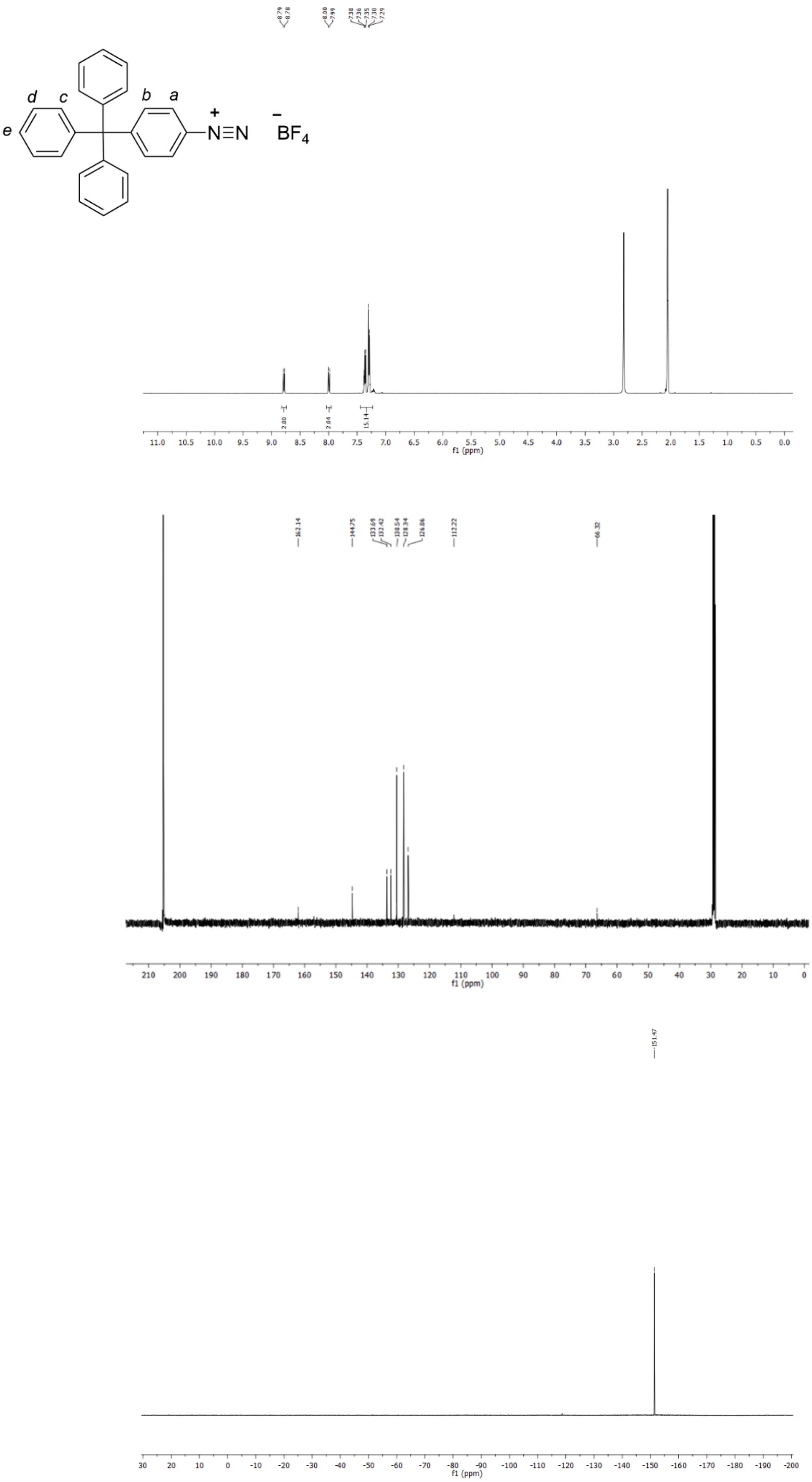

Figure S4. ¹H (500 MHz, acetone-*d6*, 298K), ¹³C{¹H} (125 MHz, acetone-*d6*, 298K) and ¹⁹F{¹³C} (470 MHz, acetone-*d6*, 298K) NMR spectra of compound **4d**.

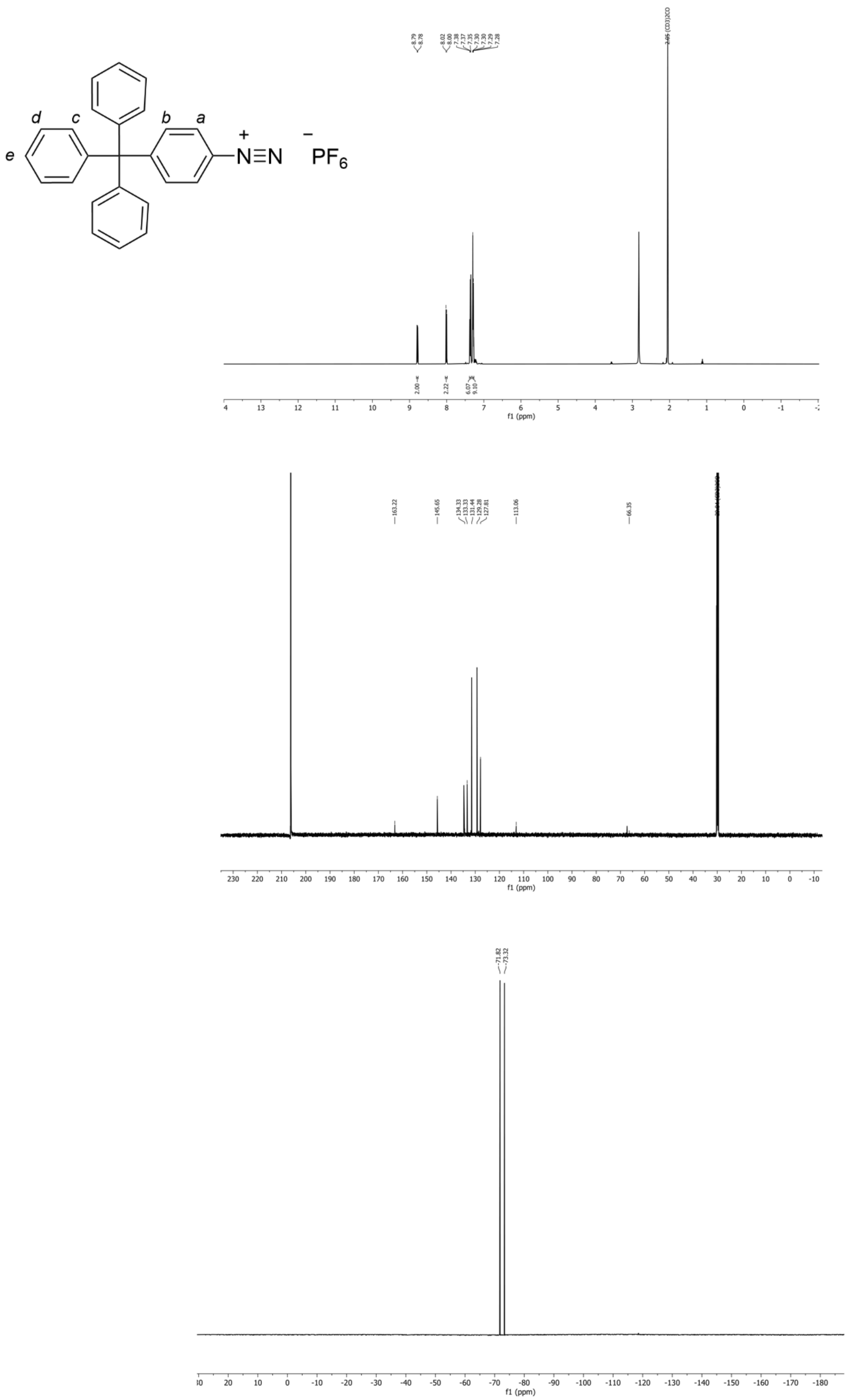

Figure S5. ^{1}H (500 MHz, acetone-*d6*, 298K), $^{13}C\{^{1}H\}$ (125 MHz, acetone-*d6*, 298K) and $^{19}F\{^{13}C\}$ (470 MHz, acetone-*d6*, 298K) NMR spectra of compound **5d**.

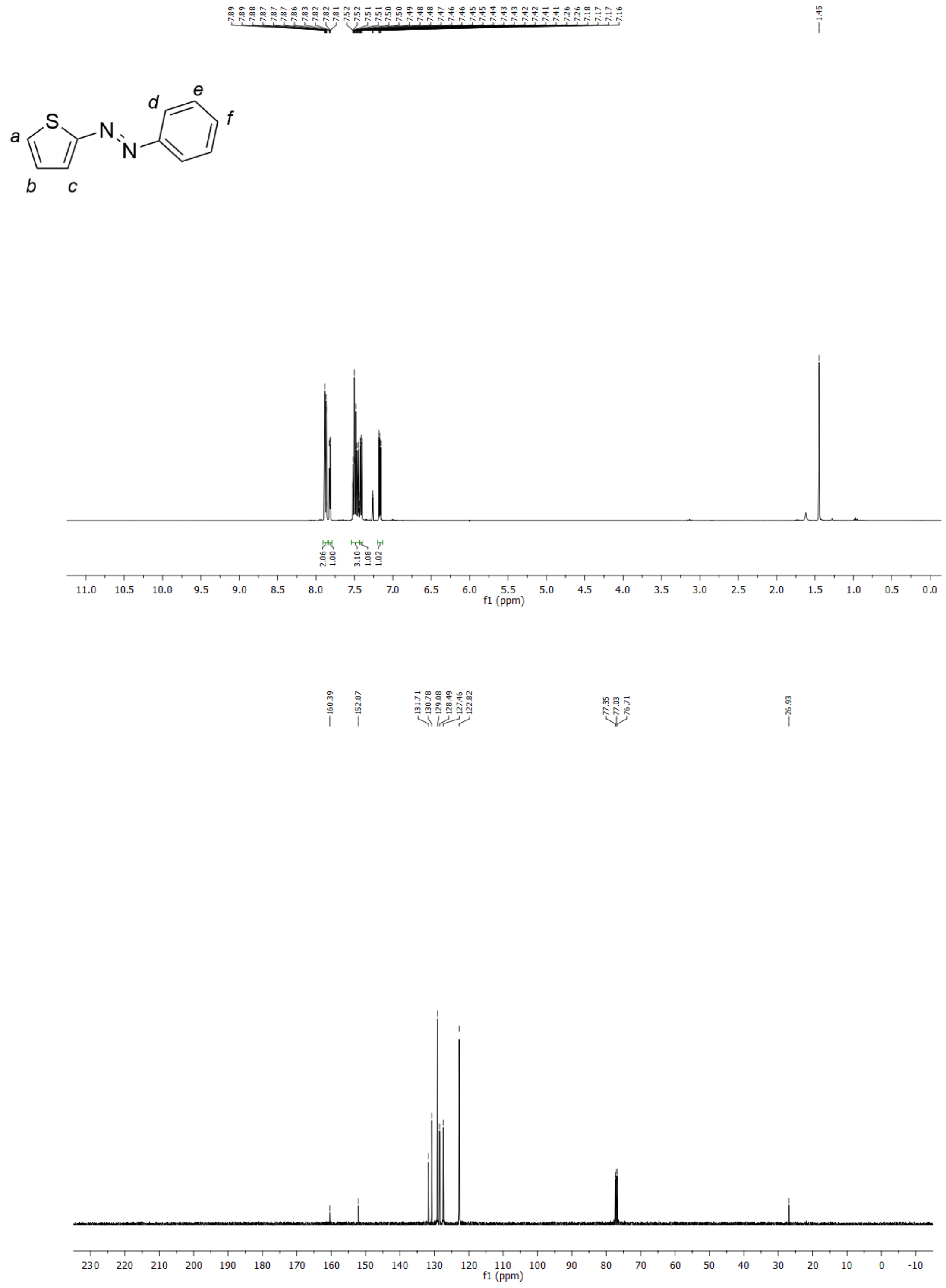

Figure S6. ^{1}H (500 MHz, $CDCl_3$, 298K) and $^{13}C\{^{1}H\}$ (125 MHz, $CDCl_3$, 298K) NMR spectra of compound *E*-**1**.

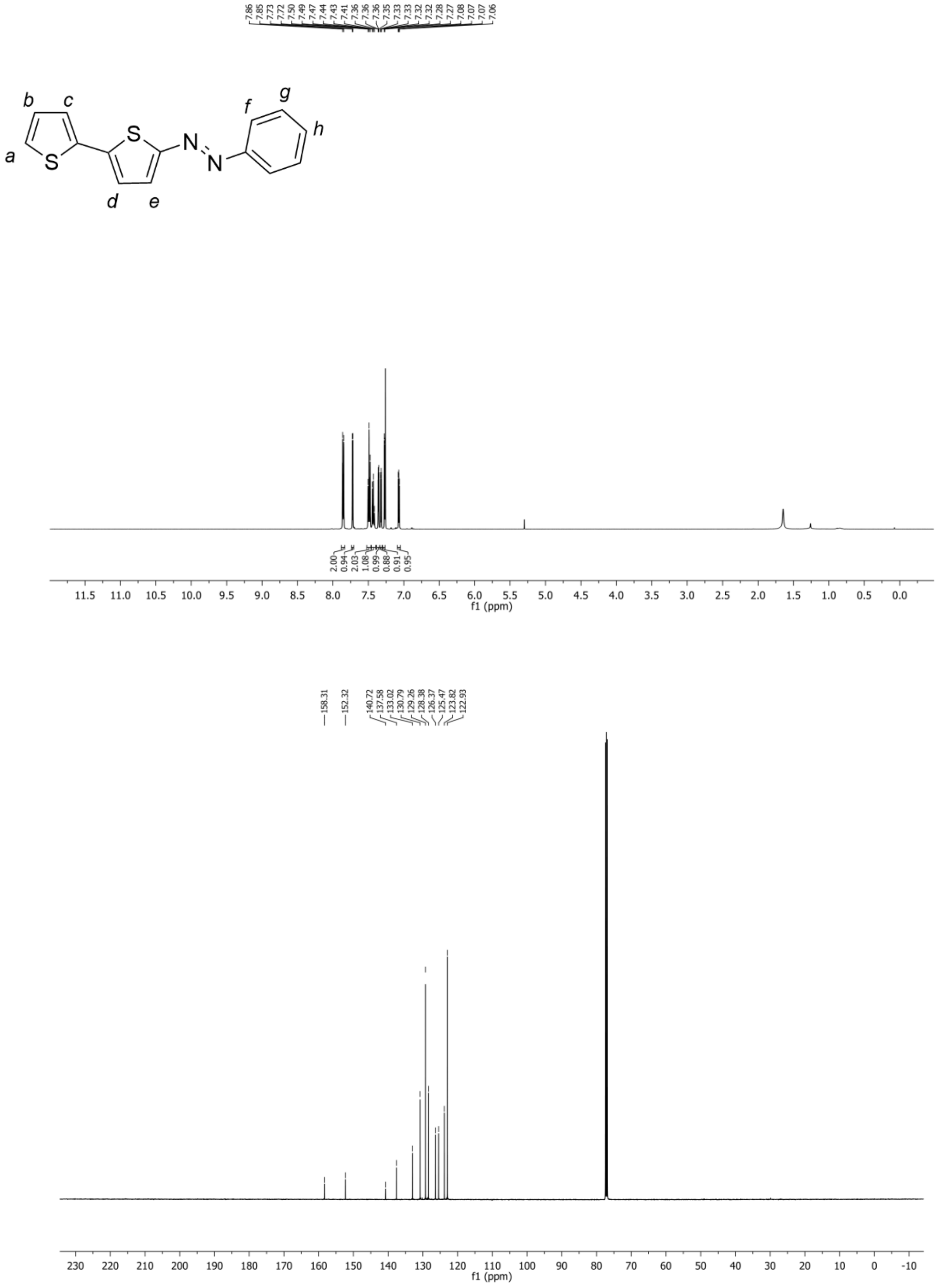

Figure S7. ^{1}H (500 MHz, $CDCl_3$, 298K) and $^{13}C\{^{1}H\}$ (125 MHz, $CDCl_3$, 298K) spectra of compound *E*-**2**.

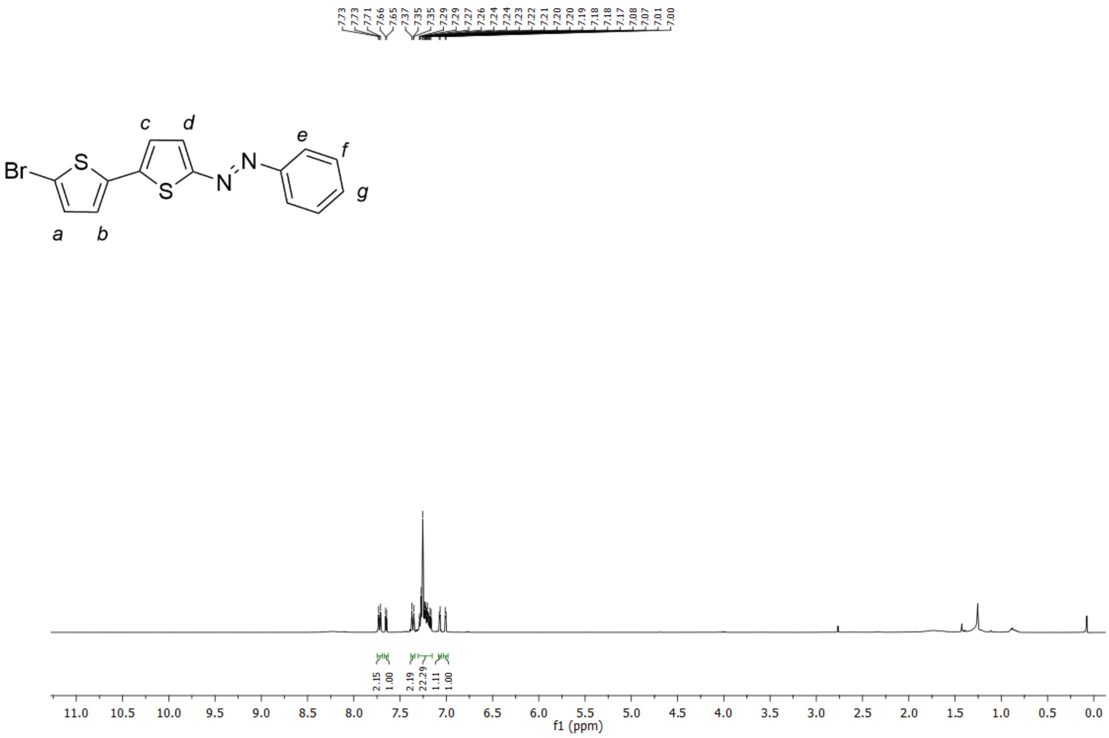

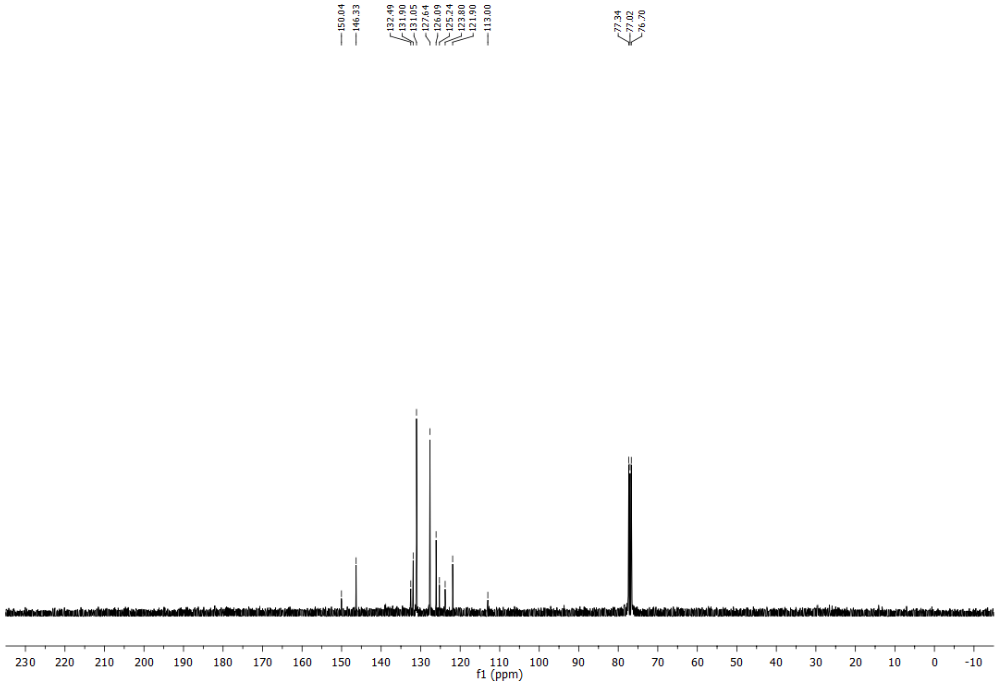

Figure S8. ^{1}H (500 MHz, $CDCl_3$, 298K) NMR and $^{13}C\{^{1}H\}$ (125 MHz, $CDCl_3$, 298K) spectra of compound *E*-**2**Br.

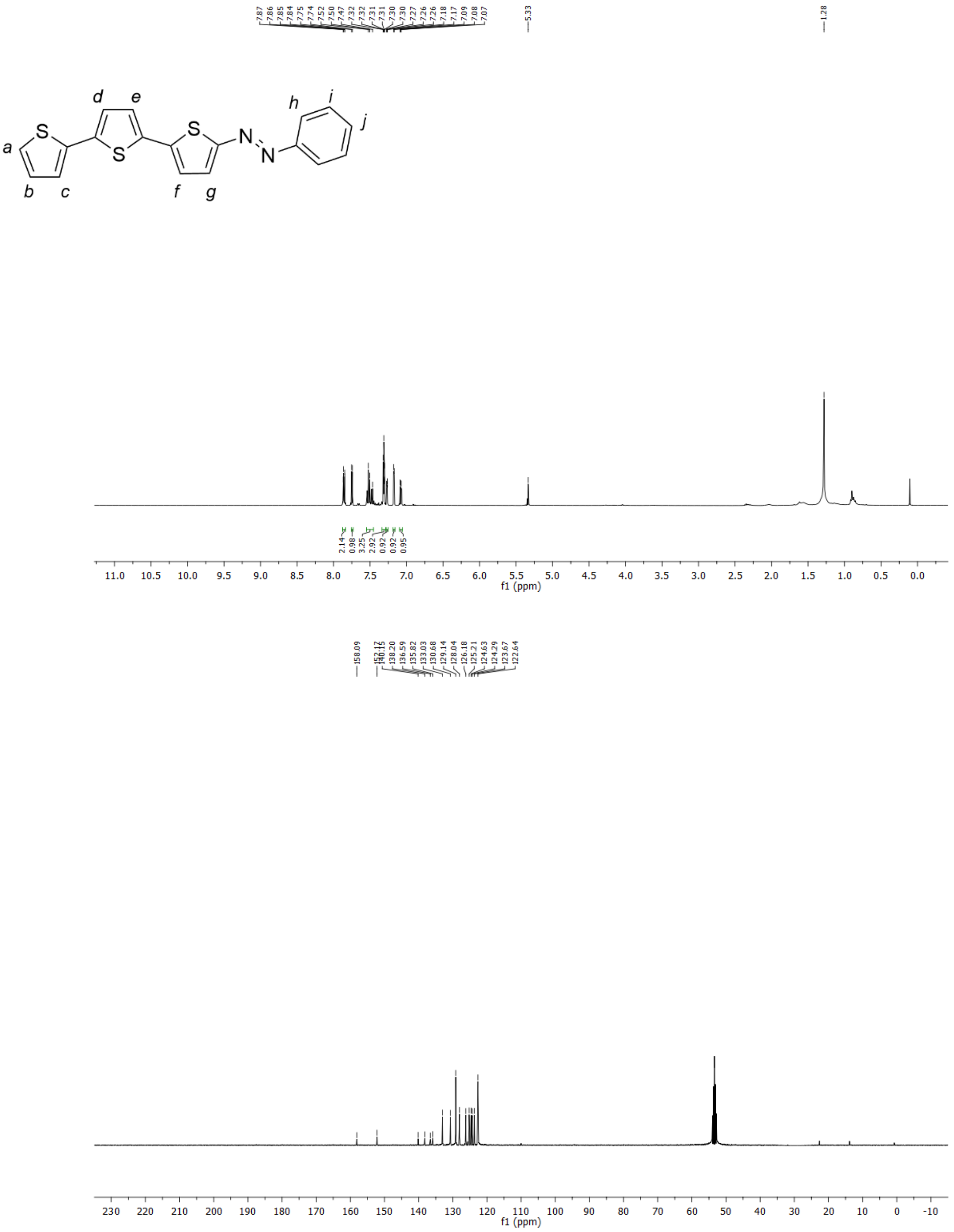

Figure S9. ^{1}H (500 MHz, $CDCl_3$, 298K) and $^{13}C\{^{1}H\}$ (125 MHz, $CDCl_3$, 298K) spectra of compound *E*-**3**

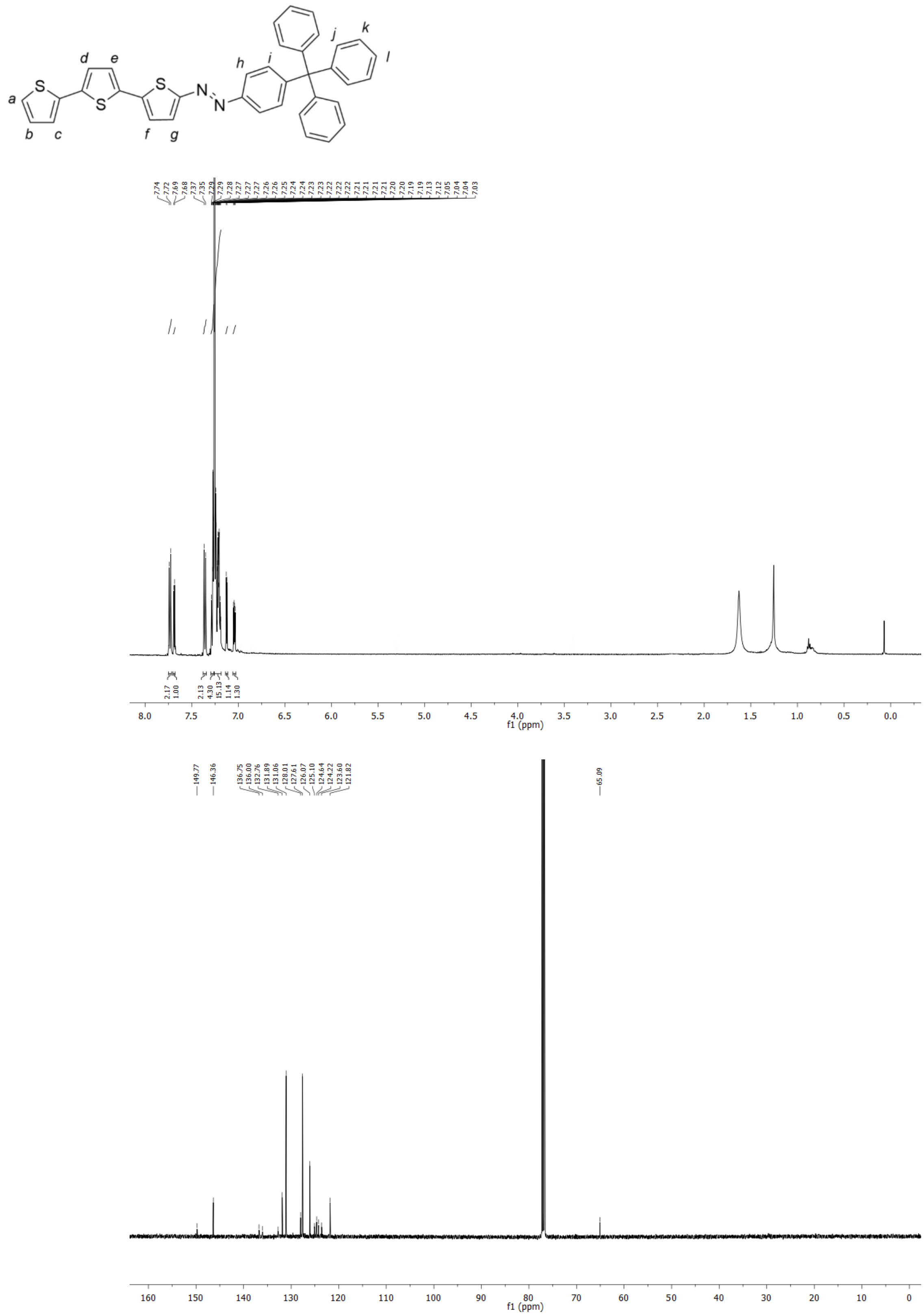

Figure S10. ^{1}H (500 MHz, CDCl3, 298K) and $^{13}C\{^{1}H\}$ (125 MHz, CDCl3, 298K) spectra of compound *E*-**3**Ph3

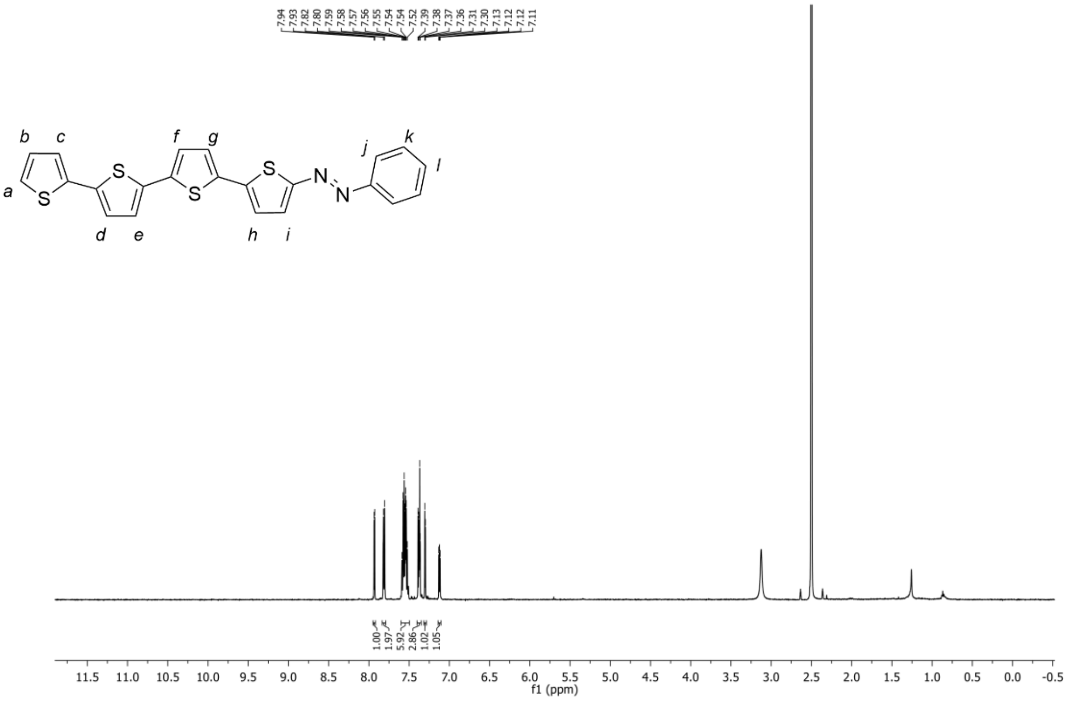

Figure S11. ^{1}H (500 MHz, DMSO-d_6, 373K) NMR spectra of compound *E*-**4**

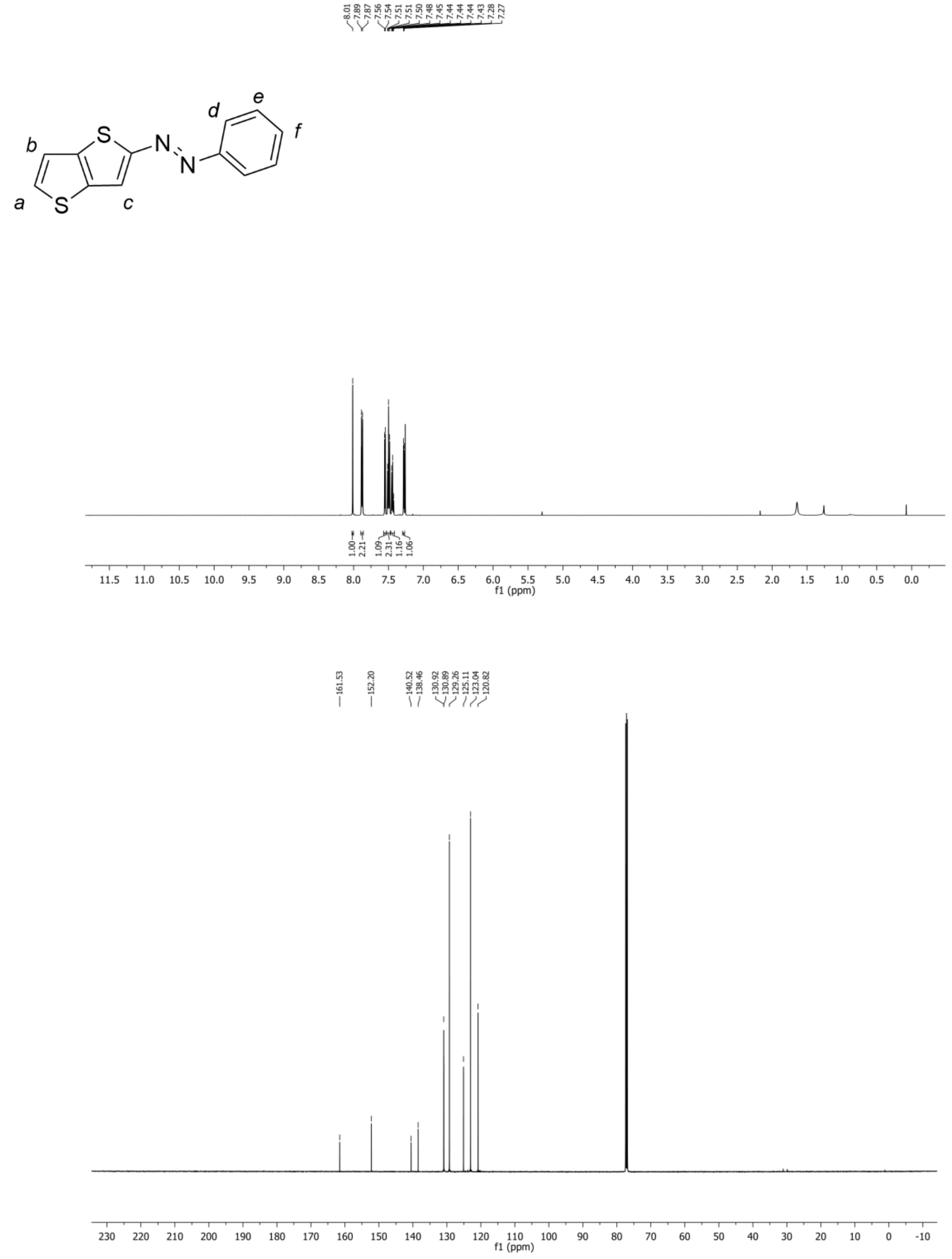

Figure S12. ^{1}H (500 MHz, $CDCl_3$, 298K) and $^{13}C\{^{1}H\}$ (125 MHz, $CDCl_3$, 298K) spectra of compound *E*-**2a**

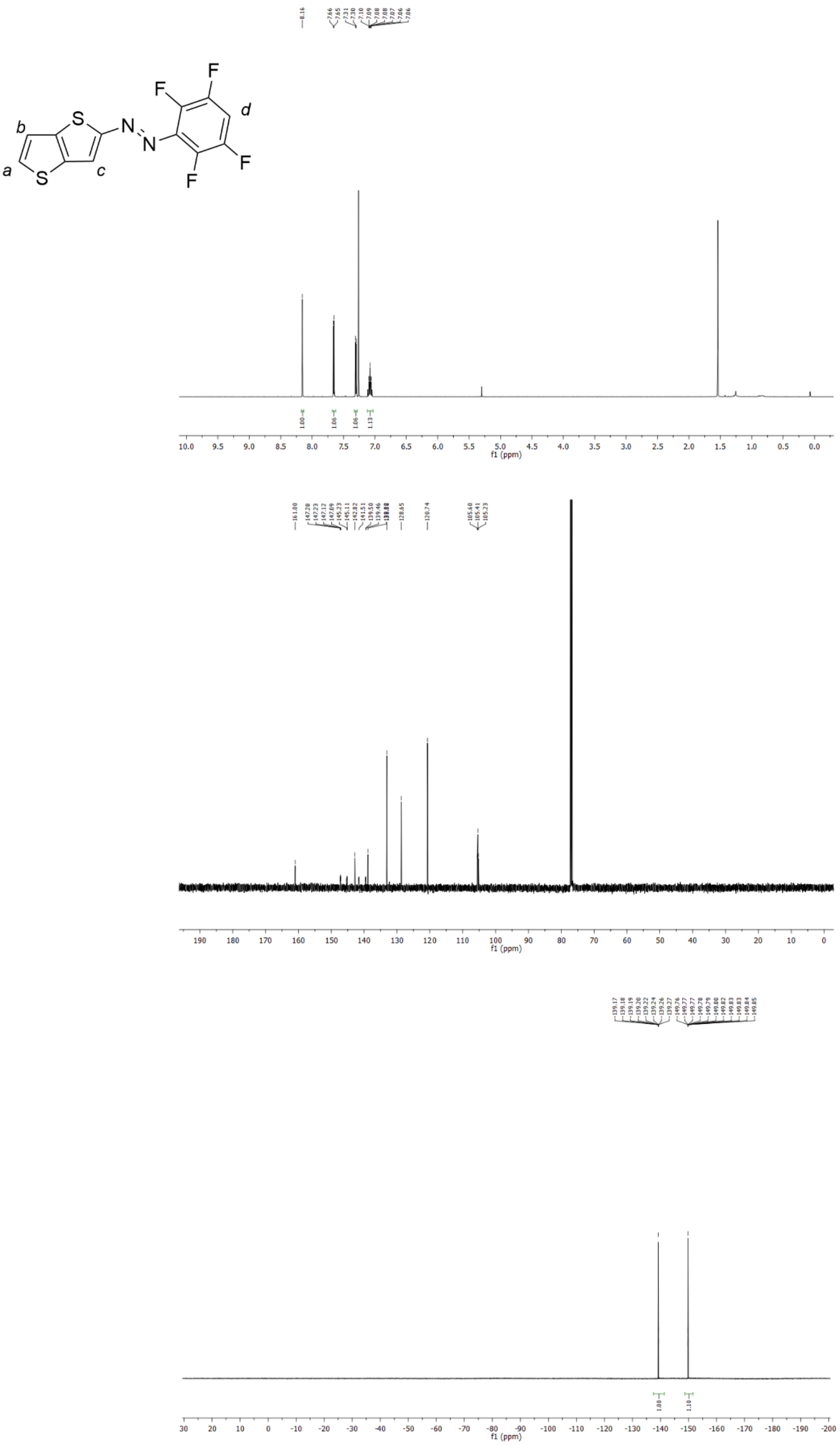

Figure S13. ^{1}H (500 MHz, $CDCl_3$, 298K), $^{13}C\{^{1}H\}$ (125 MHz, $CDCl_3$, 298K) and $^{19}F\{^{13}C\}$ (470 MHz, $CDCl_3$, 298K) NMR spectra of compound *E*-**2a**F_4

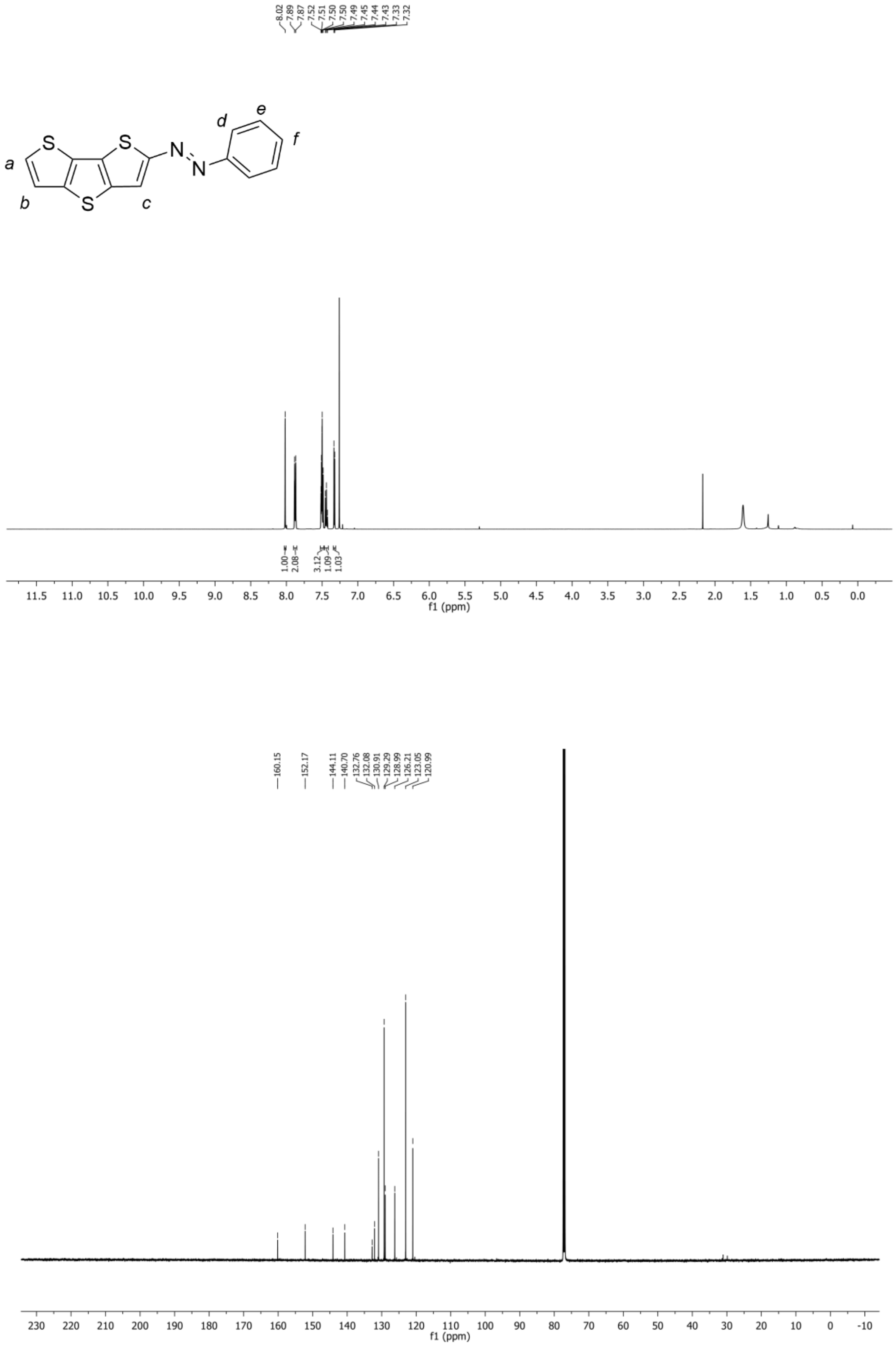

Figure S14. ^{1}H (500 MHz, $CDCl_3$, 298K) and $^{13}C\{^{1}H\}$ (125 MHz, $CDCl_3$, 298K) spectra of compound *E*-**3a**

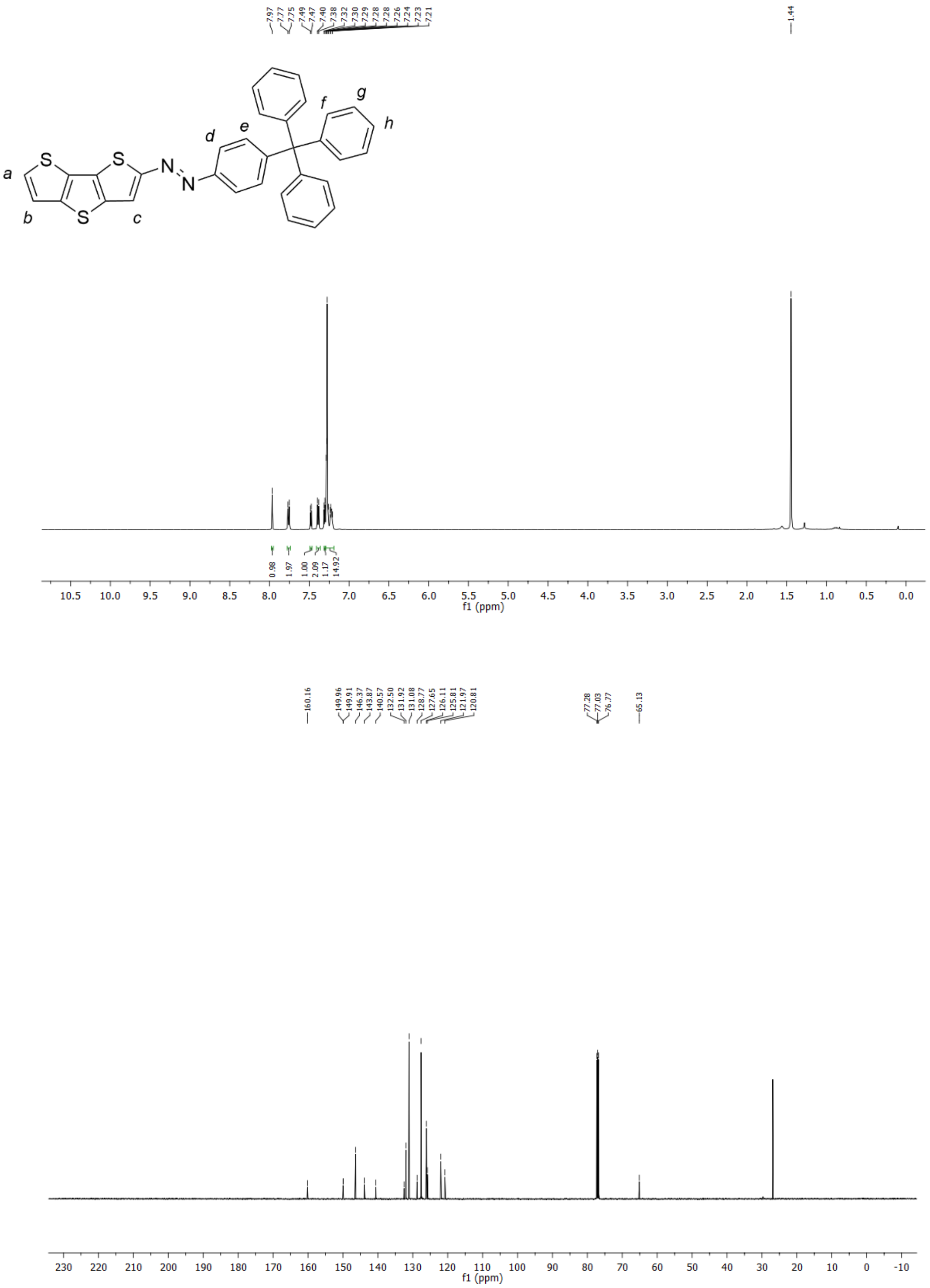

Figure S15. ^{1}H (500 MHz, $CDCl_3$, 298K) and $^{13}C\{^{1}H\}$ (125 MHz, $CDCl_3$, 298K) spectra of compound *E*-**3a**Ph_3

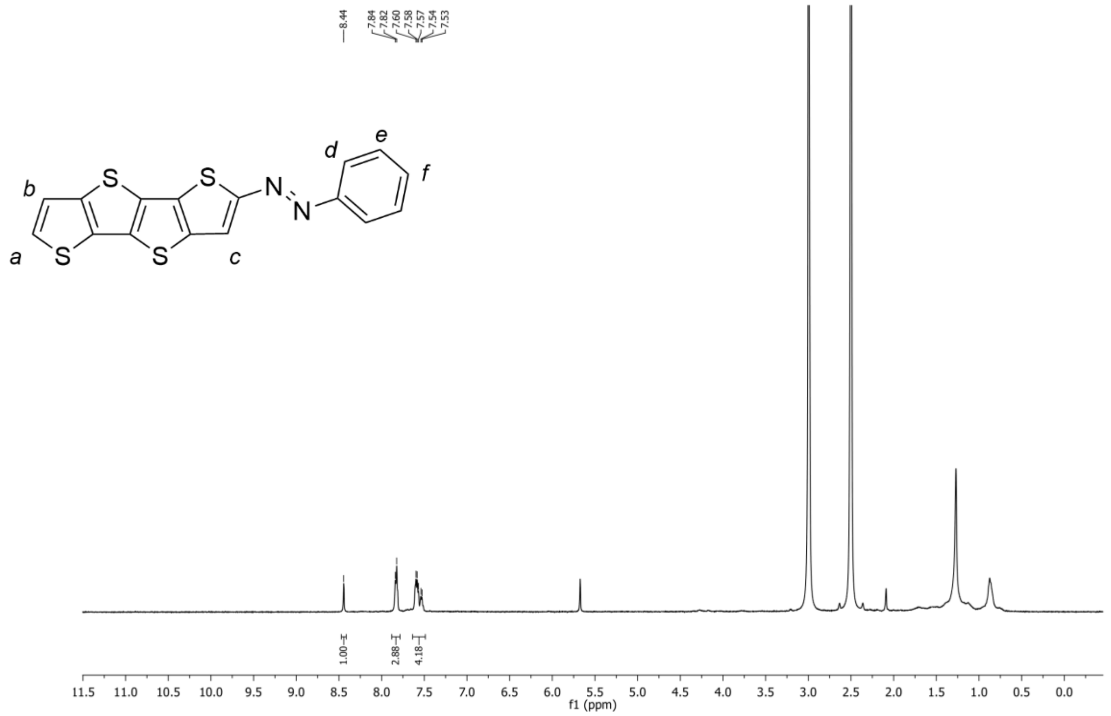

Figure S16. ^{1}H (500 MHz, DMSO-d_6, 373K) NMR spectra of compound *E*-**4a**

V. Theoretical calculations

Table S2. Selected structural parameters of the fully optimized ground-state geometries, obtained at the ωB97M-D4/def2-TZVP level in dichloromethane (using CPCM), of the lowest-energy *E*-isomers of the investigated α-linked oligothiophenes (**1**–**4**).

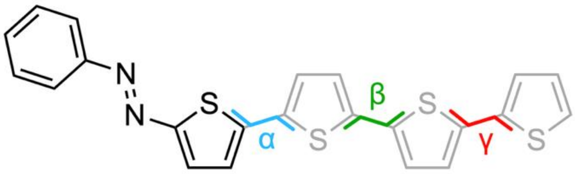

Compound	***α*** [°]	***β*** [°]	***γ*** [°]
E-**1**	–	–	–
E-**2**	157.6	–	–
E-**3**	161.3	153.6	–
E-**4**	160.3	155.2	154.0

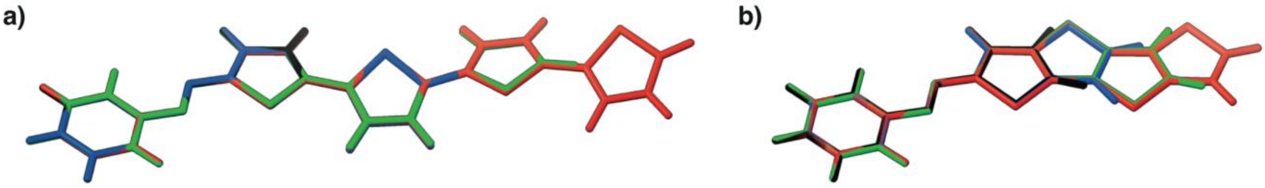

Figure S17. DFT-optimized ground-state geometries of the *E* isomers of: (a) phenylazo oligothiophenes, and (b) arylazo oligothienoacenes. Ground-state geometries overlays were generated by maximizing the overlap between heavy atoms of the phenyl–N=N–thiophenyl fragment. Color legend: *E*-**1**, black; *E*-**2** and *E*-**2a**, cyan; *E*-**3** and *E*-**3a**, green; *E*-**4** and *E*-**4a**, red.

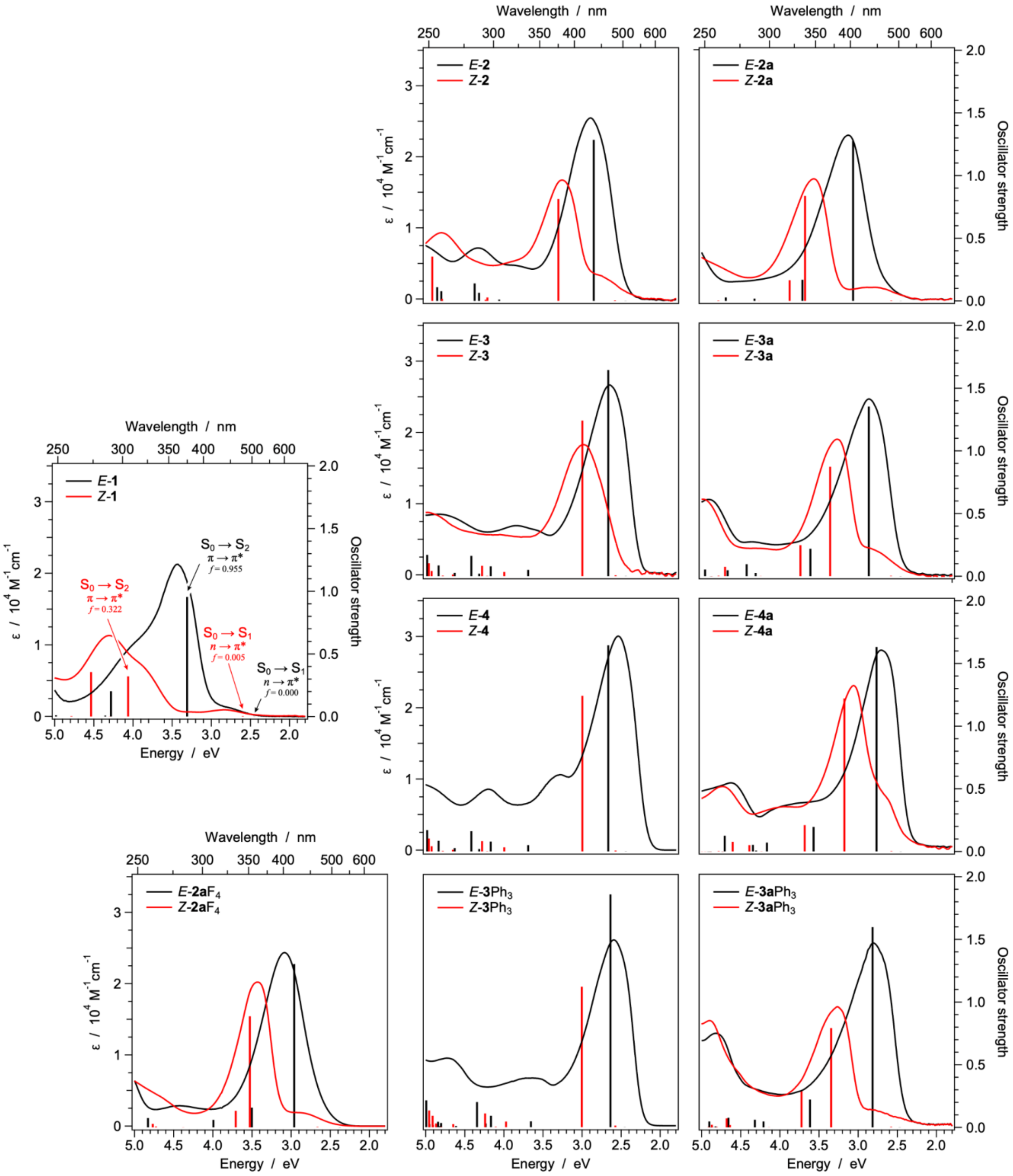

Figure S18. Absorption spectra of *Z*-isomers of investigated molecules (calculated according to Fischer method) and experimental absorption spectra of *E*-isomers in aerated room-temperature dichloromethane solution (as a function of their molar absorption coefficient, ε), compared to the vertical electronic excitations computed at the TD-ωB97M-D4/def2-TZVP level of theory in the same solvent (using CPCM), starting from the absolute minimum of each *E*- or *Z*-isomer. To allow a better comparison with the experimental spectroscopic data, TD-DFT transition energies are red-shifted by 0.6 eV.

Table S3. Calculated NTOs couples describing the lowest two singlet excitations (the n–π* and π–π* transitions) for the investigated molecules in CH_2Cl_2 at the CPCM-STEOM-DLPNO-CCSD/def2-TZVP(-f) level, using the most stable S_0 minimum-energy geometry obtained by DFT optimizations for both the *E*- and *Z*-conformers (see Experimental Section for details). The λ value is the natural transition orbital eigenvalue associated with each NTOs couple; orbital isovalue: 0.04 $e^{-1/2}$ $bohr^{-3/2}$

		Transition energy [eV (nm)]	Oscillator strength	NTO couple: hole	→ electron	λ
(*E*)-**1**	$S_0 \rightarrow S_1$ [n–π*]	2.74 (453)	0.000	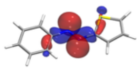	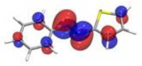	99.7 %
	$S_0 \rightarrow S_2$ [π–π*]	3.63 (342)	0.532	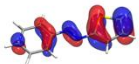	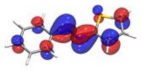	95.3%
(Z)-**1**	$S_0 \rightarrow S_1$ [n–π*]	2.87 (432)	0.002	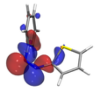	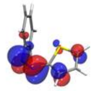	99.6%
	$S_0 \rightarrow S_2$ [π–π*]	4.12 (301)	0.112	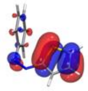	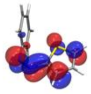	91.3%
(*E*)-**2**	$S_0 \rightarrow S_1$ [n–π*]	2.70 (460)	0.003	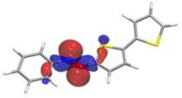	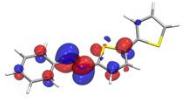	99.8%
	$S_0 \rightarrow S_2$ [π–π*]	3.13 (396)	0.779	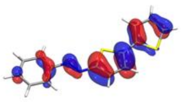	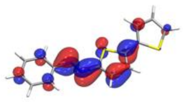	94.9%
(Z)-**2**	$S_0 \rightarrow S_1$ [n–π*]	2.80 (443)	0.002	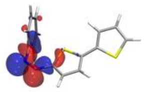	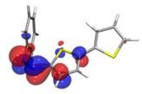	99.6%
	$S_0 \rightarrow S_2$ [π–π*]	3.54 (350)	0.481	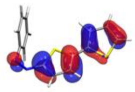	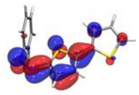	93.2%

(*E*)-**3**	$S_0 \rightarrow S_1$ [n–π*]	2.69 (461)	0.000	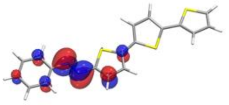	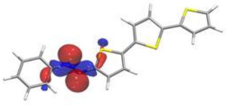	99.7%
	$S_0 \rightarrow S_2$ [π–π*]	2.93 (423)	0.995	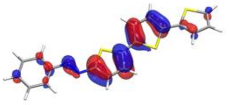	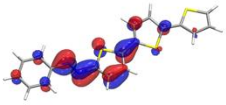	93.0%
(Z)-**3**	$S_0 \rightarrow S_1$ [n–π*]	2.80 (443)	0.002	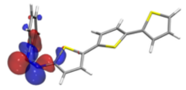	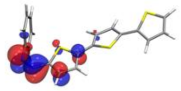	99.6%
	$S_0 \rightarrow S_2$ [π–π*]	3.27 (380)	0.770	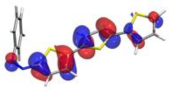	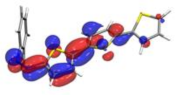	91.7%
(*E*)-**4**	$S_0 \rightarrow S_1$ [n–π*]	2.62 (472)	0.004	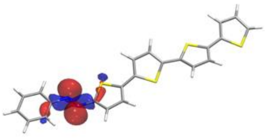	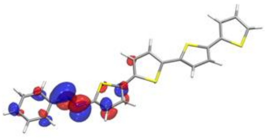	99.8%
	$S_0 \rightarrow S_2$ [π–π*]	2.87 (433)	1.217	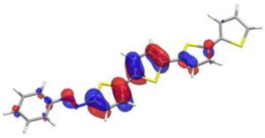	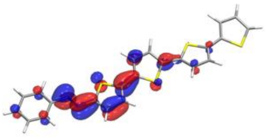	89.2%
(Z)-**4**	$S_0 \rightarrow S_1$ [n–π*]	2.79 (444)	0.009	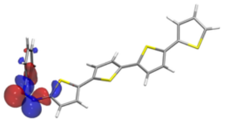	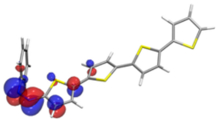	99.3%
	$S_0 \rightarrow S_2$ [π–π*]	3.13 (396)	1.040	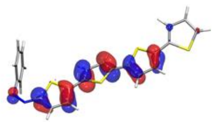	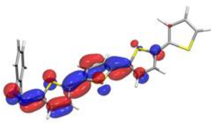	88.6%
(*E*)-**2a**	$S_0 \rightarrow S_1$ [n–π*]	2.70 (459)	0.000	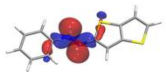	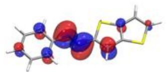	99.6%
	$S_0 \rightarrow S_2$ [π–π*]	3.31 (375)	0.701	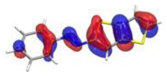	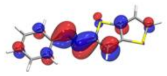	95.7%

(Z)-**2a**	$S_0 \rightarrow S_1$ [n–π*]	2.83 (439)	0.002	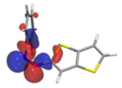	99.7%	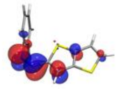
	$S_0 \rightarrow S_2$ [π–π*]	3.80 (326)	0.191	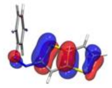	93.5%	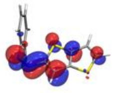
(*E*)-**3a**	$S_0 \rightarrow S_1$ [n–π*]	2.70 (459)	0.000	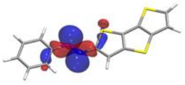	99.8%	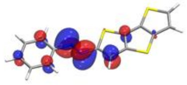
	$S_0 \rightarrow S_2$ [π–π*]	3.03 (410)	0.735	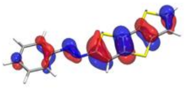	95.4%	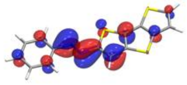
(Z)-**3a**	$S_0 \rightarrow S_1$ [n–π*]	2.84 (442)	0.002	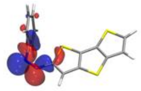	99.7%	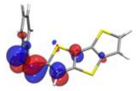
	$S_0 \rightarrow S_2$ [π–π*]	3.47 (357)	0.418	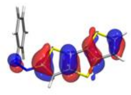	93.6%	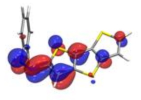
(*E*)-**4a**	$S_0 \rightarrow S_1$ [n–π*]	2.70 (459)	0.000	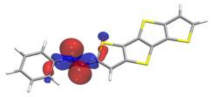	99.8%	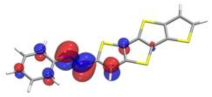
	$S_0 \rightarrow S_2$ [π–π*]	2.90 (428)	0.890	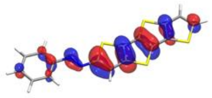	95.0%	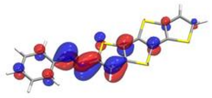
(Z)-**4a**	$S_0 \rightarrow S_1$ [n–π*]	2.79 (444)	0.002	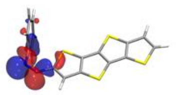	99.7%	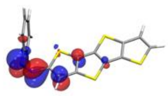
	$S_0 \rightarrow S_2$ [π–π*]	3.27 (379)	0.660	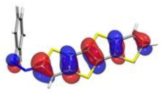	93.4%	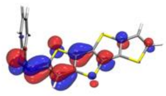

(*E*)-**2a**F_4	$S_0 \rightarrow S_1$ [n–π*]	2.58 (481)	0.000	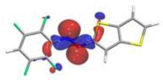	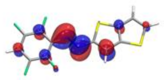	99.8%
	$S_0 \rightarrow S_2$ [π–π*]	3.06 (405)	0.664	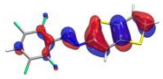	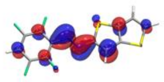	95.9%
(Z)-**2a**F_4	$S_0 \rightarrow S_1$ [n–π*]	2.90 (428)	0.002	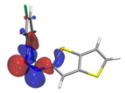	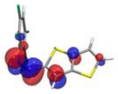	99.7%
	$S_0 \rightarrow S_2$ [π–π*]	3.60 (345)	0.322	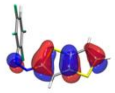	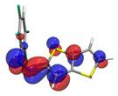	92.8%

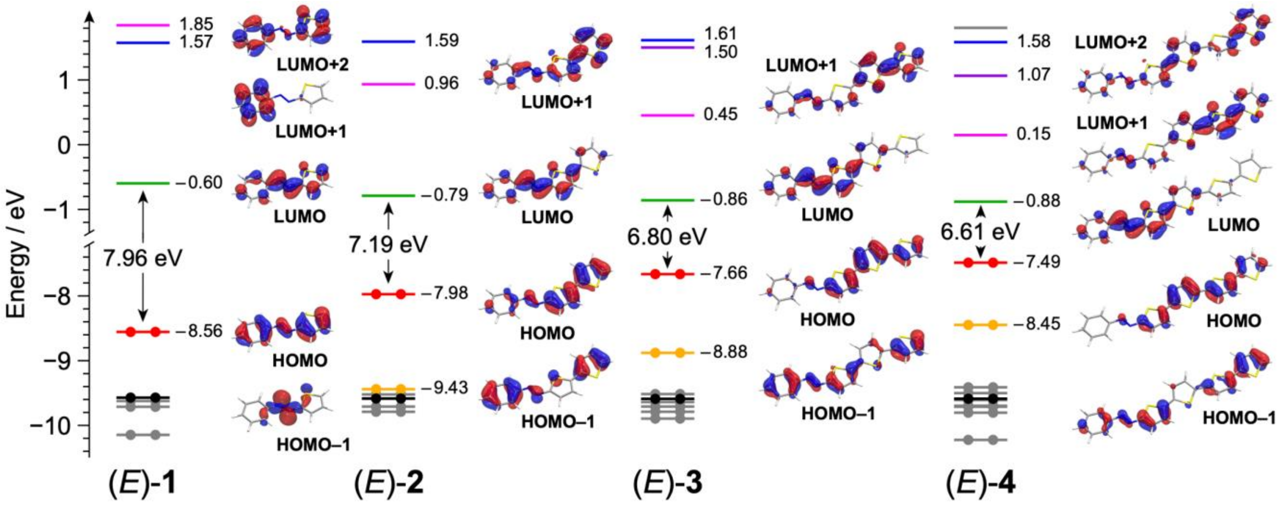

Figure S19. Energy diagram comparing the frontier molecular orbitals of the most stable *E* isomers of **1**–**4** in dichloromethane (isovalue = 0.04 $e^{1/2}$ $bohr^{-3/2}$). Orbitals with similar topologies are reported with the same color.

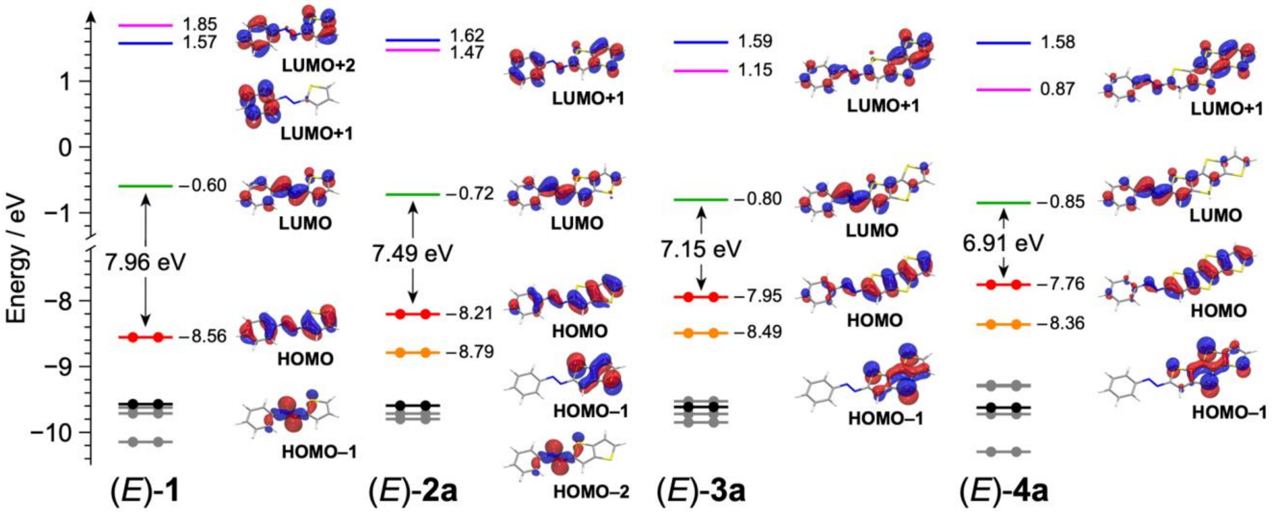

Figure S20. Energy diagram and frontier molecular orbitals of the most stable *E* isomer of **2a**–**4a** in dichloromethane (isovalue = 0.04 $e^{1/2}$ $bohr^{-3/2}$). Orbitals with similar topologies are reported with the same color. *E*-**1** is also reported for comparison

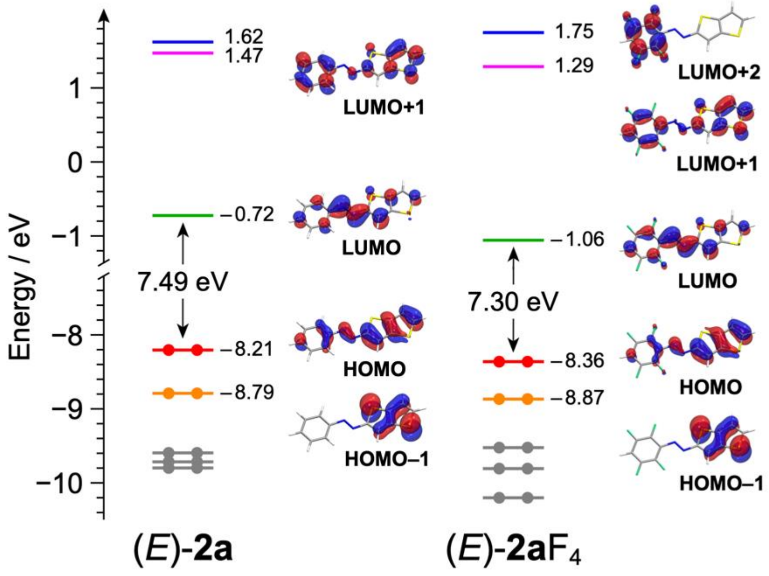

Figure S21. Energy diagram comparing the frontier molecular orbitals of *E*-**2a** and *E*-**2a**F_4 in dichloromethane (isovalue = 0.04 $e^{1/2}$ $bohr^{-3/2}$) to assess the electronic perturbation caused by the presence of the fluorine substituents on the phenyl ring of **2a**; a significant LUMO stabilization is observed.

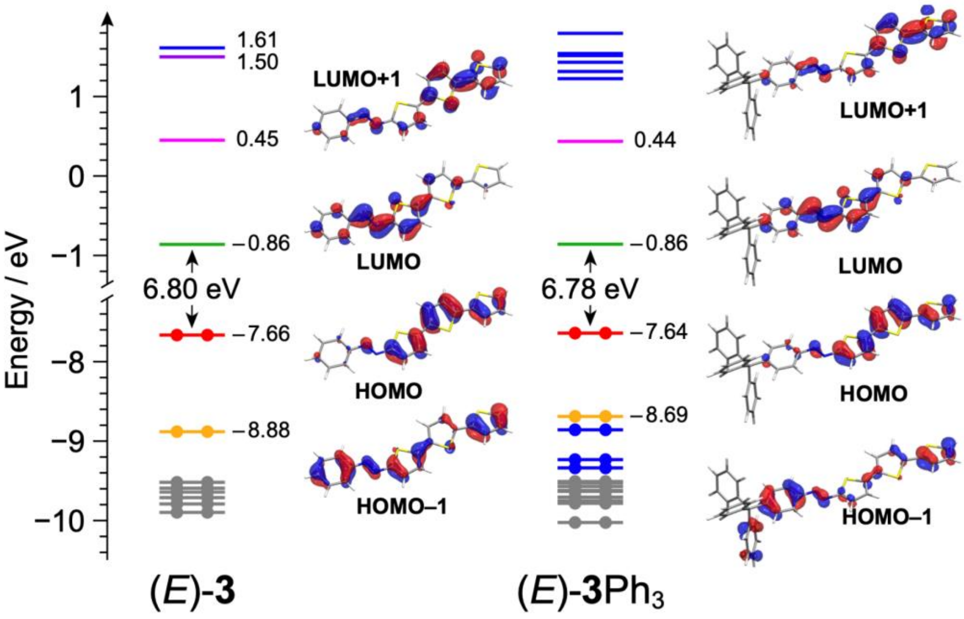

Figure S22. Energy diagrams comparing the frontier molecular orbitals of the most stable conformers of: a) *E* isomers of **3** and **3**Ph_3, and b) the *E*- and *Z*-isomers of **3** and **3**Ph_3; dichloromethane (isovalue = 0.04 $e^{1/2}$ $bohr^{-3/2}$).

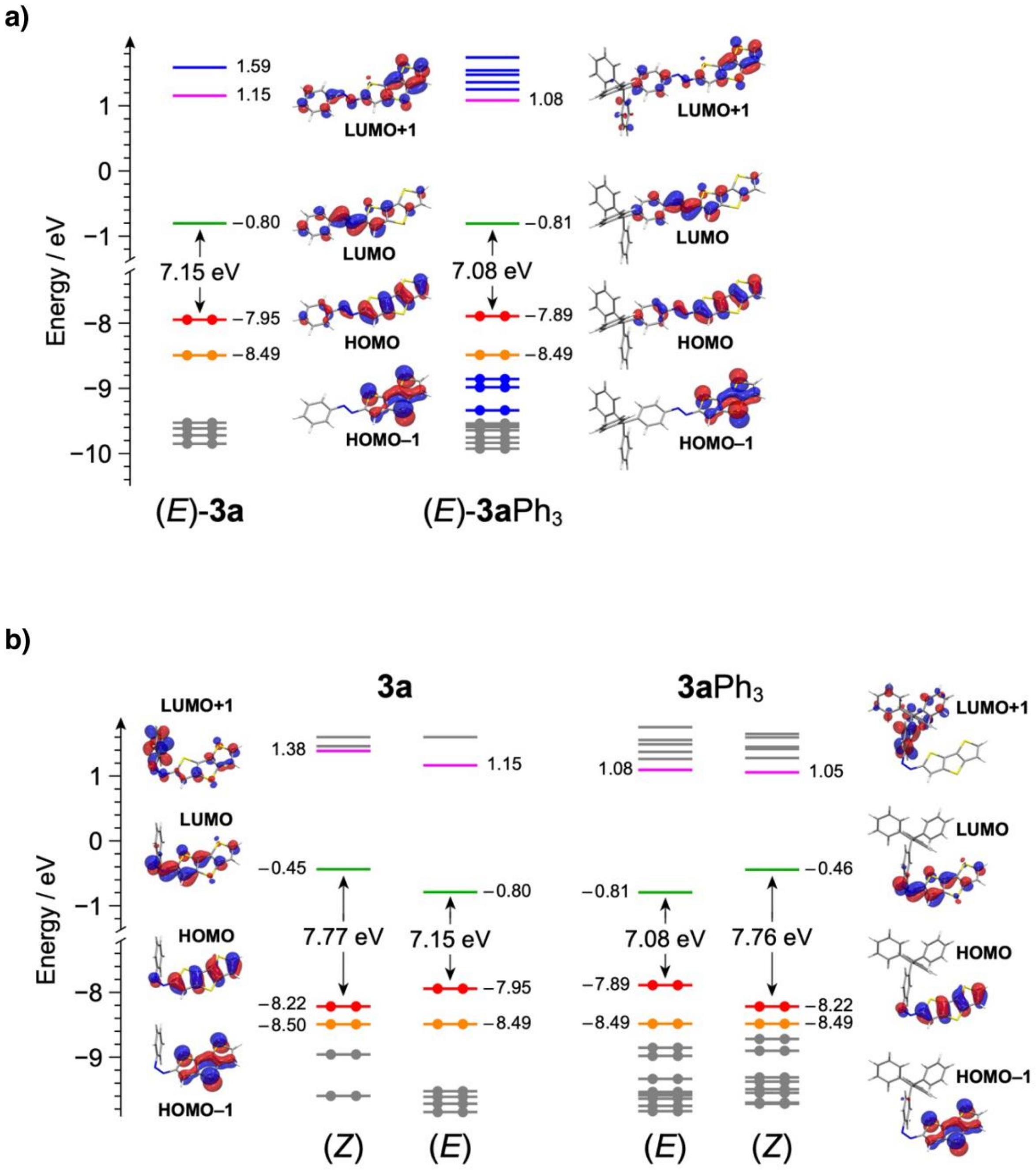

Figure S23. Energy diagrams comparing the frontier molecular orbitals of the most stable conformers of: a) *E* isomers of **3a** and **3a**Ph_3, and b) the *E*- and *Z*-isomers of **3a** and **3a**Ph_3; dichloromethane (isovalue = 0.04 $e^{1/2}$ $bohr^{-3/2}$).

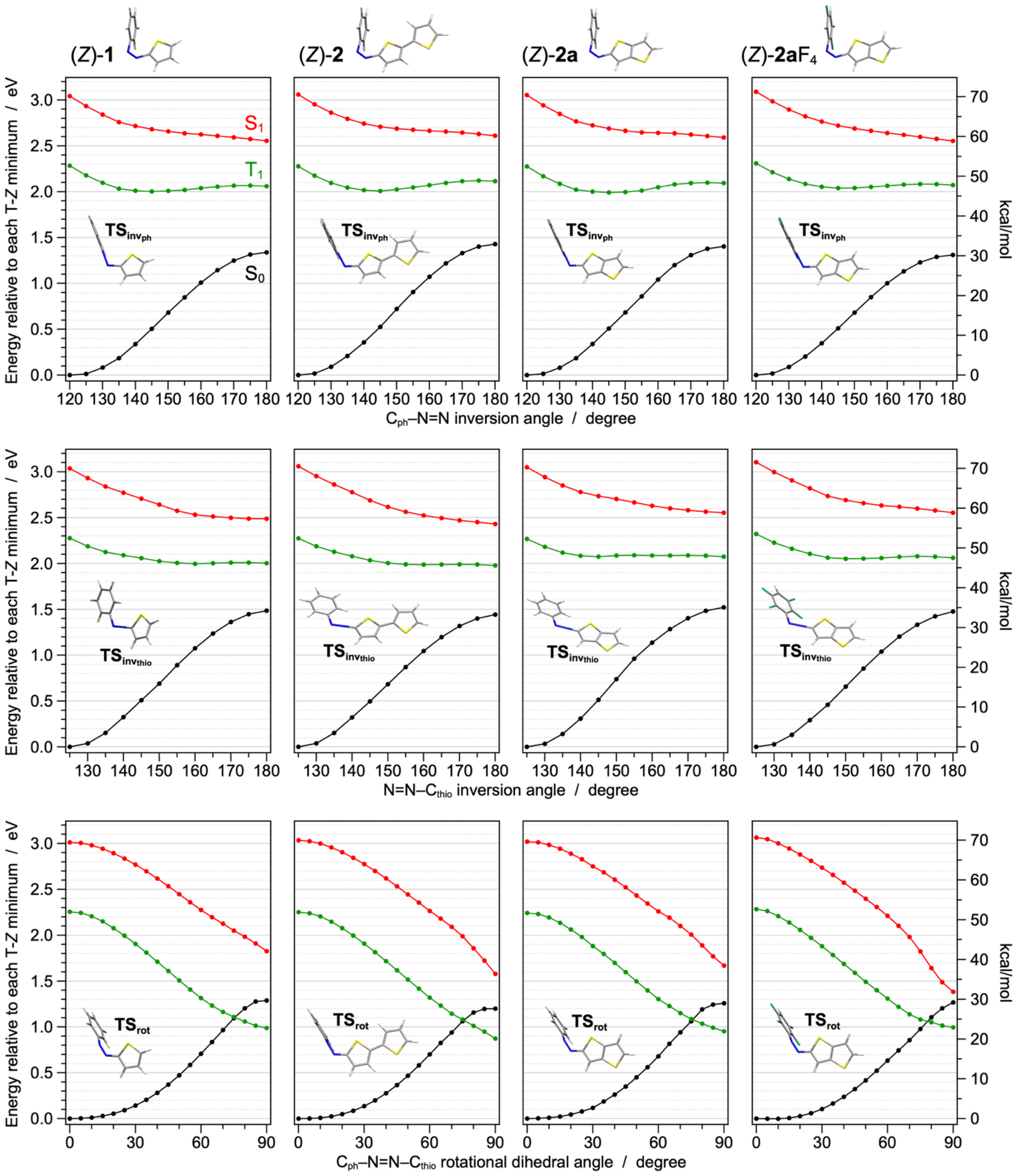

Figure S24. Potential-energy profiles (at the NEVPT2/SA3-CASSCF(14,12)/def2-SVP level in dichloromethane) of S_0 (black), S_1 (red) and T_1 (green) along the three investigated $Z \rightarrow E$ isomerization pathways: phenyl-side inversion (top), thiophene-side inversion (middle) and rotation along the azo bond (bottom). Only the path from the T-shaped *Z*-minimum (on the left side of every graph) to each transition state (TS, right side) was explored. The geometry of each minimum and TS is also reported for all investigated molecules.

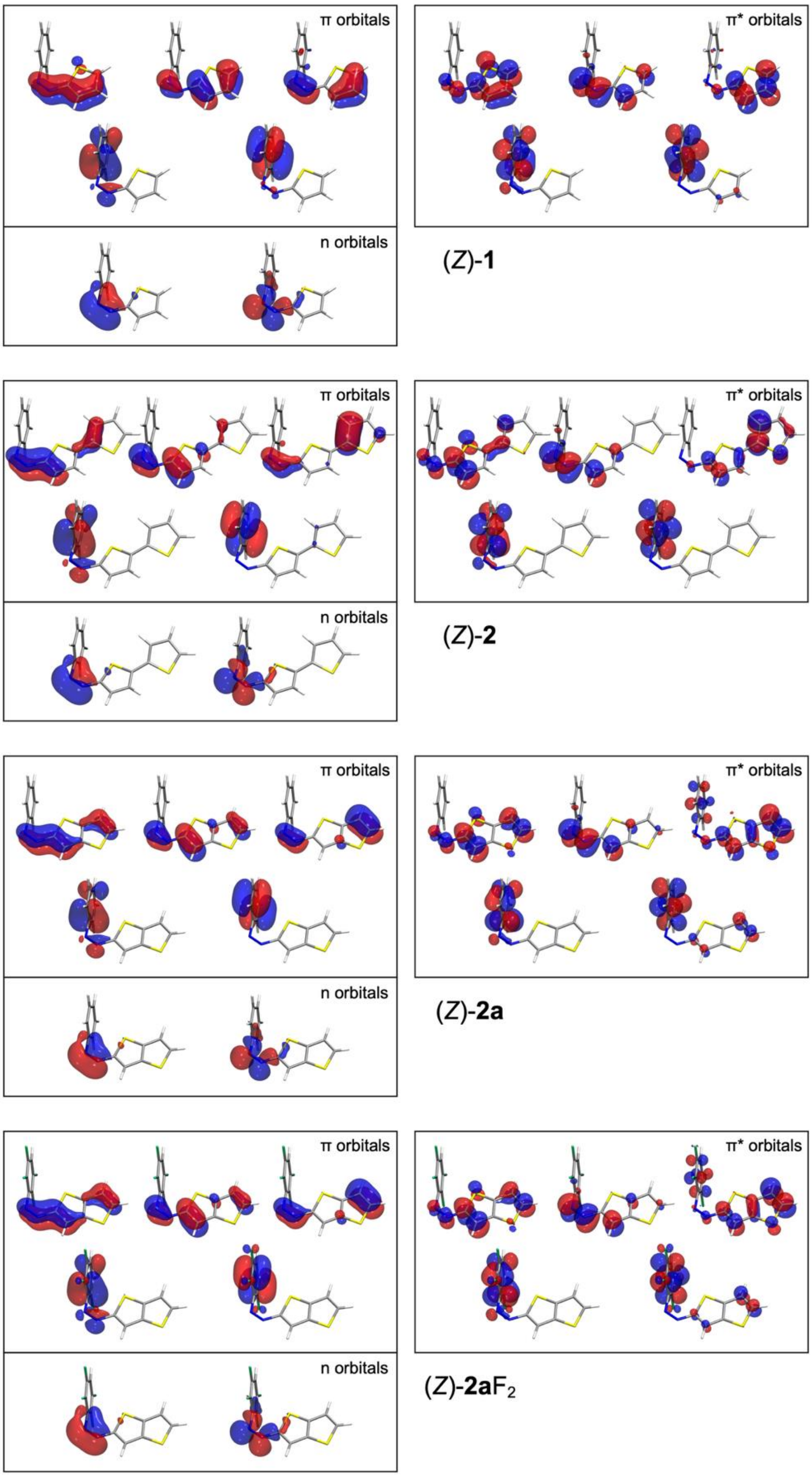

Figure S25. Molecular orbitals of the active spaces selected for **1**, **2**, **2a** and **2a**F_4 (here depicted in their *Z*-isomer minimum) to be used in the NEVPT2/SA3-CASSCF(14,12)/def2-SVP calculations in dichloromethane (using CPCM). Orbital isovalues: 0.04 $e^{1/2}$ $bohr^{-3/2}$.

Table S4. Energy of the transition states relative to the ground-state T-shaped *Z*-minimum of **1**, **2**, **2a** and **2a**F_4, calculated at the NEVPT2/SA3-CASSCF(14,12)/def2-SVP level in dichloromethane (using CPCM), starting from DFT-optimized geometries (see Experimental Section for further details).

TS energy relative to S_0 T-shaped *Z*-minimum [eV]

Compound	***Phenyl-side inversion***	***Thiophene-side inversion***	***C_{ph}–N=N–C_{thio} rotation***
**1**	1.338	1.485	1.287
**2**	1.428	1.442	1.198
**2a**	1.402	1.521	1.259
**2a**F_4	1.310	1.475	1.268

CASSCF/NEVPT2 calculations (see **Figures S24** and **S25**) show that thermal *Z* → *E* back-isomerization in azothiophenes occurs predominantly through a rotational pathway (*i.e.*, torsion around the N=N azo bond), and not exclusively *via* inversion, as previously reported for this class of compounds. For the investigated subset of derivatives (*i.e.*, **1**, **2**, **2a** and **2a**F_4) the computed rotational barriers are consistently lower than the inversion ones (**Table S4**). This theoretical prediction is supported by experiment: the *Z* isomer half-life decreases markedly with the number of thiophene units, particularly when the rings are α-linked ($t_{1/2}$ = 5332, 433 and 81 seconds for **1**, **2a** and **2**, respectively, see **Table S7**). Consistently, **CASSCF/NEVPT2** rotational barriers decline from 29.7 in **1**, to 29.0 in **2a**, and 27.6 kcal/mol in **2** (see **Table S4**), whereas the inversion pathways cannot account for the experimentally observed trend. This result highlights that single-reference DFT methods fail to describe the torsional transition state of azobenzenes and related systems, yielding qualitatively wrong potential energy profiles, and that a multireference treatment is required for an accurate mechanistic description of the *Z* → *E* back-isomerization, not only in azobenzene derivatives, but also in heteroazo systems.

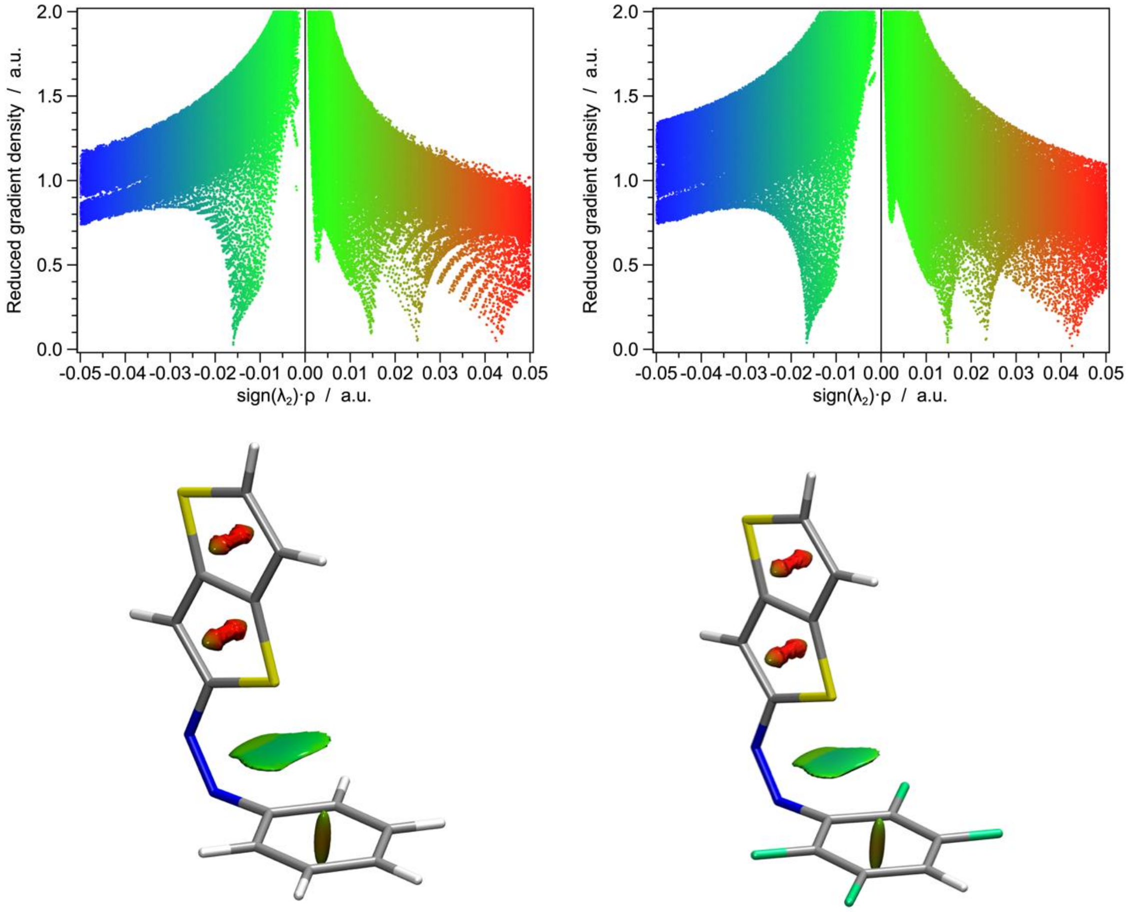

Figure S26. Non-covalent-interaction (NCI) scatter plots at the ωB97M-D4/def2-TZVP level of theory in dichloromethane, using CPCM, for the T-shaped Z-isomers of **2a** (top left) and **2a**F_4 (top right). The NCI isosurfaces, colored according to sign(λ_2)·ρ, are also reported at the bottom (RDG isosurface = 0.5 a.u.), showing the presence of sulfur lone-pair···π interaction.

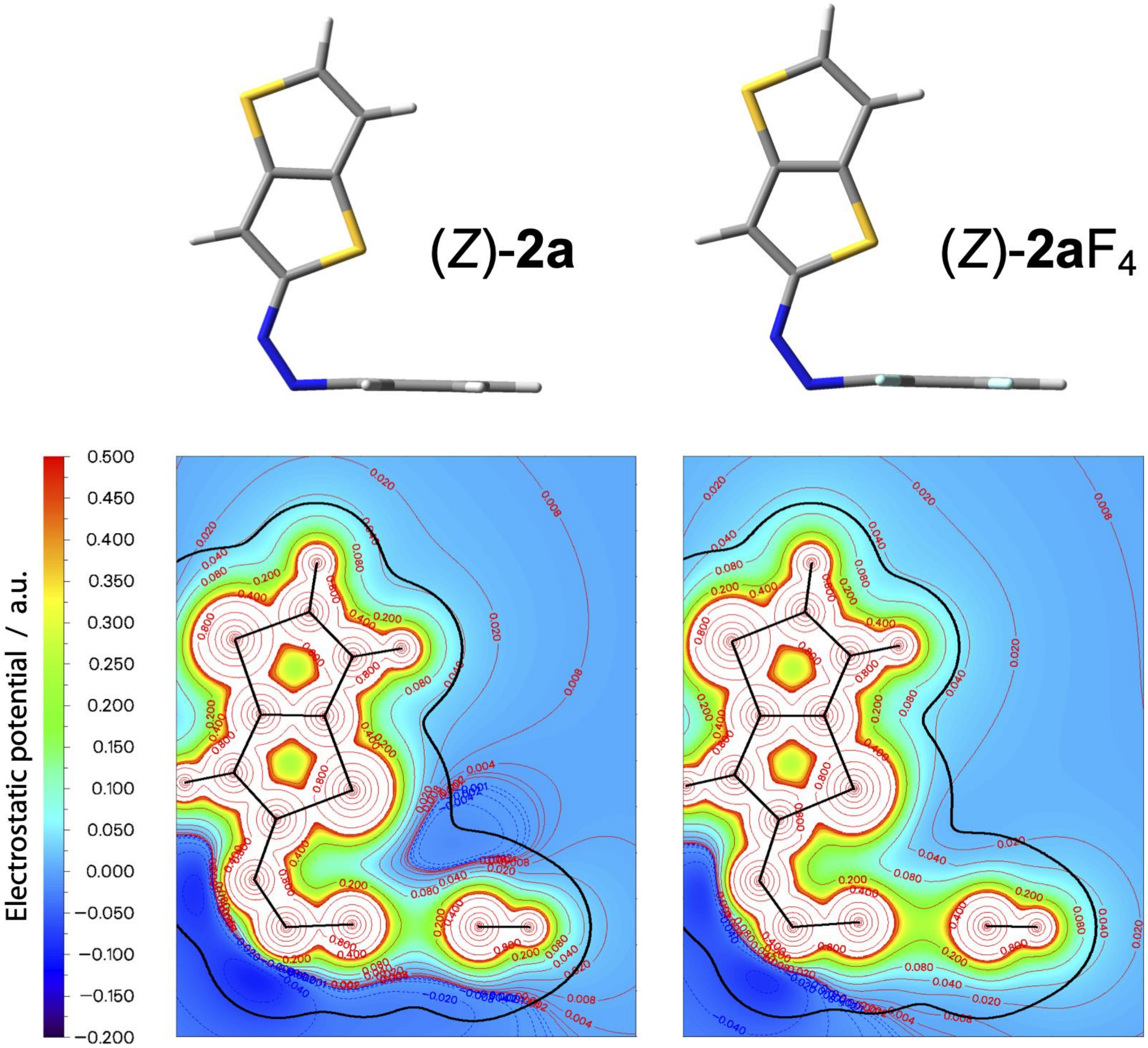

Figure S27. 2D plots of the electrostatic potential (ESP) mapped on the plane of symmetry of the T-shaped *Z*-isomers of **2a** (left) and **2**aF_4 (right). The blue regions indicate electron-rich zones (negative ESP), as in the case of the azo lone pairs or the π-electron cloud under and above the plane of the unsubstituted phenyl ring in *Z*-**2a**.

VI. Cyclic voltammetry measurements (CVs)

Table S5. Redox potentials of the investigated compounds, energy levels of HOMO, LUMO, and comparison of the electrochemical and optical energy gap

Compound	$E^{1/2}_{ox}$ [a] [*V* vs. SCE]	$E°_{rid}$ [*V* vs. SCE]	***HOMO*** [eV]	***LUMO*** [eV]	E_g^{el} [eV]	E_g^{opt} [eV]
E-**2**	1.31	-1.11	5.99	3.57	2.42	2.90 (427 nm)
E-**3**	1.05	-1.09	5.73	3.59	2.14	2.66 (466nm)
E-**4**	0.96	-1.09	5.64	3.59	2.05	2.53 (489 nm)
E-**2a**	1.47	-1.10	6.15	3.58	2.57	3.14 (394 nm)
E-**3a**	1.31	-1.10	5.99	3.58	2.41	2.87 (431nm)
E-**4a**	1.24	-1.09	5.92	3.59	2.33	2.73 (454 nm)
E-**2a**F_4	1.66	-0.80	6.34	3.88	2.46	3.09 (401 nm)
E-**3a**Ph_3	1.29	-1.10	5.97	3.58	2.39	2.76 (448 nm)
E-**3**Ph_3	1.06	-1.09	5.74	3.59	2.15	2.59 (478nm)

[a] Oxidation processes were irreversible and their potentials were estimated as half-wave potentials.

VII. PhotoNMR characterization of 2aF$_4$ and 3aPh$_3$

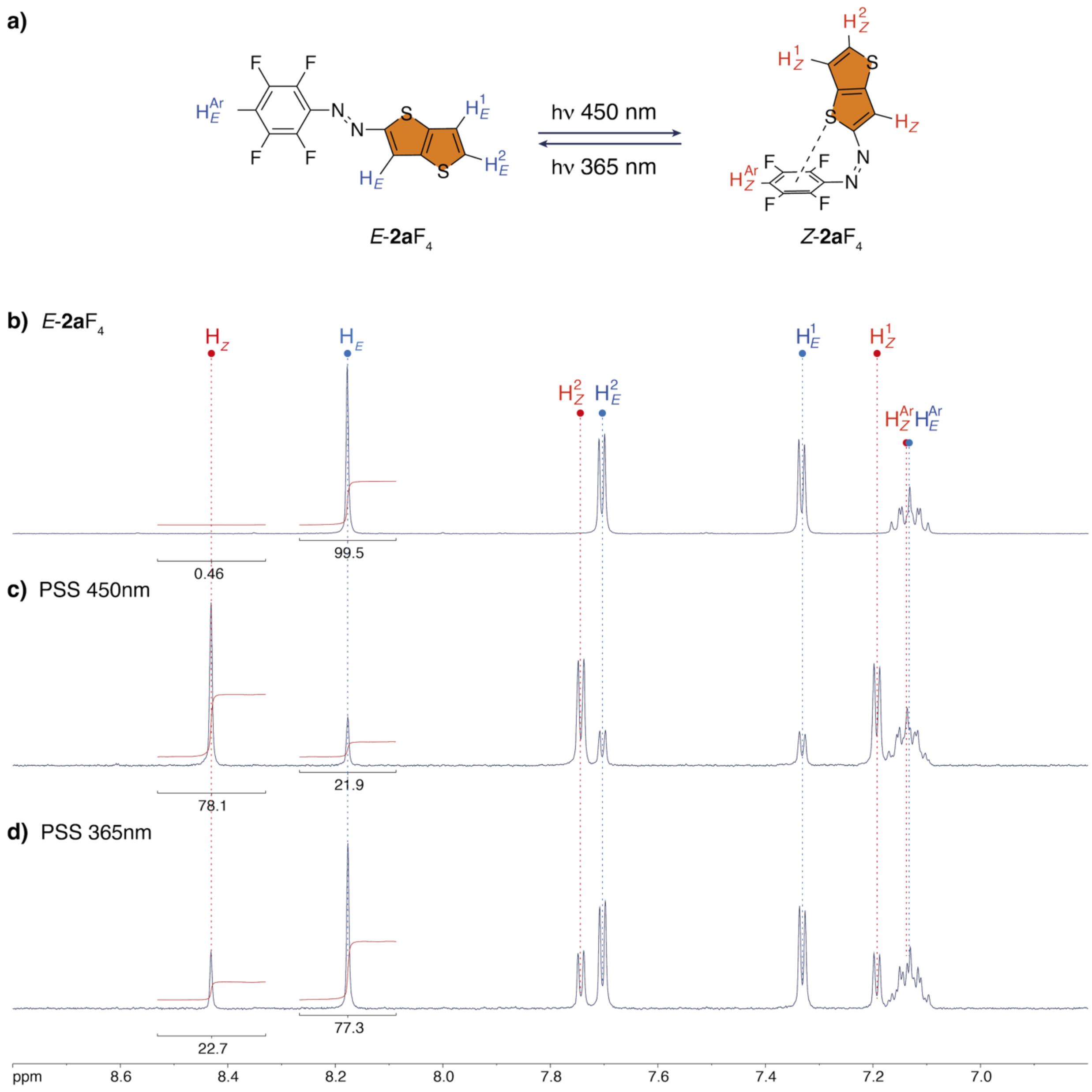

Figure S28. a) Scheme of the *E*/*Z* photoisomerization of **2a**F$_4$. b) ^{1}H NMR spectra (500 MHz, CD_2Cl_2, 298 K) of *E*-**2a**F$_4$. c) ^{1}H NMR spectra (500 MHz, CD_2Cl_2, 298K) of *E*-**2a**F$_4$ after exhaustive irradiation within the NMR probe with a LED light source at *450 nm* (± 20 nm) to the photostationary state. d) ^{1}H NMR spectra (500 MHz, CD_2Cl_2, 298K) of *E*-**2a**F$_4$ after exhaustive irradiation at *365 nm* within the NMR probe with a LED light source (± 20 nm) to the photostationary state. Spectral assignment and relative integrals of diagnostic protons H_E and H_Z for quantification of the stereoisomeric composition are indicated.

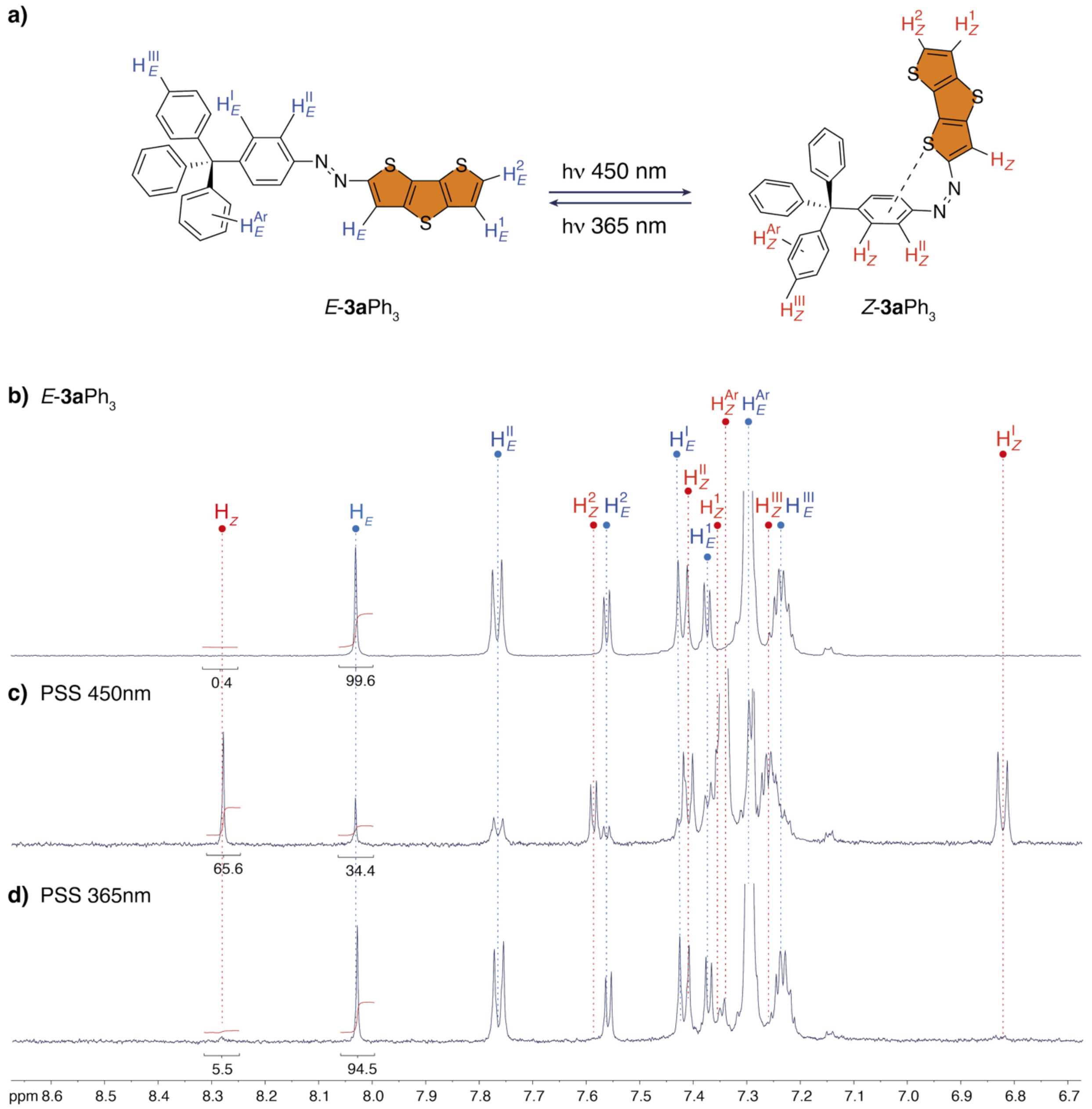

Figure S29. a) Scheme of the *E*/*Z* photoisomerization of **3a**Ph_3. b) ^1H NMR spectra (500 MHz, CD_2Cl_2, 298K) of *E*-**3a**Ph_3. c) ^1H NMR spectra (500 MHz, CD_2Cl_2, 273K) of *E*-**3a**Ph_3 after exhaustive irradiation within the NMR probe with a LED light source at *450 nm* (± 20 nm) to the photostationary state. d) ^1H NMR spectra (500 MHz, CD_2Cl_2, 273K) of *E*-**3a**Ph_3 after exhaustive irradiation within the NMR probe with a LED light source at *365 nm* (± 20 nm) to the photostationary state. Spectral assignment and relative integrals of diagnostic protons H_E and H_Z for quantification of the stereoisomeric composition are indicated.

Table S6. ^{1}H-NMR shielding-tensor data and predicted chemical shifts for the *E*- and *Z*- isomers of compound **2a**F_4, computed at the PBE0/def2-TZVPP in dichloromethane (CPCM) on fully optimized minima at the ωB97M-D4/def2-TZVP level. The variation of chemical shifts and shielding-tensor anisotropy upon $E \rightarrow Z$ isomerization is also reported. An ORTEP-style representation of the proton chemical shielding tensors is also depicted to illustrate the magnitude and orientation of the shielding anisotropy.

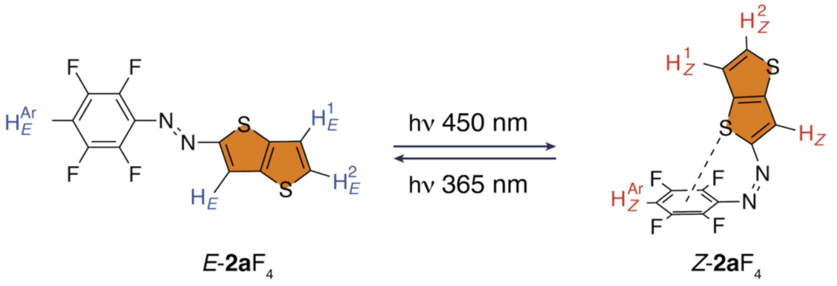

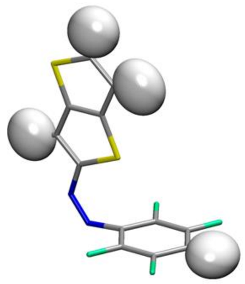

proton	Diagonal elements of the diagonalized total shielding matrix			σ_{iso}	δ (ppm)	Δσ	η	ORCA anisotropy value
				E-**2a**F_4				
H	19.526	23.327	25.834	22.896	**8.659**	**-3.370**	**0.744**	**4.408**
H^1	19.729	25.095	26.640	23.821	**7.734**	**-4.092**	**0.378**	**4.228**
H^2	20.172	24.207	25.752	23.377	**8.178**	**-3.205**	**0.482**	**3.563**
H^{Ar}	20.626	24.415	26.937	23.993	**7.562**	**-3.367**	**0.749**	**4.417**
				Z-**2a**F_4				
H	19.337	23.219	25.148	22.568	**8.987**	**-3.231**	**0.597**	**3.870**
H^1	19.505	25.078	27.363	23.982	**7.573**	**-4.477**	**0.510**	**5.072**
H^2	19.951	24.446	25.646	23.348	**8.207**	**-3.397**	**0.353**	**3.448**
H^{Ar}	20.967	24.750	26.207	23.975	**7.580**	**-3.008**	**0.484**	**3.349**

	$\Delta\sigma_{iso}$	Δδ (ppm)	Δ(Δσ)	Δη	Δanisotropy
H		**+0.328**	**+0.139**	**-0.147**	**-0.537**
H^1		**-0.161**	**-0.385**	**+0.133**	**+0.843**
H^2		**+0.029**	**-0.192**	**-0.129**	**-0.115**
H^{Ar}		**+0.018**	**+0.359**	**-0.265**	**-1.068**

VIII. UV-Vis photochemical characterization in solution

Table S7. UV-vis photophysical and photochemical data of investigated compounds (aerated CH_2Cl_2, RT).

Compound	λ_{max} [nm]	ε [$cm^{-1}M^{-1}$]	$\Phi_{E\rightarrow Z}$ (λ_{irr}, nm)	$\Phi_{Z\rightarrow E}$ (λ_{irr}, nm)	*Z/E* PSS [%] (λ_{irr}, nm)	$k_{Z,\Delta}$ [s^{-1}]	$t_{½}$ [sec]
**1**	362	21200	0.30 (436)	0.39 (436)	44:56 (436)	1.3×10^{-4}	5332
			0.26 (365)	< 0.1(365)	97:3 (365)		
**2**	427	25300	0.44 (436)	0.54 (436) [a]	64:36 (436)	8.5×10^{-3}	81
			0.61 (405)	0.99 (405) [a]	34:66 (405)		
			0.42 (365)	0.99 (365) [a]	12:88 (365)		
**3**	466	27000	0.36 (436)	0.97 (436) [a]	17:83 (436)	3.8×10^{-2}	18
			0.56 (405)	0.99 (405) [a]	10:90 (405)		
			0.41 (365)	< 0.1 (365) [a]	10:90 (365)		
**3**Ph_3	478	26000	0.35 (436)	0.99 (436) [a]	15:85 (436) [b]	1.9×10^{-2}	36
			0.16 (405)	0.99 (405) [a]	10:90 (405) [b]		
			0.19 (365)	0.99 (365) [a]	10:90 (365) [b]		
**4**	489	30000	0.37 (436)	0.45 (436) [a]	10:90 (436) [b]	4.9×10^{-2}	14
			0.62 (405)	0.99 (405) [a]	10:90 (405) [b]		
			0.57 (365)	0.99 (365) [a]	10:90 (365) [b]		
**2a**	394	23000	0.65 (436)	0.87 (436)	75:25 (436)	1.6×10^{-3}	433
			0.83 (405)	0.99 (405)	88:12 (405)		
			0.61 (365)	0.99 (365)	40:60 (365)		
**3a**	431	25000	0.60 (436)	< 0.1 (436) [a]	67:33 (436)	1.2×10^{-2}	58
			0.83 (405)	0.79 (405) [a]	40:60 (405)		
			0.51 (365)	0.99 (365) [a]	14:86 (365)		
**4a**	454	28000	0.61 (436)	0.85 (436) [a]	35:65 (436)	2.2×10^{-2}	31
			0.71 (405)	0.98 (405) [a]	17:83 (405)		
			0.59 (365)	0.99 (365) [a]	9:91 (365)		
**3a**Ph_3	448	25800	—	—	66:34 (450) [c]	1.2×10^{-2}	58
			0.20 (436)	0.40 (436) [a]	57:43 (436)		
			0.19 (405)	0.61 (405) [a]	25:75 (405)		
			—	—	< 5:95 (365)		
			—	—	< 6:94 (365) [c]		
**2a**F_4	401	24300	—	—	36:64 (580)	4.5×10^{-5}	15403
			—	—	78:22 (450) [c]		
			0.39 (436)	0.99 (436)	76:24 (436)		
			0.36 (405)	0.93 (405)	80:20 (405)		
			0.22 (365)	0.67 (365)	20:80 (365)		
			—	—	23:77 (365) [c]		
			—	—	15:85 (337)		

(a) Given the fast rate of thermal back-isomerization of the *Z*-isomer this value is affected by an error >30%.[4]
(b) Given the uncertainty in the spectrum of the pure *Z* isomer, this value can only be estimated.
(c) Data obtained from NMR experiments using unfiltered broadband (Δλ ≈ ±30 nm) LED light sources.

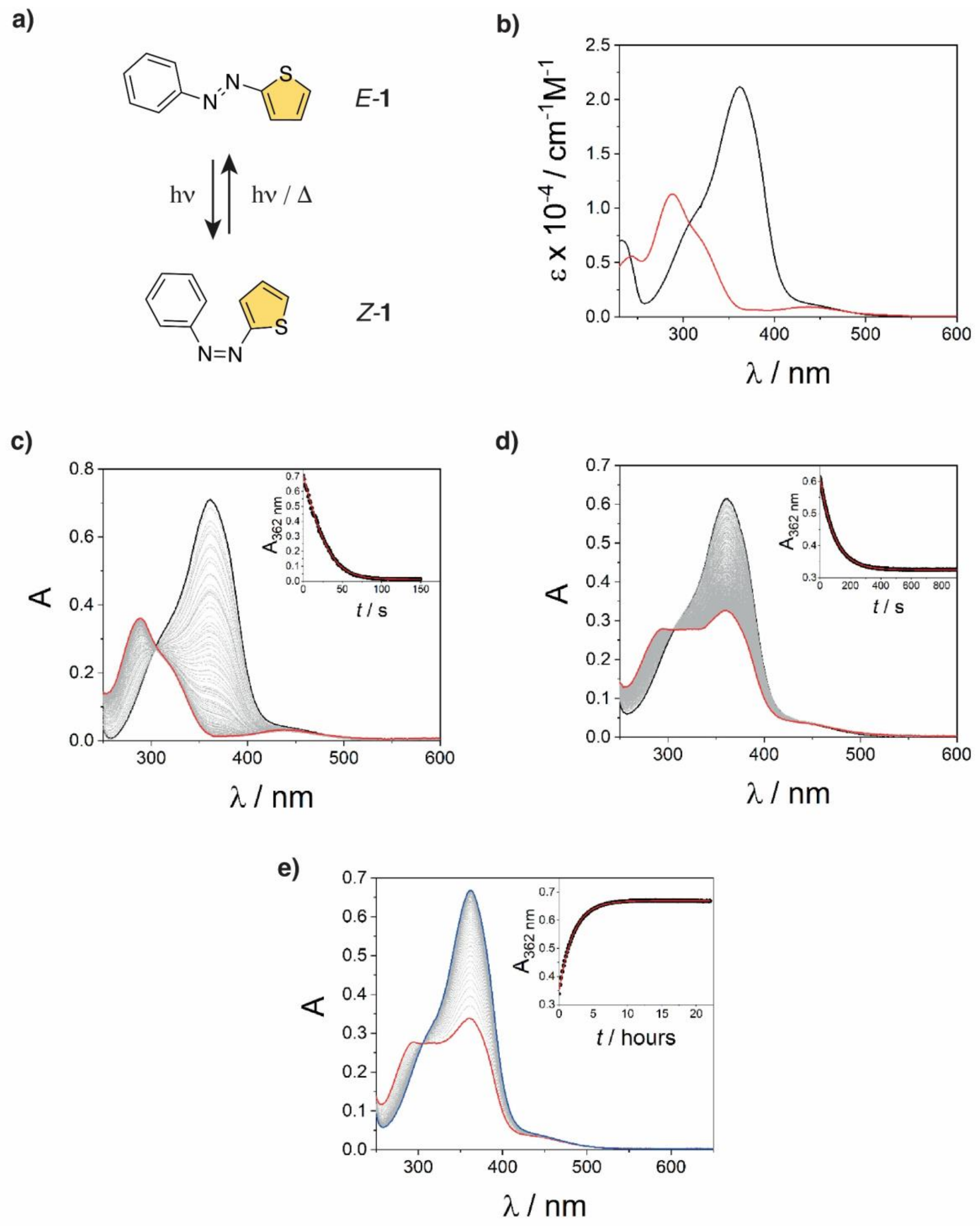

Figure S30. a) Photoisomerization reaction of *E*-**1**. b) Absorption spectra of the *E* (black line) and *Z* (computed by Fischer method, red line) isomers of compound **1** in CH_2Cl_2. c) Absorption variations of a 3.3×10^{-5} M solution of *E*-**1** in CH_2Cl_2 (black line) upon irradiation at 365 nm until PSS is reached (red line); inset: absorption changes at 362 nm (black dots) together with data fitting (red line). d) Absorption variations of a 2.9×10^{-5} M solution of *E*-**1** in CH_2Cl_2 (black line) upon irradiation at 436 nm until PSS is reached (red line); inset: absorption changes at 362 nm (black dots) together with data fitting (red line). e) Thermal isomerization of a 2.9×10^{-5} M solution of *E*-**1** in CH_2Cl_2 after exhaustive irradiation at 436 nm (red line) until total recovery of the *E* isomer (blue line); inset: absorption changes at 362 nm (black dots) together with data fitting (red line).

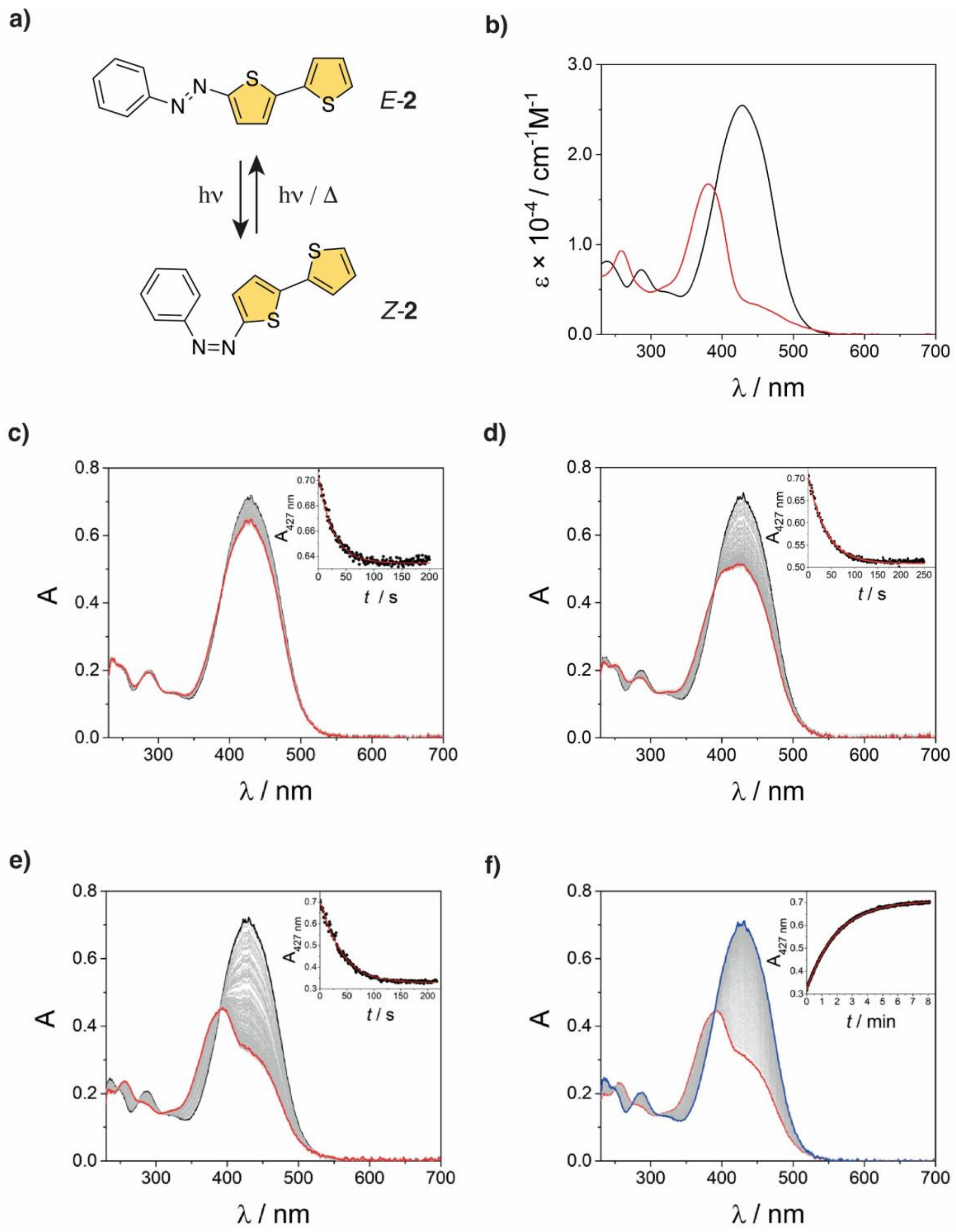

Figure S31. a) Photoisomerization reaction of *E*-**2**. b) Absorption spectra of the *E* (black line) and *Z* (computed by Fischer method, red line) isomers of compound **2** in CH_2Cl_2. c) Absorption variations of a 1.9×10^{-5} M solution of *E*-**2** in CH_2Cl_2 (black line) upon irradiation at 365 nm until PSS is reached (red line); inset: absorption changes at 427 nm (black dots) together with data fitting (red line). d) Absorption variations of a 1.9×10^{-5} M solution of *E*-**2** in CH_2Cl_2 (black line) upon irradiation at 405 nm until PSS is reached (red line); inset: absorption changes at 427 nm (black dots) together with data fitting (red line). e) Absorption variations of a 1.9×10^{-5} M solution of *E*-**2** in CH_2Cl_2 (black line) upon irradiation at 436 nm until PSS is reached (red line); inset: absorption changes at 427 nm (black dots) together with data fitting (red line). f) Thermal isomerization of a 1.9×10^{-5} M solution of *E*-**2** in CH_2Cl_2 after exhaustive irradiation at 436 nm (red line) until total recovery of the *E* isomer (blue line); inset: absorption changes at 427 nm (black dots) together with data fitting (red line).

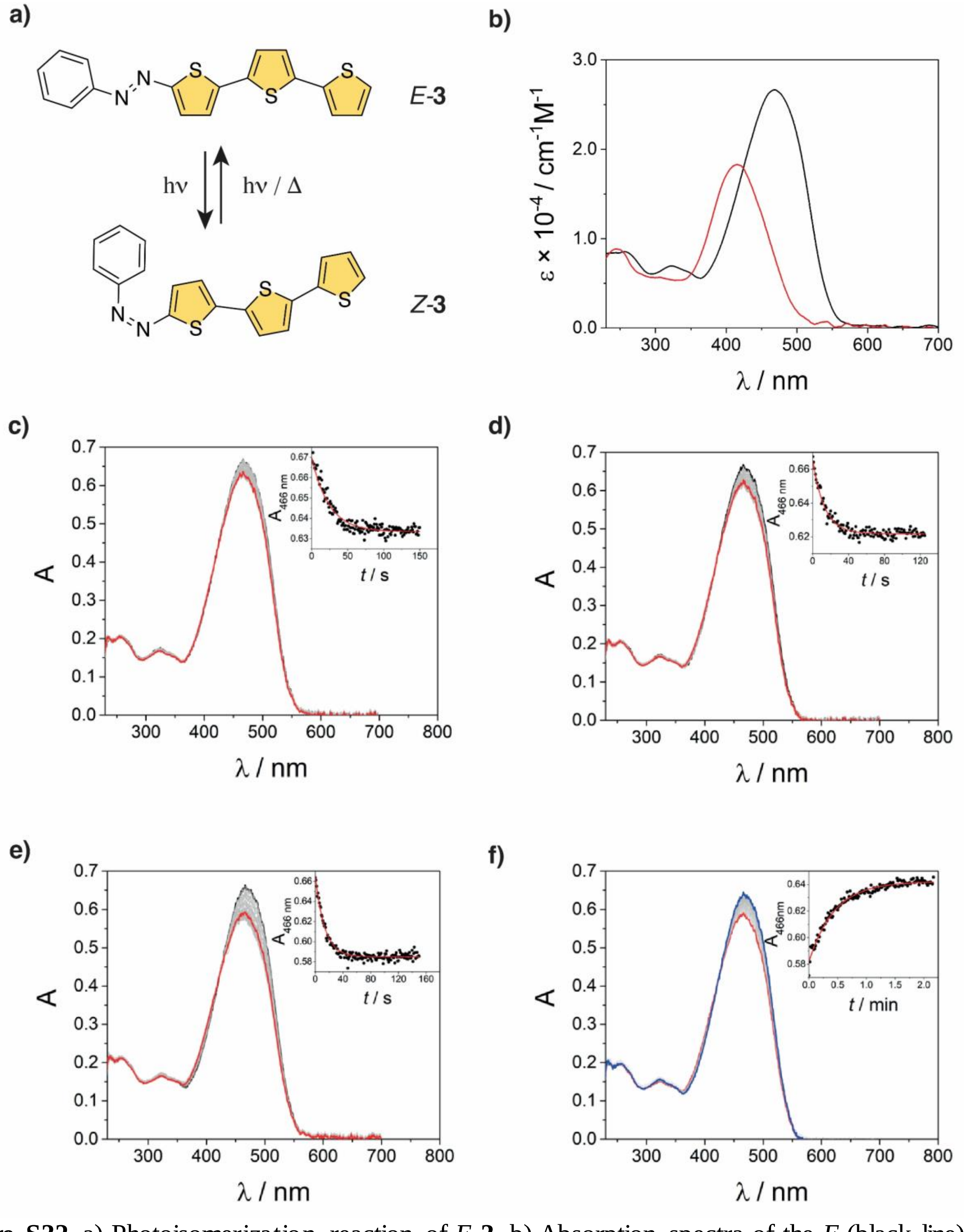

Figure S32. a) Photoisomerization reaction of *E*-**3**. b) Absorption spectra of the *E* (black line) and *Z* (computed by Fischer method, red line) isomers of compound **3** in CH_2Cl_2. c) Absorption variations of a 2.5×10^{-5} M solution of *E*-**3** in CH_2Cl_2 (black line) upon irradiation at 365 nm until PSS is reached (red line); inset: absorption changes at 466 nm (black dots) together with data fitting (red line). d) Absorption variations of a 2.5×10^{-5} M solution of *E*-**3** in CH_2Cl_2 (black line) upon irradiation at 405 nm until PSS is reached (red line); inset: absorption changes at 466 nm (black dots) together with data fitting (red line). e) Absorption variations of a 2.5×10^{-5} M solution of *E*-**3** in CH_2Cl_2 (black line) upon irradiation at 436 nm until PSS is reached (red line); inset: absorption changes at 466 nm (black dots) together with data fitting (red line). f) Thermal isomerization of a 2.5×10^{-5} M solution of *E*-**3** in CH_2Cl_2 after exhaustive irradiation at 436 nm (red line) until total recovery of the *E* isomer (blue line); inset: absorption changes at 466 nm (black dots) together with data fitting (red line).

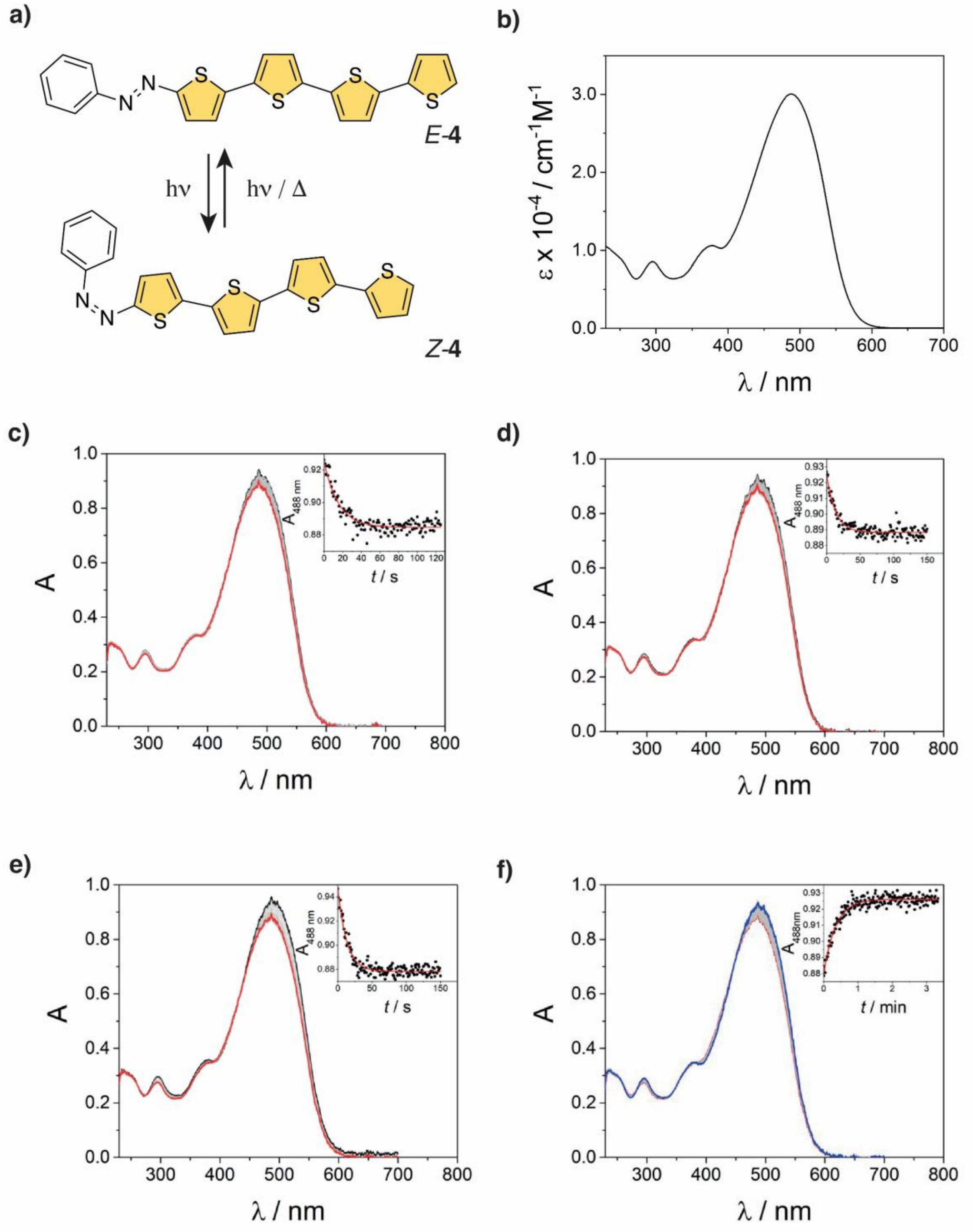

Figure S33. a) Photoisomerization reaction of *E*-**4**. b) Absorption spectrum of the *E* isomer of compound **4** in CH_2Cl_2; the spectrum of its *Z* isomer could not be estimated. c) Absorption variations of a 3.1×10^{-5} M solution of *E*-**4** in CH_2Cl_2 (black line) upon irradiation at 365 nm until PSS is reached (red line); inset: absorption changes at 488 nm (black dots) together with data fitting (red line). d) Absorption variations of a 3.1×10^{-5} M solution of *E*-**4** in CH_2Cl_2 (black line) upon irradiation at 405 nm until PSS is reached (red line); inset: absorption changes at 488 nm (black dots) together with data fitting (red line). e) Absorption variations of a 3.1×10^{-5} M solution of *E*-**4** in CH_2Cl_2 (black line) upon irradiation at 436 nm until PSS is reached (red line); inset: absorption changes at 488 nm (black dots) together with data fitting (red line). f) Thermal isomerization of a 3.1×10^{-5} M solution of *E*-**4** in CH_2Cl_2 after exhaustive irradiation at 436 nm (red line) until total recovery of the *E* isomer (blue line); inset: absorption changes at 488 nm (black dots) together with data fitting (red line).

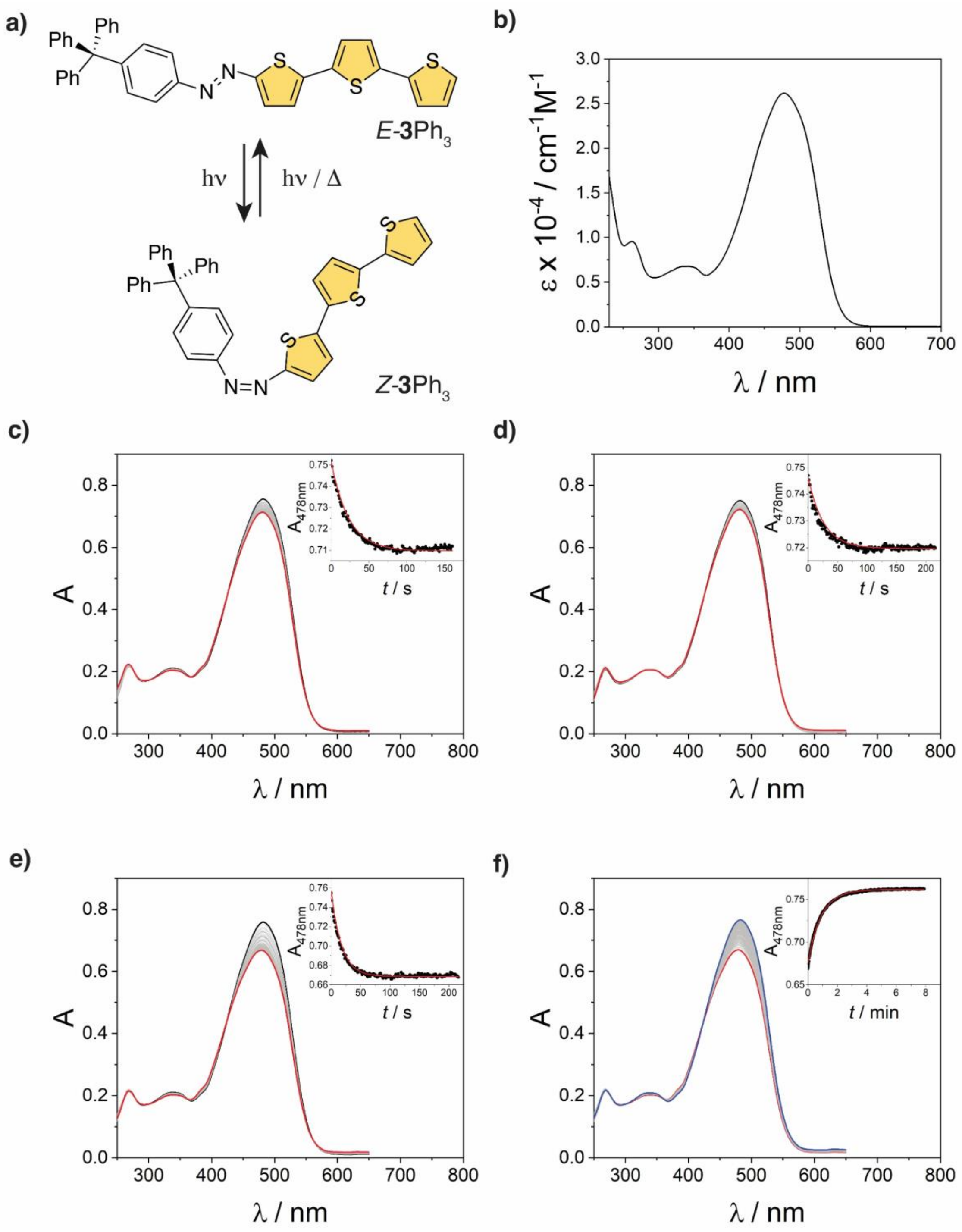

Figure S34. a) Photoisomerization reaction of *E*-**3**Ph_3. b) Absorption spectra of the *E* isomer of compound **3**Ph_3 in CH_2Cl_2; the spectrum of its Z isomer could not be calculated. c) Absorption variations of a 2.9×10^{-5} M solution of *E*-**3**Ph_3 in CH_2Cl_2 (black line) upon irradiation at 365 nm until PSS is reached (red line); inset: absorption changes at 478 nm (black dots) together with data fitting (red line). d) Absorption variations of a 2.9×10^{-5} M solution of *E*-**3**Ph_3 in CH_2Cl_2 (black line) upon irradiation at 405 nm until PSS is reached (red line); inset: absorption changes at 478 nm (black dots) together with data fitting (red line). e) Absorption variations of a 2.9×10^{-5} M solution of *E*-**3**Ph_3 in CH_2Cl_2 (black line) upon irradiation at 436 nm until PSS is reached (red line); inset: absorption changes at 478 nm (black dots) together with data fitting (red line). f) Thermal isomerization of a 2.9×10^{-5} M solution of *E*-**3**Ph_3 in CH_2Cl_2 after exhaustive irradiation at 436 nm (red line) until total recovery of the *E* isomer (blue line); inset: absorption changes at 427 nm (black dots) together with data fitting (red line).

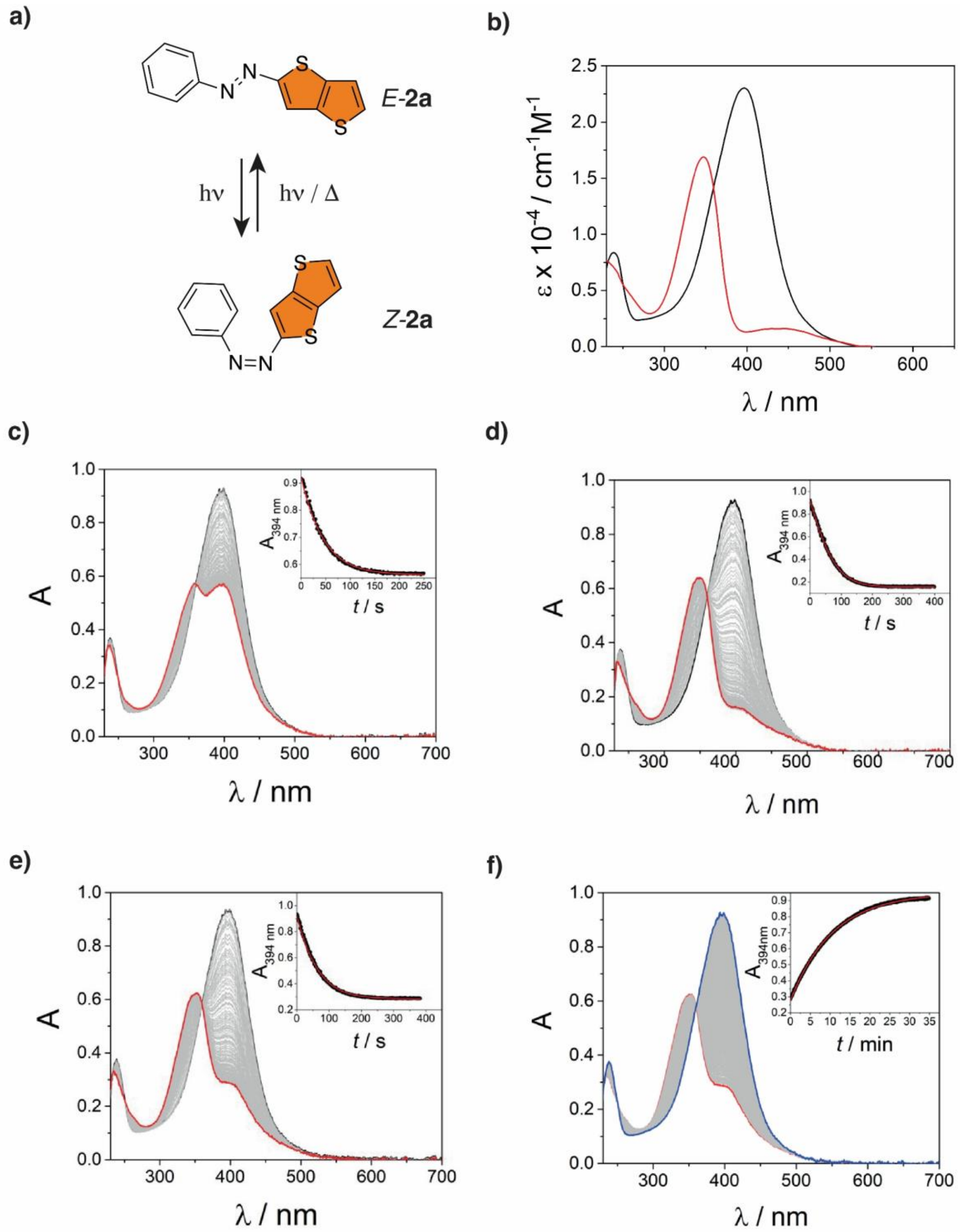

Figure S35. a) Photoisomerization reaction of *E*-**2a**. b) Absorption spectra of the *E* (black line) and *Z* (computed by Fischer method, red line) isomers of compound **2a** in CH_2Cl_2. c) Absorption variations of a 4.0×10^{-5} M solution of *E*-**2a** in CH_2Cl_2 (black line) upon irradiation at 365 nm until PSS is reached (red line); inset: absorption changes at 394 nm (black dots) together with data fitting (red line). d) Absorption variations of a 4.0×10^{-5} M solution of *E*-**2a** in CH_2Cl_2 (black line) upon irradiation at 405 nm until PSS is reached (red line); inset: absorption changes at 394 nm (black dots) together with data fitting (red line). e) Absorption variations of a 4.0×10^{-5} M solution of *E*-**2a** in CH_2Cl_2 (black line) upon irradiation at 436 nm until PSS is reached (red line); inset: absorption changes at 394 nm (black dots) together with data fitting (red line). f) Thermal isomerization of a 4.0×10^{-5} M solution of *E*-**2a** in CH_2Cl_2 after exhaustive irradiation at 436 nm (red line) until total recovery of the *E* isomer (blue line); inset: absorption changes at 394 nm (black dots) together with data fitting (red line).

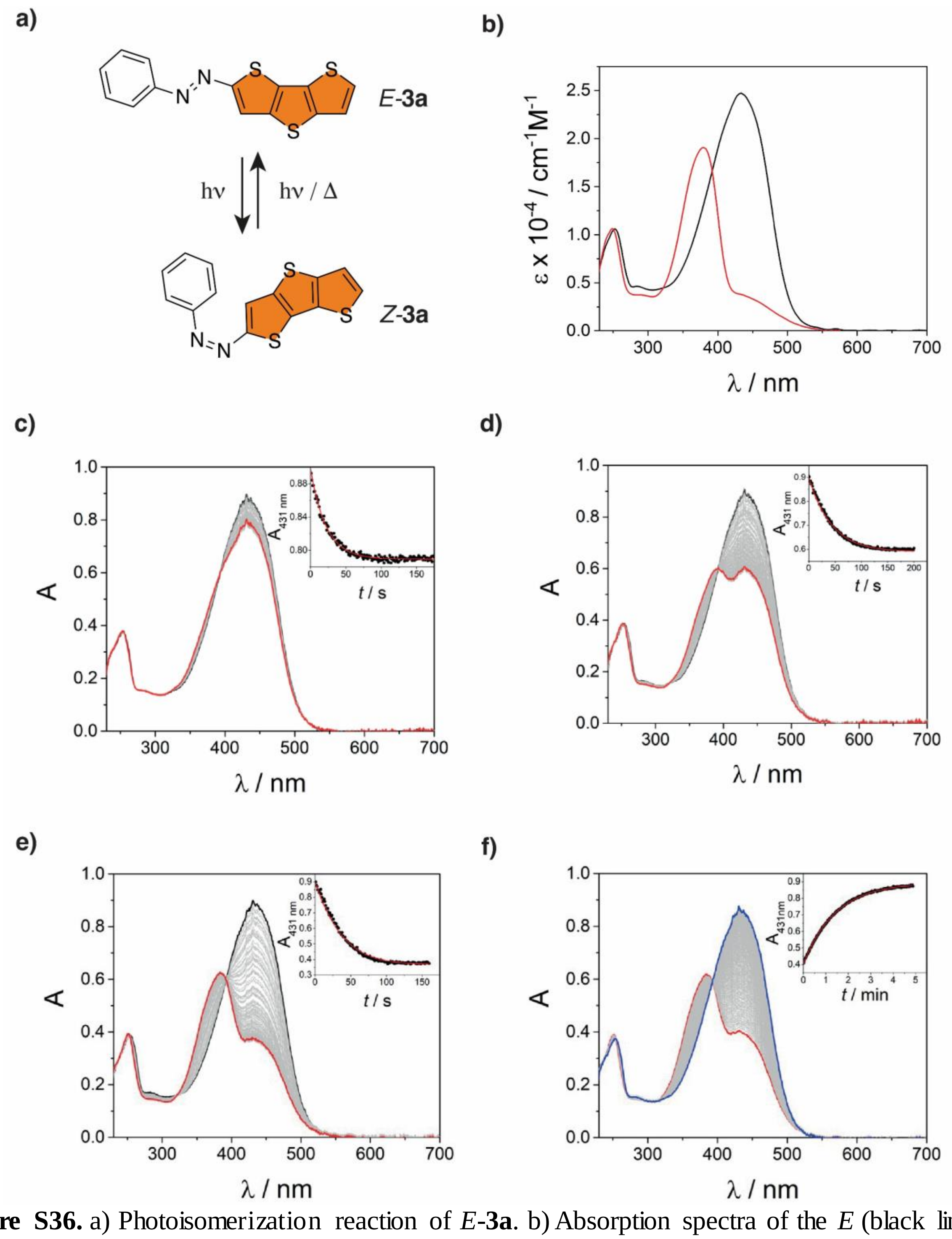

Figure S36. a) Photoisomerization reaction of *E*-**3a**. b) Absorption spectra of the *E* (black line) and *Z* (computed by Fischer method, red line) isomers of compound **3a** in CH_2Cl_2. c) Absorption variations of a 3.6×10^{-5} M solution of *E*-**3a** in CH_2Cl_2 (black line) upon irradiation at 365 nm until PSS is reached (red line); inset: absorption changes at 431 nm (black dots) together with data fitting (red line). d) Absorption variations of a 3.6×10^{-5} M solution of *E*-**3a** in CH_2Cl_2 (black line) upon irradiation at 405 nm until PSS is reached (red line); inset: absorption changes at 431 nm (black dots) together with data fitting (red line). e) Absorption variations of a 3.6×10^{-5} M solution of *E*-**3a** in CH_2Cl_2 (black line) upon irradiation at 436 nm until PSS is reached (red line); inset: absorption changes at 431 nm (black dots) together with data fitting (red line). f) Thermal isomerization of a 3.6×10^{-5} M solution of *E*-**3a** in CH_2Cl_2 after exhaustive irradiation at 436 nm (red line) until total recovery of the *E* isomer (blue line); inset: absorption changes at 431 nm (black dots) together with data fitting (red line).

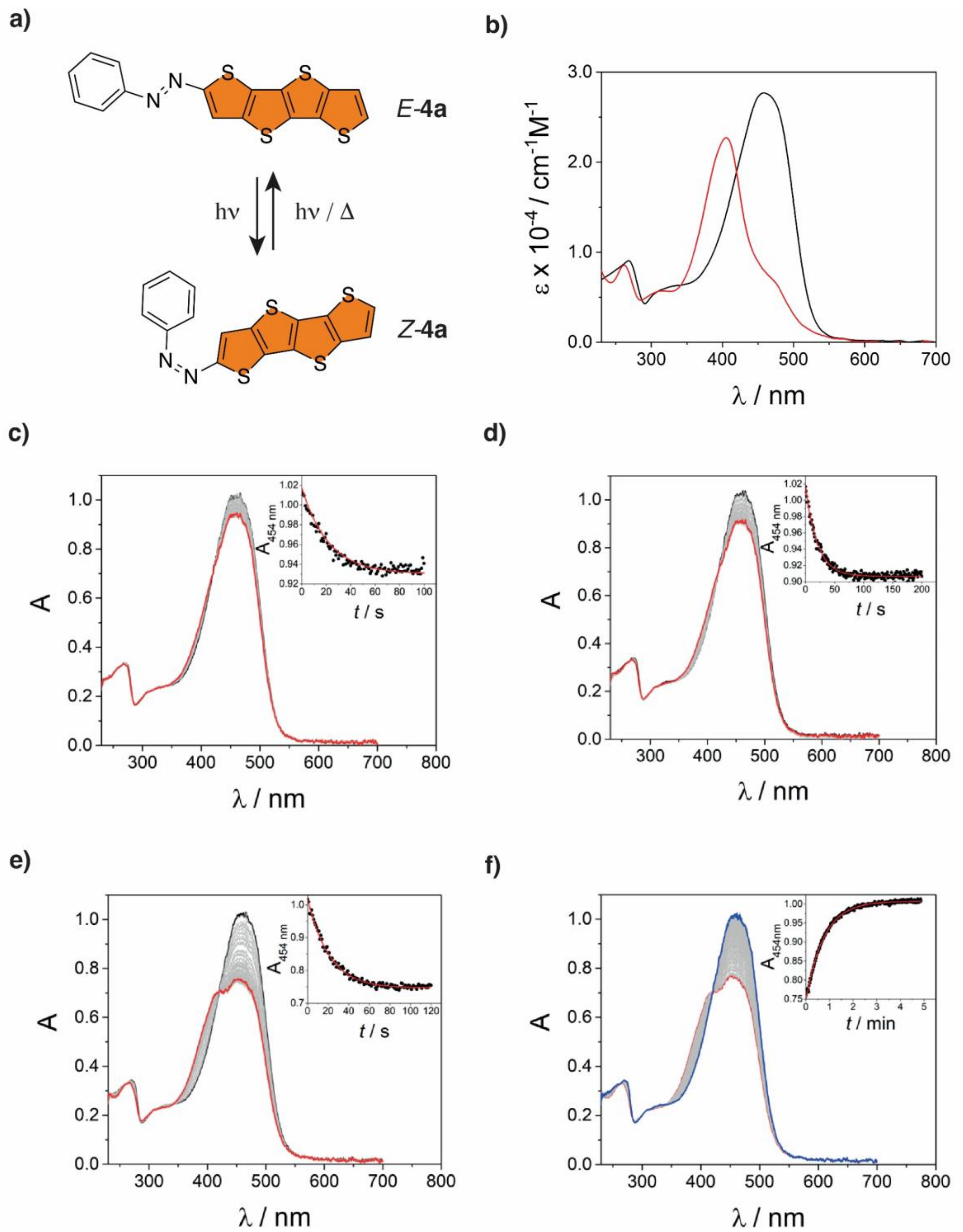

Figure S37. a) Photoisomerization reaction of *E*-**4a**. b) Absorption spectra of the *E* (black line) and *Z* (computed by Fischer method, red line) isomers of compound **4a** in CH_2Cl_2. c) Absorption variations of a 3.6×10^{-5} M solution of *E*-**4a** in CH_2Cl_2 (black line) upon irradiation at 365 nm until PSS is reached (red line); inset: absorption changes at 454 nm (black dots) together with data fitting (red line). d) Absorption variations of a 3.6×10^{-5} M solution of *E*-**4a** in CH_2Cl_2 (black line) upon irradiation at 405 nm until PSS is reached (red line); inset: absorption changes at 454 nm (black dots) together with data fitting (red line). e) Absorption variations of a 3.6×10^{-5} M solution of *E*-**4a** in CH_2Cl_2 (black line) upon irradiation at 436 nm until PSS is reached (red line); inset: absorption changes at 454 nm (black dots) together with data fitting (red line). f) Thermal isomerization of a 3.6×10^{-5} M solution of *E*-**4a** in CH_2Cl_2 after exhaustive irradiation at 436 nm (red line) until total recovery of the *E* isomer (blue line); inset: absorption changes at 454 nm (black dots) together with data fitting (red line).

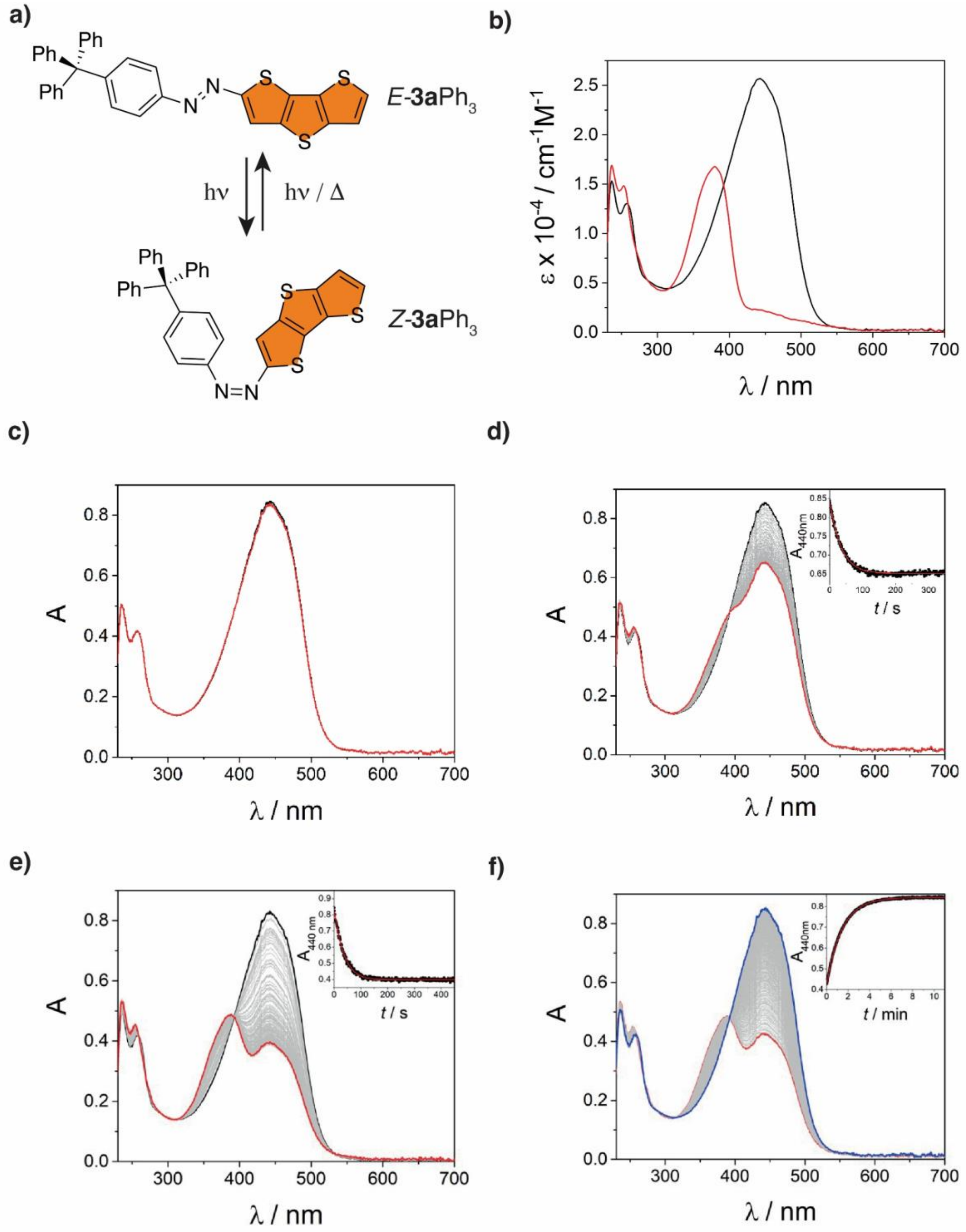

Figure S38. a) Photoisomerization reaction of *E*-**3a**Ph_3. b) Absorption spectra of the *E* (black line) and *Z* (red line) isomers of compound **3a**Ph_3 in CH_2Cl_2. c) Absorption variations of a 3.3×10^{-5} M solution of *E*-**3a**Ph_3 in CH_2Cl_2 (black line) upon irradiation at 365 nm until PSS is reached (red line). d) Absorption variations of a 3.3×10^{-5} M solution of *E*-**3a**Ph_3 in CH_2Cl_2 (black line) upon irradiation at 405 nm until PSS is reached (red line); inset: absorption changes at 440 nm (black dots) together with data fitting (red line). e) Absorption variations of a 3.3×10^{-5} M solution of *E*-**3a**Ph_3 in CH_2Cl_2 (black line) upon irradiation at 436 nm until PSS is reached (red line); inset: absorption changes at 440 nm (black dots) together with data fitting (red line). f) Thermal isomerization of a 3.3×10^{-5} M solution of *E*-**3a**Ph_3 in CH_2Cl_2 after exhaustive irradiation at 436 nm (red line) until total recovery of the *E* isomer (blue line); inset: absorption changes at 440 nm (black dots) together with data fitting (red line).

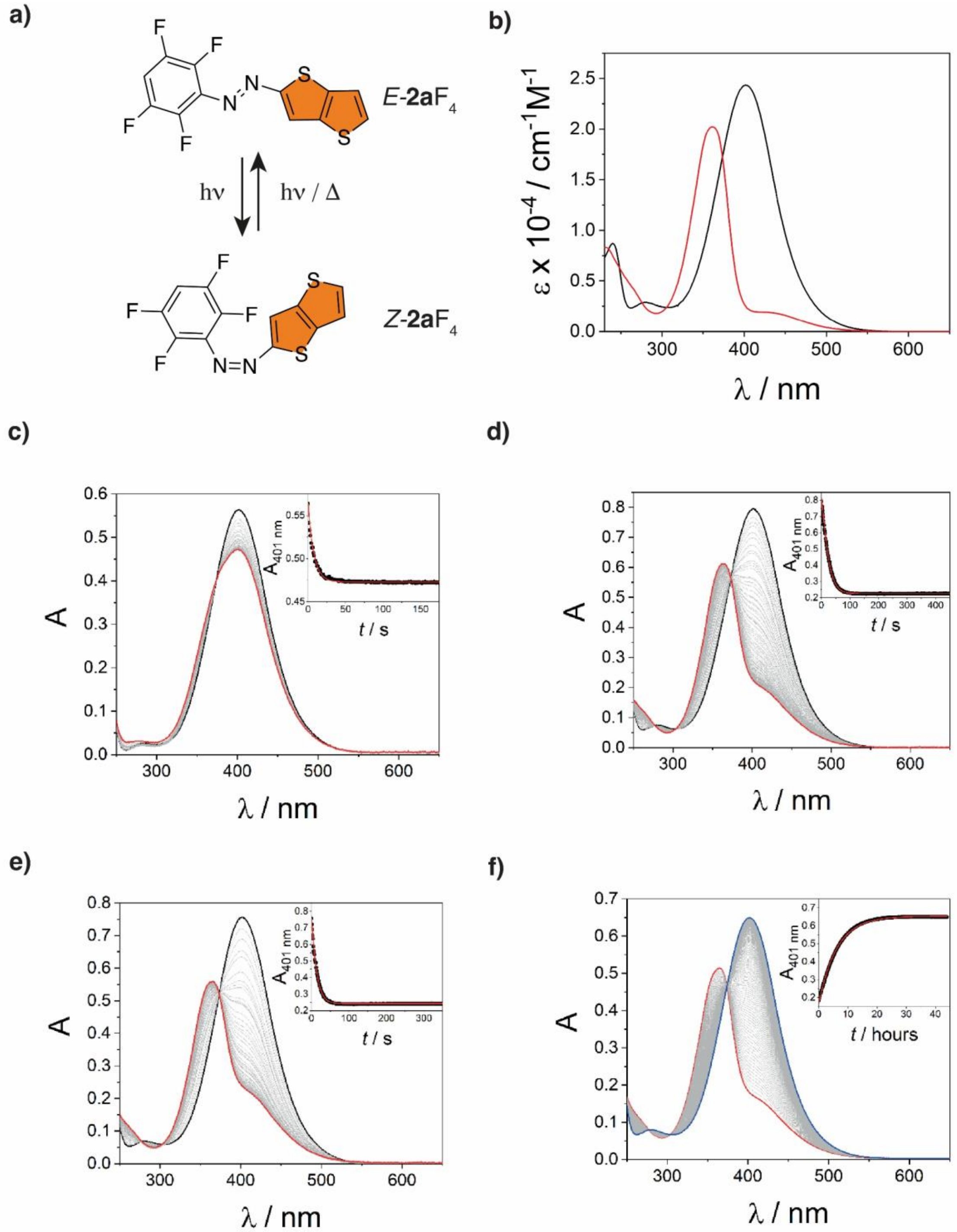

Figure S39. a) Photoisomerization reaction of *E*-**2a**F_4. b) Absorption spectra of the *E* (black line) and *Z* (computed by Fischer method, red line) isomers of compound **2a**F_4 in CH_2Cl_2. c) Absorption variations of a 2.3×10^{-5} M solution of *E*-**2a**F_4 in CH_2Cl_2 (black line) upon irradiation at 365 nm until PSS is reached (red line); inset: absorption changes at 401 nm (black dots) together with data fitting (red line). d) Absorption variations of a 3.4×10^{-5} M solution of *E*-**2a**F_4 in CH_2Cl_2 (black line) upon irradiation at 405 nm until PSS is reached (red line); inset: absorption changes at 401 nm (black dots) together with data fitting (red line). e) Absorption variations of a 3.2×10^{-5} M solution of *E*-**2a**F_4 in CH_2Cl_2 (black line) upon irradiation at 436 nm until PSS is reached (red line); inset: absorption changes at 401 nm (black dots) together with data fitting (red line). f) Thermal isomerization of a 2.7×10^{-5} M solution of *E*-**2a**F_4 in CH_2Cl_2 after exhaustive irradiation at 405 nm (red line) until total recovery of the *E* isomer (blue line); inset: absorption changes at 401 nm (black dots) together with data fitting (red line).

IX. Periodic DFT calculations on E-3a and E-3aPh$_3$ crystals

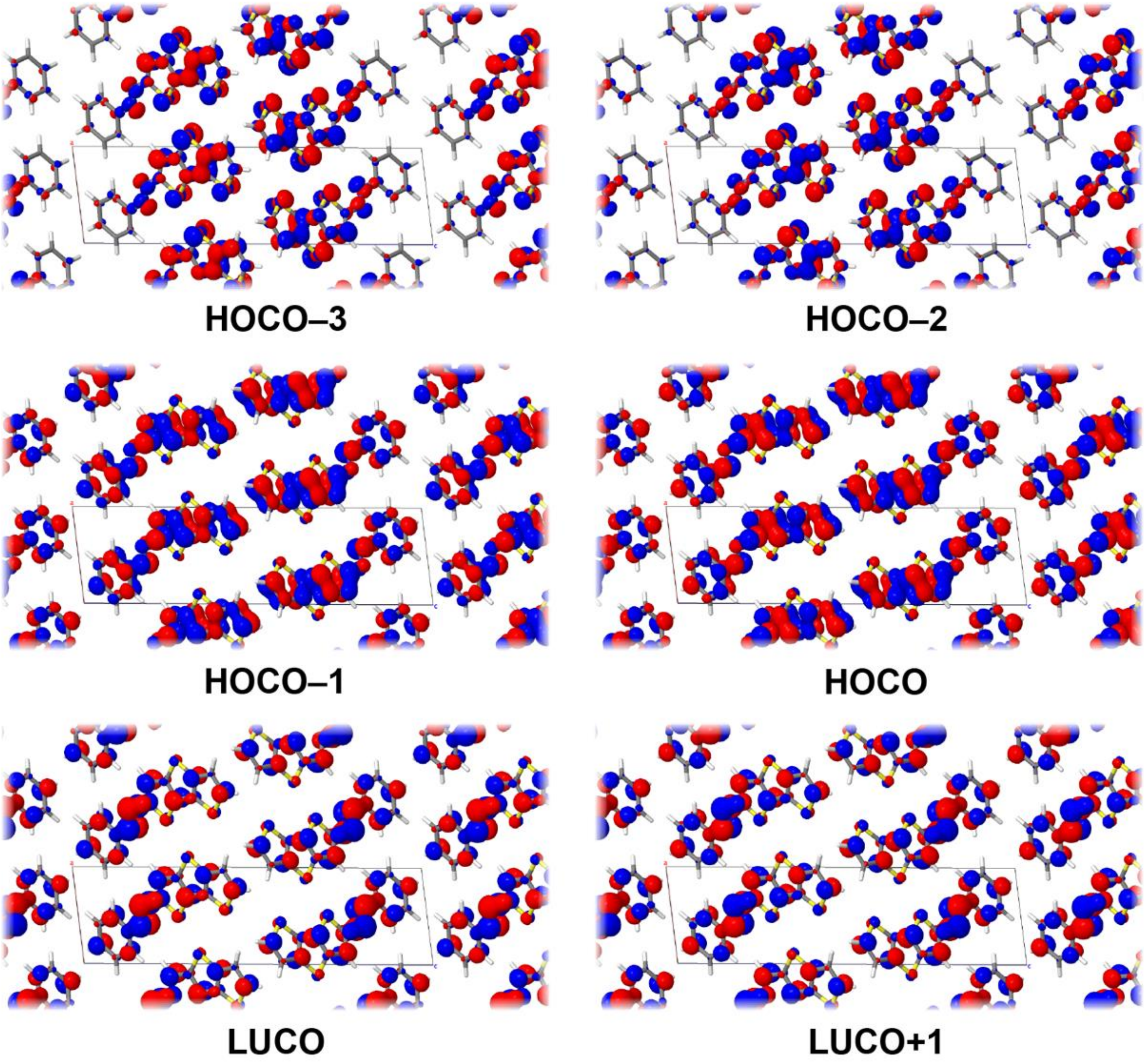

Figure S40. Frontier crystal orbitals of *E*-**3a** at the Γ point. HOCO = Highest occupied crystal orbital; LUCO = Lowest unoccupied crystal orbital. Since two *E*-**3a** molecules are present in the crystal cell, bands come in pairs of in- and out-of-phase combinations of molecular orbitals (*e.g.*, the linear combination of the two HOMOs generates HOCO and HOCO–1, the LUMO combination originates LUMO and LUCO+1).

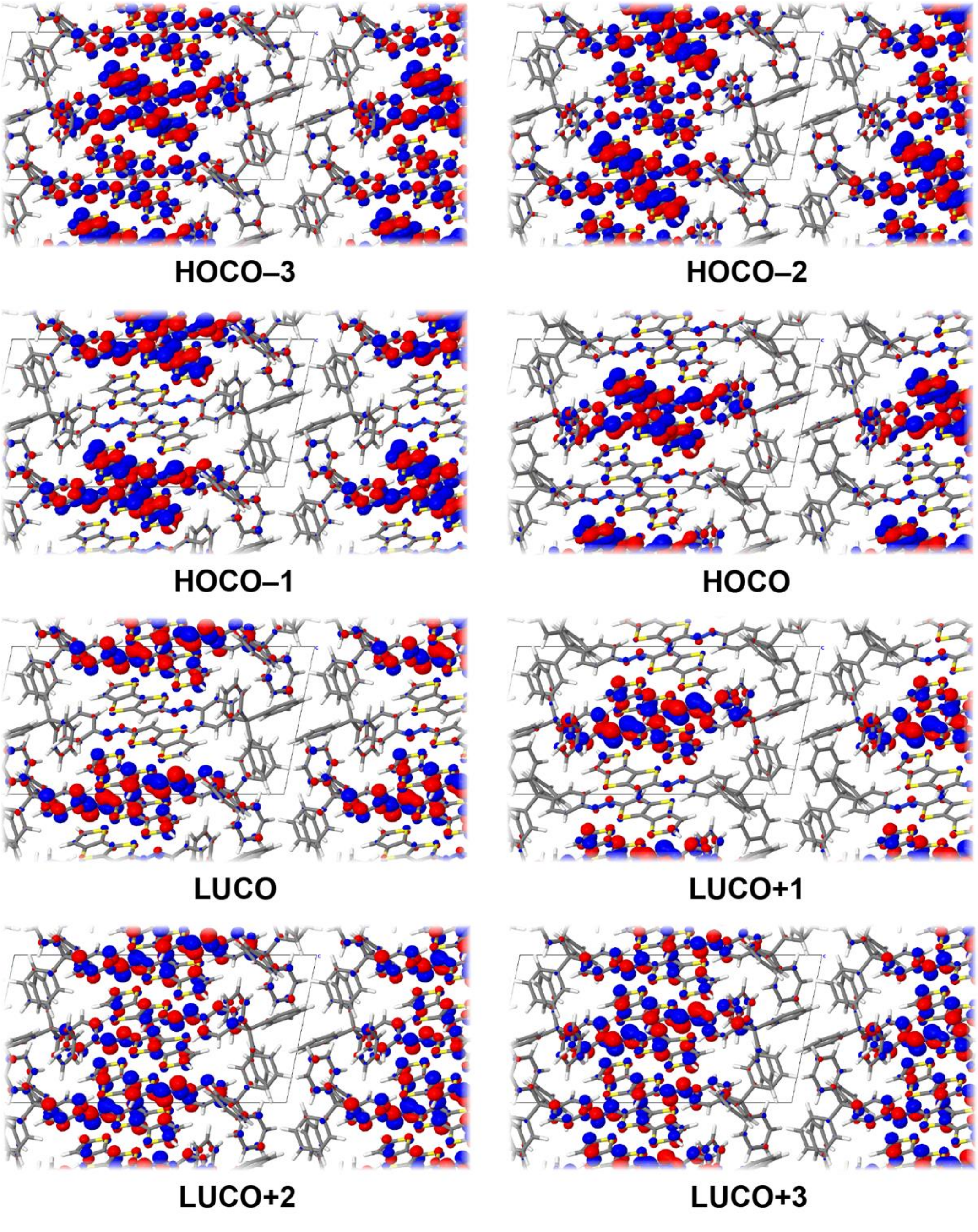

Figure S41. Frontier crystal orbitals of *E*-**3a**Ph_3 at the Γ point. HOCO = Highest occupied crystal orbital; LUCO = Lowest unoccupied crystal orbital. Since four *E*-**3a**Ph_3 molecules are present in the crystal cell, bands come in sets of four linear combinations of molecular orbitals (*e.g.*, HOMO coupling generates HOCO and HOCO–1, HOCO–2 and HOCO–3, the same for LUMO with LUCO to LUCO+3). Interestingly, it is evident that there are two couples of molecules in the crystal cell having stronger interactions within themselves (see main text).

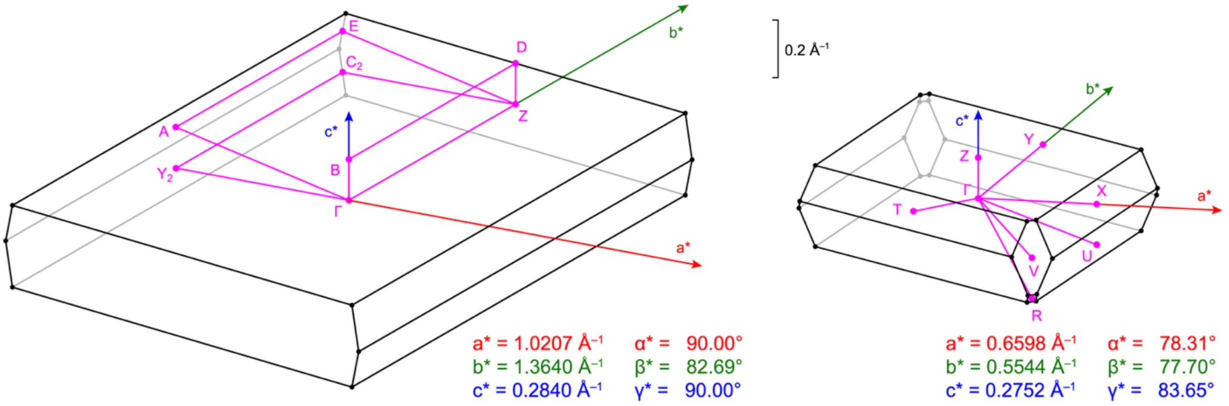

Figure S42. First Brillouin zone associated to the crystal cell of *E*-**3a** (left) and *E*-**3a**Ph3 (right), as experimentally determined by X-ray diffraction spectroscopy. The *k* paths mapped for generating the band-structure diagrams of Figure 6 c-d in the main text are highlighted in magenta, together with the relevant high-symmetry points. Reciprocal lattice parameters are also reported.

X. Solid-state characterization

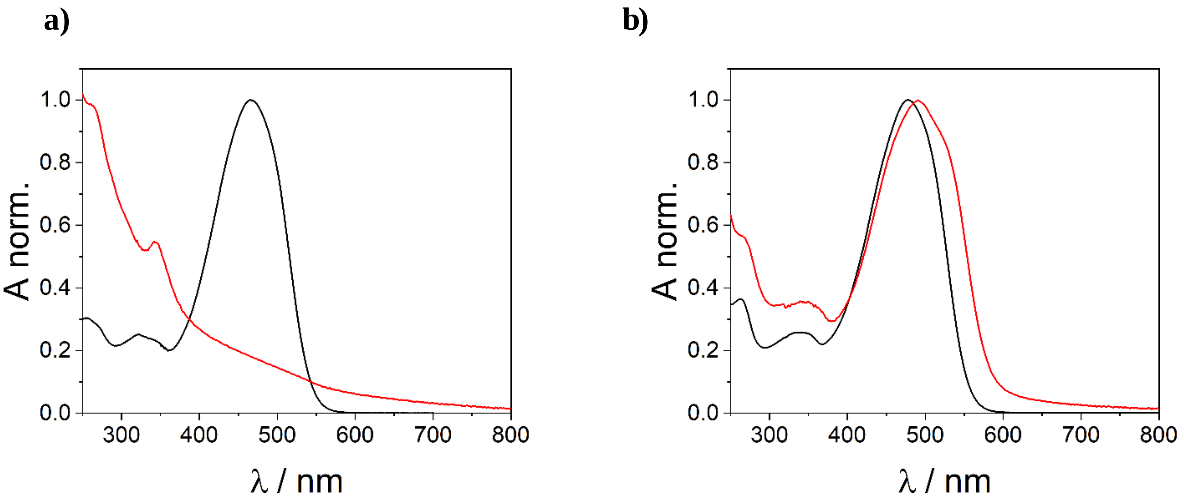

Figure S43. Comparison between absorption spectra in CH_2Cl_2 solution (black line) and spin-coated thin film (red line; thickness ≈ 60 nm, spin-coated from a 1 mg/mL solution at 1500 rpm) of compound *E*-**3** (a), *E*-**3**Ph_3 (b).

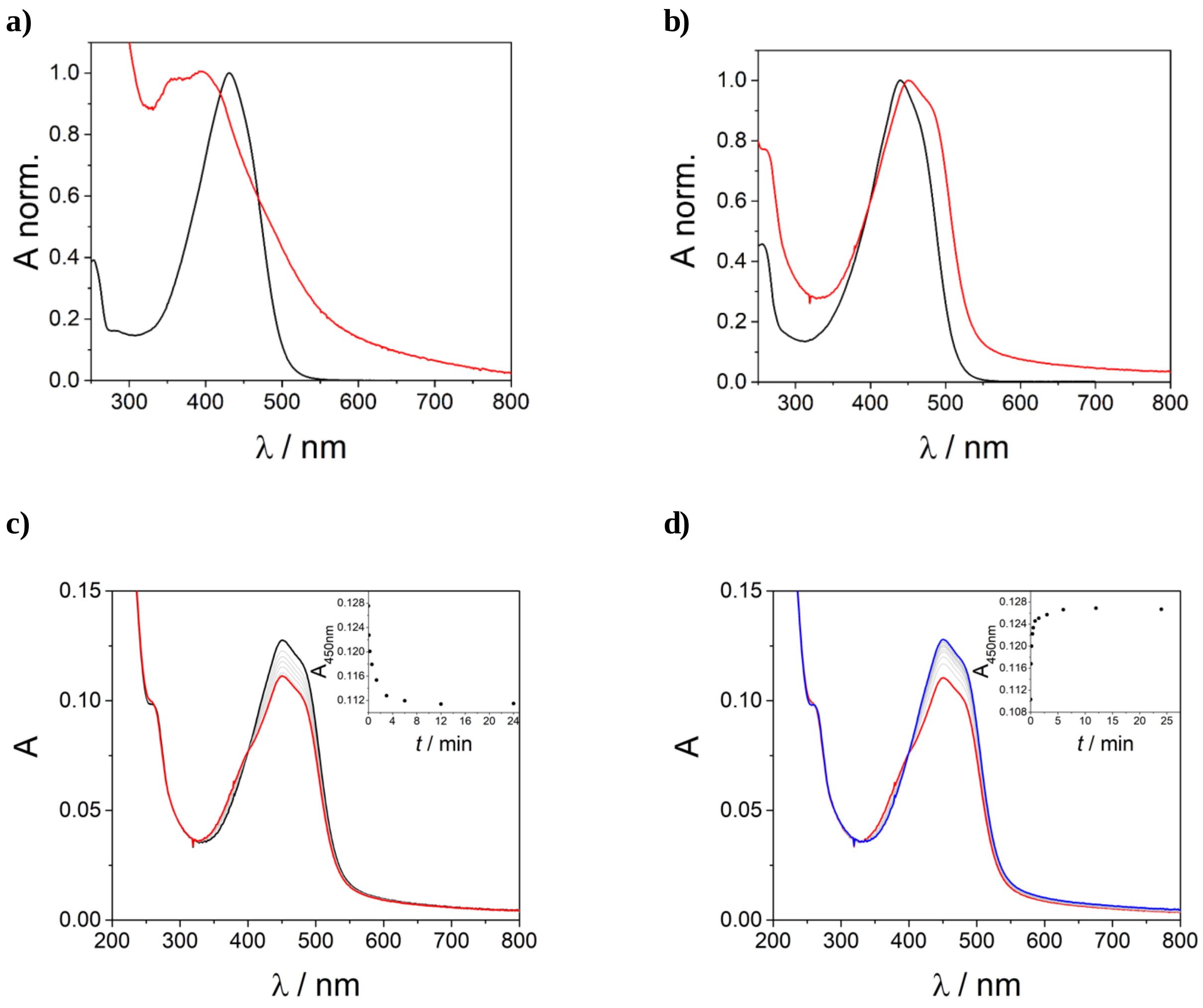

Figure S44. a) Comparison between absorption spectra in CH_2Cl_2 solution (black trace) and spin-coated thin film (red trace; thickness ≈ 60 nm, spin-coated from a 1 mg/mL solution at 1500 rpm) of compound *E*-**3a**. b) Comparison between absorption spectra in CH_2Cl_2 solution (black trace) and spin-coated thin film (red trace; thickness ≈ 60 nm, spin-coated from a 1 mg/mL solution at 1500 rpm) of compound *E*-**3a**Ph_3. c) Absorption variations of a spin-coated thin film of *E*-**3a**Ph_3 (black line) upon irradiation at 436 nm until PSS is reached (red line); inset: absorption changes at 450 nm (black dots). db) Absorption spectra of a spin-coated thin film of *Z*-**3a**Ph_3 (red line), after irradiation at 436 nm, upon irradiation at 365 nm until PSS (blue line); insert: absorption changes at 450 nm.

a)

b)

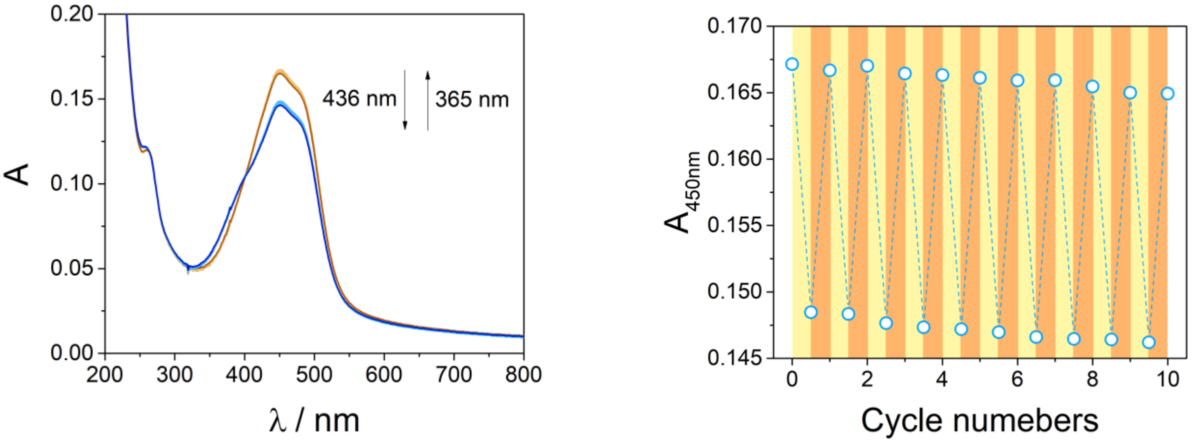

Figure S45. a) Absorption spectral changes of a thin solid film of *E*-**3a**Ph_3 upon repeated visible-light irradiation (436 nm for 20 min) and UV-irradiation (365 nm for 20 min). b) Absorption changes measured at 450 nm of a spin-coated thin film of *E*-**3a**Ph_3 subjected to visible (yellow areas) and UV-irradiation (orange areas) for 20 min each.

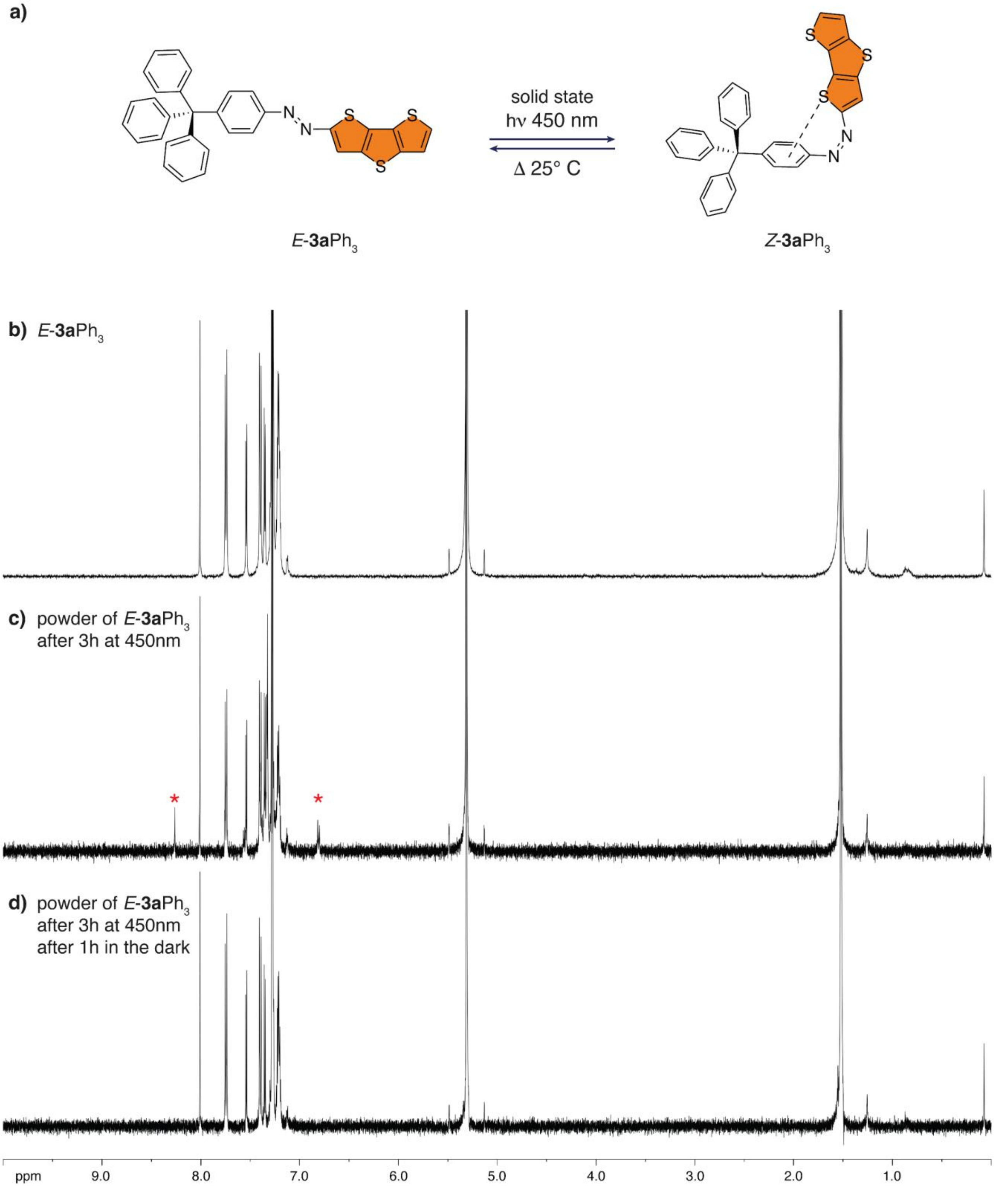

Figure S46. a) Schematic representation of the solid-state *E/Z* photoisomerization of **3a**Ph_3. b) ^{1}H NMR spectrum (500 MHz, CD_2Cl_2, 273K) of *E*-**3a**Ph_3 ($\approx 3 \times 10^{-3}$ M). c) ^{1}H NMR spectrum (500 MHz, CD_2Cl_2, 273K) of ca. 1 mg of *E*-**3a**Ph_3 solid powder after irradiation for 3 h at 450 nm (FWHM $\approx$ 40 nm; irradiance $\approx$ 20 mW cm^{-2}) upon dissolution in CD_2Cl_2. d) ^{1}H NMR spectrum (500 MHz, CD_2Cl_2, 273K) of ca. 1 mg of *E*-**3a**Ph_3 solid powder irradiated for 3 h at 450 nm, then dissolved in CD_2Cl_2 and kept in the dark at 25 °C for 1 h before analysis. Diagnostic integrals for protons of *Z*-**3a**Ph_3 observed upon dissolution of the irradiated powder are marked with red asterisks in panel c.

XI. AFM images and Data Analysis

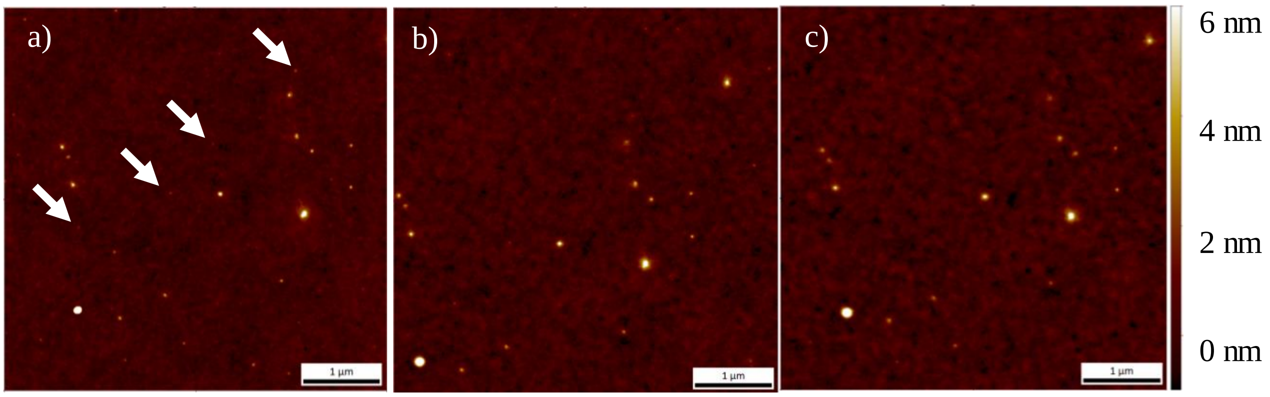

Figure S47. AFM morphology images of the same area of a spin coated thin film of *E*-**3a**Ph_3 (thickness ≈ 60 nm): a) pristine; b) after illumination for 2 hours at 436 nm (≈ 20 mW·cm^{-2}); c) after additional illumination for 2 hours at 365 nm (≈ 20 mW·cm^{-2}). White arrows in a) indicate clusters that become less visible and eventually disappear upon light exposure.

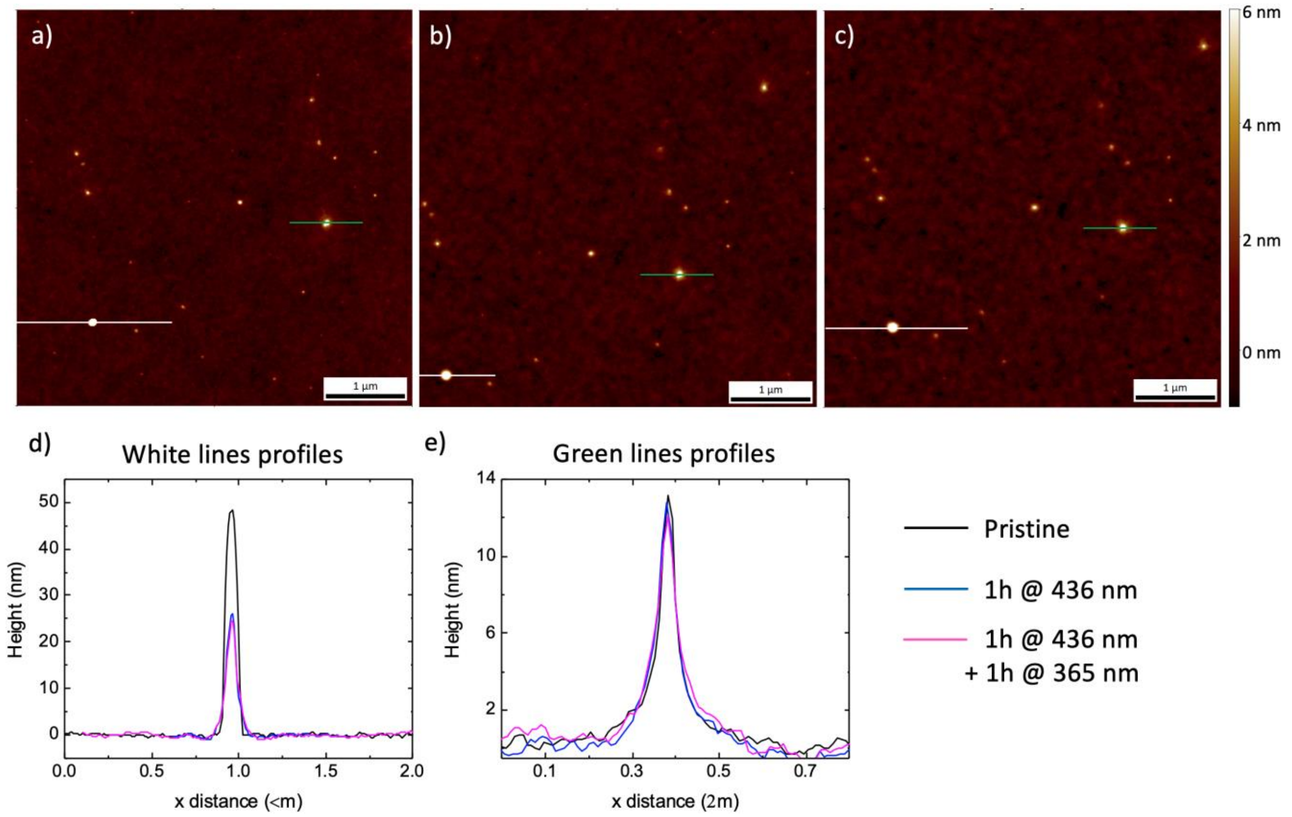

Figure S48. AFM morphology images of the same area of a spin coated thin film of *E*-**3a**Ph_3 (thickness ≈ 60 nm): a) pristine; b) after illumination for 2 hours at 436 nm (≈ 20 mW·cm^{-2}); c) after additional illumination for 2 hours at 365 nm (≈ 20 mW·cm^{-2}). d, e): evolution of the line profiles (white lines shown in d) and green lines in e) of representative nanoclusters.

Table S8. Roughness parameters as calculated from the images in **Figure S42**

Image	***Ra*** [nm]	***Rq*** [nm]
*Pristine thin film of E-**3a**Ph$_3$*	0.27	0.39
After 2h @ 436 nm	0.29	0.43
After 2h @ 436 nm and 2h @ 365 nm	0.29	0.49

XII. Optical microscopy

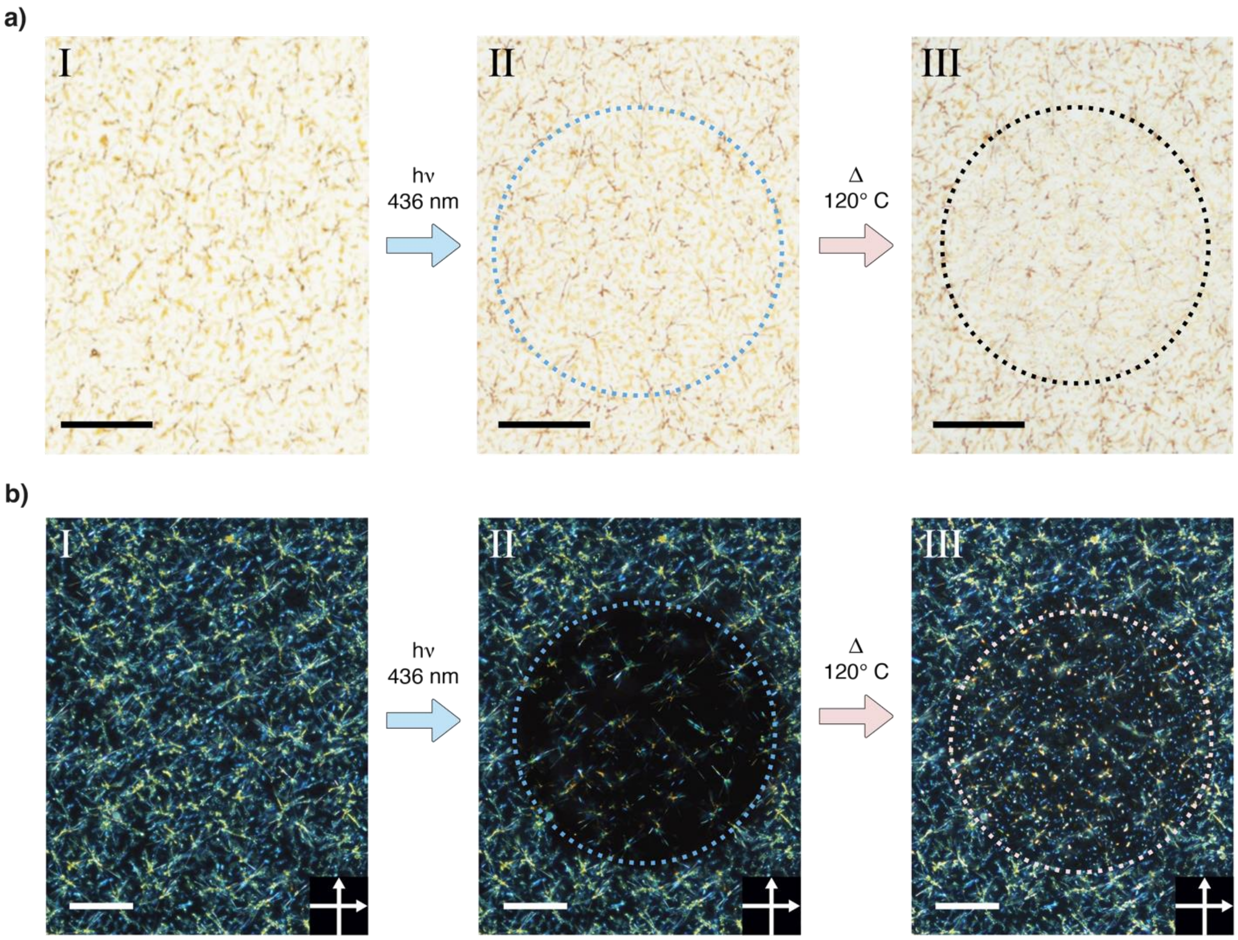

Figure S49. Micrographs of drop casted film of *E*-**3a**Ph3 on glass slides a) under bright field illumination, and b) under cross polarizers. Before (I), after localized (central spot indicated by the dotted circle) irradiation at 436 nm (II), and after thermal annealing at 120° C for 20 min (III). *Scale bar*: 30 µm. The white arrows represent the relative orientation of the polarizer and analyser

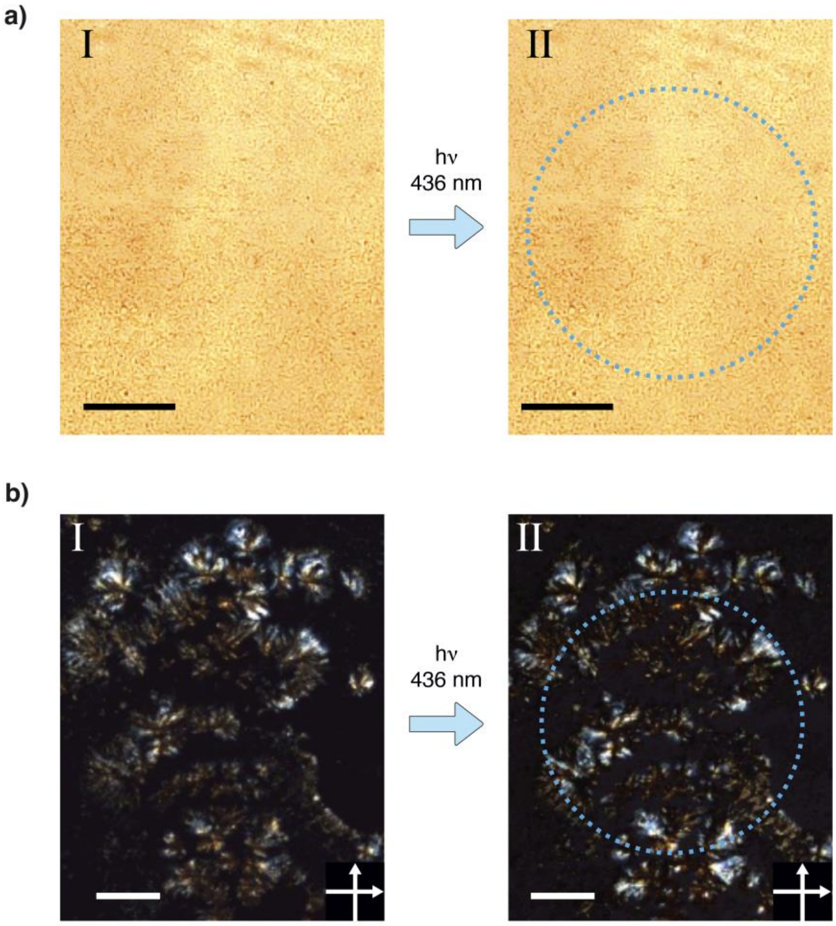

Figure S50. Micrographs of: (a) spin coated thin film of *E*-**3a** under bright field illumination, and (b) drop casted film under cross polarizers: before (I), and after localized irradiation (central spot indicated by the dotted circle) at 436 nm (II). *Scale bar*: 30 μm. The white arrows represent the relative orientation of the polarizer and analyser. The absence of intensity and geometric differences between micrographs I and II highlights the absence of significant photoinduced morphological transformations in *E*-**3a** upon light irradiation in the solid-state.

XIII. Single crystal structures determination

Table S9. Crystal data and experimental details for *E*-**3a** and *E*-**3a**Ph_3

	E-3a	*E-3aPh₃*
Formula	$C_{14}H_8N_2S_3$	$C_{33}H_{22}N_2S_3$
Fw	300.40	542.70
T, K	100(2)	100(2)
λ, Å	0.71073	1.54178
Crystal symmetry	Monoclinic	Triclinic
Space group	$P2_1$	P-1
a, Å	6.2060(5)	9.7711(5)
b, Å	4.6066(4)	11.6021(5)
c, Å	22.3042(17)	23.7771(11)
α, °	90	100.630(2)
β,°	97.308(2)	101.294(2)
γ, °	90	94.041(2)
Cell volume, $Å^3$	632.47(9)	2582.0(2)
Z	2	4
D_c, Mg m^{-3}	1.577	1.396
$\mu(Mo\text{-}K_\alpha)$, mm^{-1}	0.570	2.825 [$\mu(Cu\text{-}K_\alpha)$]
F(000)	308	1128
Crystal size/ mm	0.28 x 0.15 x 0.10	0.07 x 0.05 x 0.05
θ limits, °	3.547 to 28.252	3.872 to 68.563
Reflections collected	9713	106017
Unique obs. Reflections [$F_o > 4\sigma(F_o)$]	3087 [R(int) = 0.0225]	9471 [R(int) = 0.0738]
Goodness-of-fit-on F^2	1.225	1.042
R_1 (F)[a], wR_2 (F^2) [I > 2σ(I)][b]	0.0254, 0.0666	0.0486, 0.1409
Largest diff. peak and hole, e. $Å^{-3}$	0.340 and -0.262	0.474 and -0.687

[a])$R_1 = \Sigma||F_o|-|F_c||/\Sigma|F_o|$. [b] $wR_2 = [\Sigma w(F_o^2-F_c^2)^2/\Sigma w(F_o^2)^2]^{1/2}$ where $w = 1/[\sigma^2(F_o^2) + (aP)^2 + bP]$ where $P = (F_o^2 + F_c^2)/3$.

Crystallographic data have been deposited with the Cambridge Crystallographic Data Centre (CCDC) as supplementary publication number CCDC 2479090-2479091 for *E*-**3a** and *E*-**3a**Ph_3, respectively.
Copies of the data can be obtained free of charge *via* www.ccdc.cam.ac.uk/getstructures

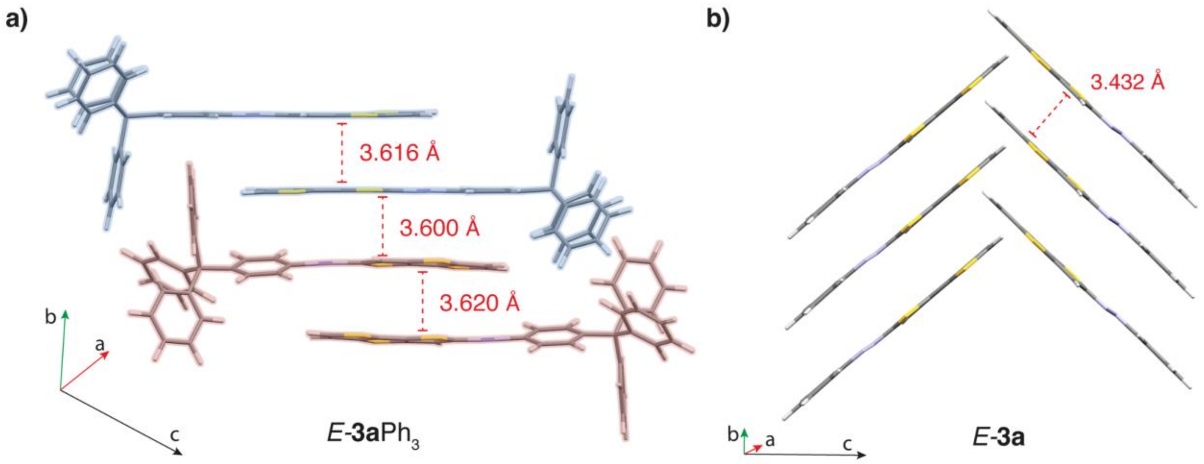

Figure S51. Single crystal X-ray structures highlighting the most relevant π-π stacking interactions for (a) *E*-**3a**Ph_3 (the two distinct molecular conformers are highlighted in red and light blue, respectively), and (b) *E*-**3a**.

XIV. Devices fabrication and characterization

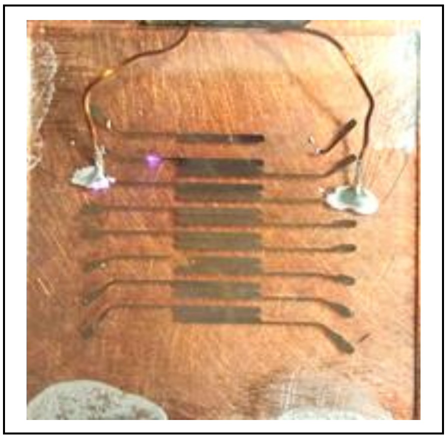

Figure S52. Contacts pattern of the device used for the *E*-**3a**Ph_3 and *E*-**3a** film photoswitching characterization: onto a 25mm x 25mm plasma-pre-treated 2mm thick glass, 300nm thick Ag-electrodes were evaporated under high vacuum through a suitable mask. Electrodes' interspace amounts to 100 µm. Afterwards, a ≈ 60 nm thick *E*-**3a**Ph_3 (or *E*-**3a**) film was spin-coated over the electrodes to cover only their central region.

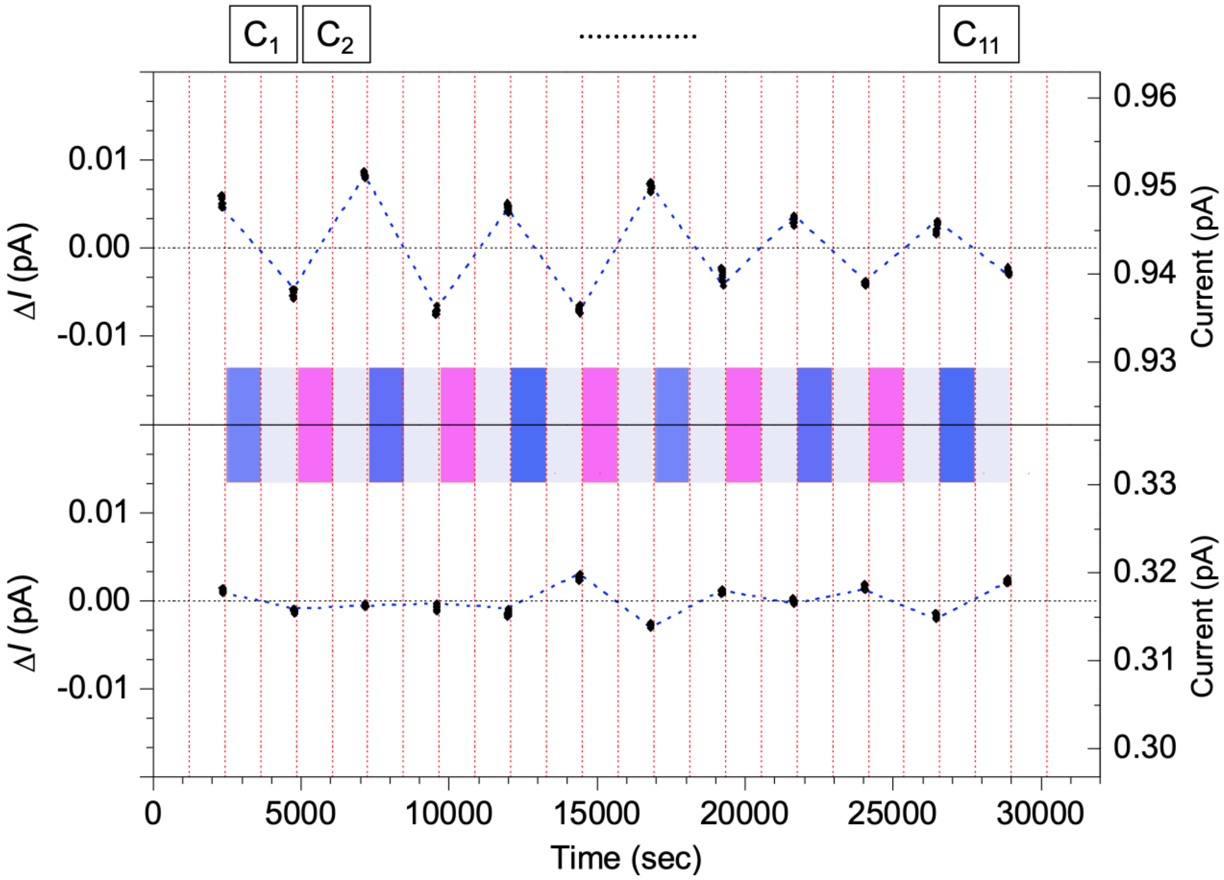

Figure S53. Current response under a fixed 0.1 V bias of planar two-contact devices (N ≥ 3 devices) fabricated from ~ 60 nm thick films of *E*-**3a**Ph_3 (top) and *E*-**3a** (bottom), measured in the dark (gray bars) 20 minutes after repeated cycles of alternating irradiation for 20 minutes at 436 nm (blue bars) and 365 nm (magenta bars). Left scale: relative current change (ΔI) with respect to a guide for the eye (horizontal dotted line).

XV. References

[1] M. Montalti, A. Credi, L. Prodi, et al., *Handbook of photochemistry*, 3rd ed., CRC Press, **2006**.
[2] E. Fischer, *J. Phys. Chem.* **1967**, 71, 3704-3706.
[3] Neese, F., Software Update: The ORCA Program System − Version 6.0. *WIREs Comput Mol Sci*, **2025**, 15, e70019. https://doi.org/10.1002/wcms.70019.
[4] M. Garcia-Ratés, F. Neese, *J Comput Chem.* **2020**, 41, 922–939.
[5] A. Najibi, L. Goerigk, *J Comput Chem.* **2020**; 41, 2562–2572.
[6] W. Florian, A. Reinhart, *Phys. Chem. Chem. Phys.*, **2005**,7, 3297-3305.
[7] D. Jacquemin, B. Mennucci, C. Adamo, *Phys. Chem. Chem. Phys.*, **2011**,13, 16987-16998.
[8] S. Hirata, M. Head-Gordon, *Chem. Phys. Lett.*, **1999**, 314, 291-299.
[9] a) F. Neese, F. Wennmohs, A. Hansen, U. Becker, *Chem. Phys.*, **2009**, 356, 98-109. b) T. Petrenko, S. Kossmann, F. Neese, *J. Chem. Phys.* **2011**; 134 (5): 054116. c) B. Helmich-Paris, B. de Souza, F. Neese, R. Izsák, *J. Chem. Phys.* 2021, 155 (10): 104109.
[10] a) F. Neese, A. Hansen, D. G., *J. Chem. Phys.* **2009**, 131 (6), 064103. b) F. Neese, F. Wennmohs, A. Hansen, *J. Chem. Phys.*, **2009**, 130 (11), 114108. c) C. Riplinger, F. Neese, *J. Chem. Phys.*, **2013**; 138 (3), 034106.
[11] F. Weigend, R. Ahlrichs, *Phys. Chem. Chem. Phys.*, **2005**, 7, 3297-3305.
[12] a) A. Hellweg, C. Hättig, S. Höfener, *et al.*, *Theor Chem Acc.*, **2007**, 117, 587–597. b) F. Weigend, *Phys. Chem. Chem. Phys.*, **2006**, *8*, 1057-1065.
[13] M. Garcia-Ratés, U. Becker, F. Neese, *J. Comput. Chem.*, **2021**, 42(27), 1959.
[14] R. L. Martin, *J. Chem. Phys.*, **2003**; 118 (*11*), 4775–4777.
[15] A. H. Heindl, H. A. Wegner, *Chem. Eur. J.*, **2020**, *26*, 13730.
[16] S. Axelrod, E. Shakhnovich, R. Gómez-Bombarelli, *ACS Cent. Sci.*, **2023**, 9, *2*, 166–176.
[17] F. Aleotti, L. Soprani, L. F. Rodríguez-Almeida, *et. al.*, *Mol. Syst. Des. Eng.*, **2025**, *10*, 13-18.
[18] C. Kollmar, K. Sivalingam, B. Helmich-Paris, C. Angeli, F. Neese., *J. Comput. Chem.* **2019**, 40, 1463–1470.
[19] A. Hellweg, C. Hättig, S. Höfener, *et al.*, *Theor Chem Acc*, **2007**, 117, 587–597.
[20] a) C. Kollmar, K. Sivalingam, Y. Guo, F. Neese, *J. Chem. Phys.*, **2021**; 155 (*23*), 234104. b) Y. Guo, K. Sivalingam, F. Neese, *J. Chem. Phys.*, **2021**; 154 (*21*), 214111.
[21] A. Erba, J. K. Desmarais, S. Casassa, B. Civalleri, *et. al.*, J. *Chem. Theory Comput.*, **2023**, 19, *20*, 6891–6932.
[22] A. V. Krukau, O. A. Vydrov, A. F. Izmaylov, G. E. Scuseria, *J. Chem. Phys.*, **2006**; 125 (*22*), 224106.
[23] D. Vilela Oliveira, J. Laun, M. F. Peintinger, T. Bredow., *J. Comput. Chem.*, **2019**, *40*, 2364–2376.
[24] W. Humphrey, A. Dalke, K. Schulten, *J. Mol. Graphics*, **1996**, 14(*1*), 33–38.
[25] Jmol: an open-source Java viewer for chemical structures in 3D. http://www.jmol.org/
[26] L. Tian Lu, *J. Chem. Phys.*, **2024**; 161 (*8*), 082503.
[27] APEX3 Software Package V2019; Bruker AXS Inc.: Madison, WI, **2019**.
[28] Bruker SAINT, v8.40A: Part of the APEX3 Software Package V2019; Bruker AXS Inc.: Madison, WI, **2019**.
[29] Bruker SADABS V2016/2: Part of the APEX3 Software Package V2019; Bruker AXS Inc.: Madison, WI, **2019**.
[30] G. M. Sheldrick, SHELXT − Integrated Space-Group and Crystal-Structure Determination. Acta Crystallogr., Sect. A: Found. Adv. **2015**, 71, 3−8.
[31] G. M. Sheldrick, Crystal Structure Refinement with SHELXL. Acta Crystallogr., Sect. C: Cryst. Struct. Commun. **2015**, 71, 3−8.
[32] C.F. Macrae, I. Sovago, S.J. Cottrell, P.T.A. Galek, P. McCabe, E. Pidcock, M. Platings, G.P. Shields, J.S. Stevens, M. Towler P.A. Wood, "*Mercury 4.0*: from visualization to analysis, design and prediction", *J. Appl. Cryst.*, **2020**, ***53***, 226.
[33] Lampert, M.A.; Park, P. *Current Injection is Solids*; Academic Press, New York, 1970.
[34] Y. Mazaki, K. Kobayashi, *Tetrahedron Lett.* **1989**, 30, 3315-3318.
[35] S. Liu, H. Zhang, Y. Li, J. Liu, L, Du, M. Chen, R. T. K. Kwok, J. W. Y: Lam, D. L. Phillips, B. Z. Tang, *Angew. Chem. Int. Ed.* **2018**, 57, 15189-15193.
[36] U. Folli, D. Iarossi, M. Montorsi, A. Mucci, L. Schenetti, *Chem. Soc.*, *Perkin Trans.* **1995**, 1, 537-540.
[37] I. E. Palamà, F. Di Maria, I. Viola, E. Fabiano, G. Gigli, C. Bettini, G. Barbarella, *J. Am. Chem. Soc.* **2011**, 133, 17777-17785.
[40] P. S. Gribanov, M. A. Topchiy, Y. D. Golenko, Y. I. Lichtenstein, A. V. Eshtukov, V. E. Terekhov, A. F. Asachenko, M. S. Nechaev, *Green Chem.*, **2016**, *18*, 5984–5988.
[41] M. R. Smith III, T-Y. Cheng, G. L. Hillhouse *Inorg. Chem.*, **1992**, *31*, 1535–1538.
[42] K. Traskovskis, I. Mihailovs, A. Tokmakovs, A. Jurgis, V. Kokars, M. Rutkis, *J. Mater. Chem.*, **2012**, *22*, 11268–11276.
